# Purple spot damage dynamics investigated by an integrated approach on a 1244 A.D. parchment roll from the Secret Vatican Archive

**DOI:** 10.1038/s41598-017-05398-7

**Published:** 2017-09-07

**Authors:** Luciana Migliore, Maria Cristina Thaller, Giulia Vendittozzi, Astrid Yazmine Mejia, Fulvio Mercuri, Silvia Orlanducci, Alessandro Rubechini

**Affiliations:** 1 Department of Biology, Tor Vergata University, Rome, Italy; 2 Department of Natural Resources, Inter-American Development Bank, Tegucigalpa, Honduras; 3 Department of Industrial Engineering, Tor Vergata University, Rome, Italy; 4 Department of Chemical Science and Technology, Tor Vergata University, Rome, Italy; 5 Vatican Secret Archives, Vatican City, Vatican City

## Abstract

Ancient parchments are commonly attacked by microbes, producing purple spots and detachment of the superficial layer. Neither standard cultivation nor molecular methods (DGGE) solved the issue: causative agents and colonization model are still unknown. To identify the putative causal agents, we describe the 16 S rRNA gene analysis (454-pyrosequencing) of the microbial communities colonizing a damaged parchment roll dated 1244 A.D. (*A.A. Arm. I-XVIII 3328*, Vatican Secret Archives). The taxa in damaged or undamaged areas of the same document were different. In the purple spots, marine halotolerant Gammaproteobacteria, mainly Vibrio, were found; these microorganisms are rare or absent in the undamaged areas. Ubiquitous and environmental microorganisms were observed in samples from both damaged and undamaged areas. Pseudonocardiales were the most common, representing the main colonizers of undamaged areas. We hypothesize a successional model of biodeterioration, based on metagenomic data and spectroscopic analysis of pigments, which help to relate the damage to a microbial agent. Furthermore, a new method (Light Transmitted Analysis) was utilized to evaluate the kind and entity of the damage to native collagen. These data give a significant advance to the knowledge in the field and open new perspectives to remediation activity on a huge amount of ancient document.

## Introduction

**Figure 1:**
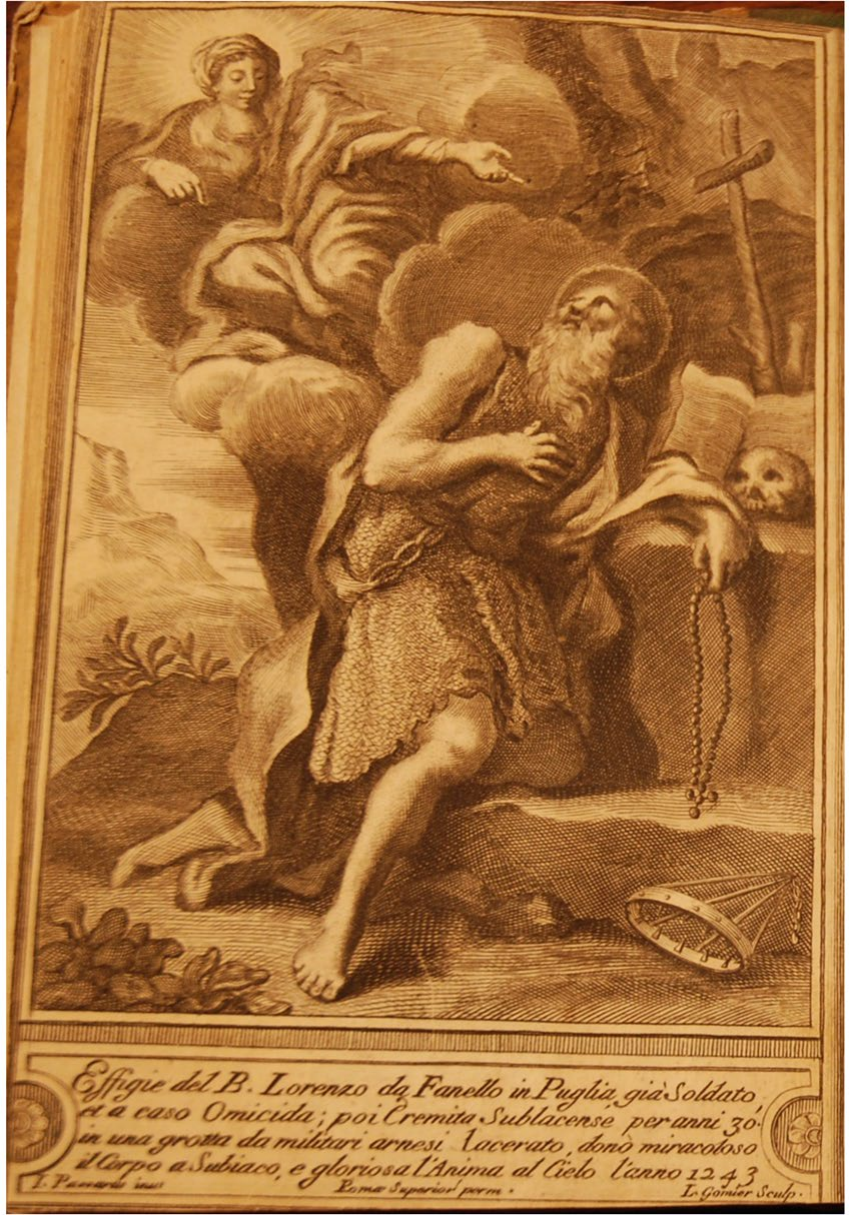
A portrait of Laurentius Loricatus (engraving from G. da Capistrano, 1805). On the left, S. Chelidonia, on the right Lorenzo da Fanella, also known as Laurentius Loricatus.

**Figure 2:**
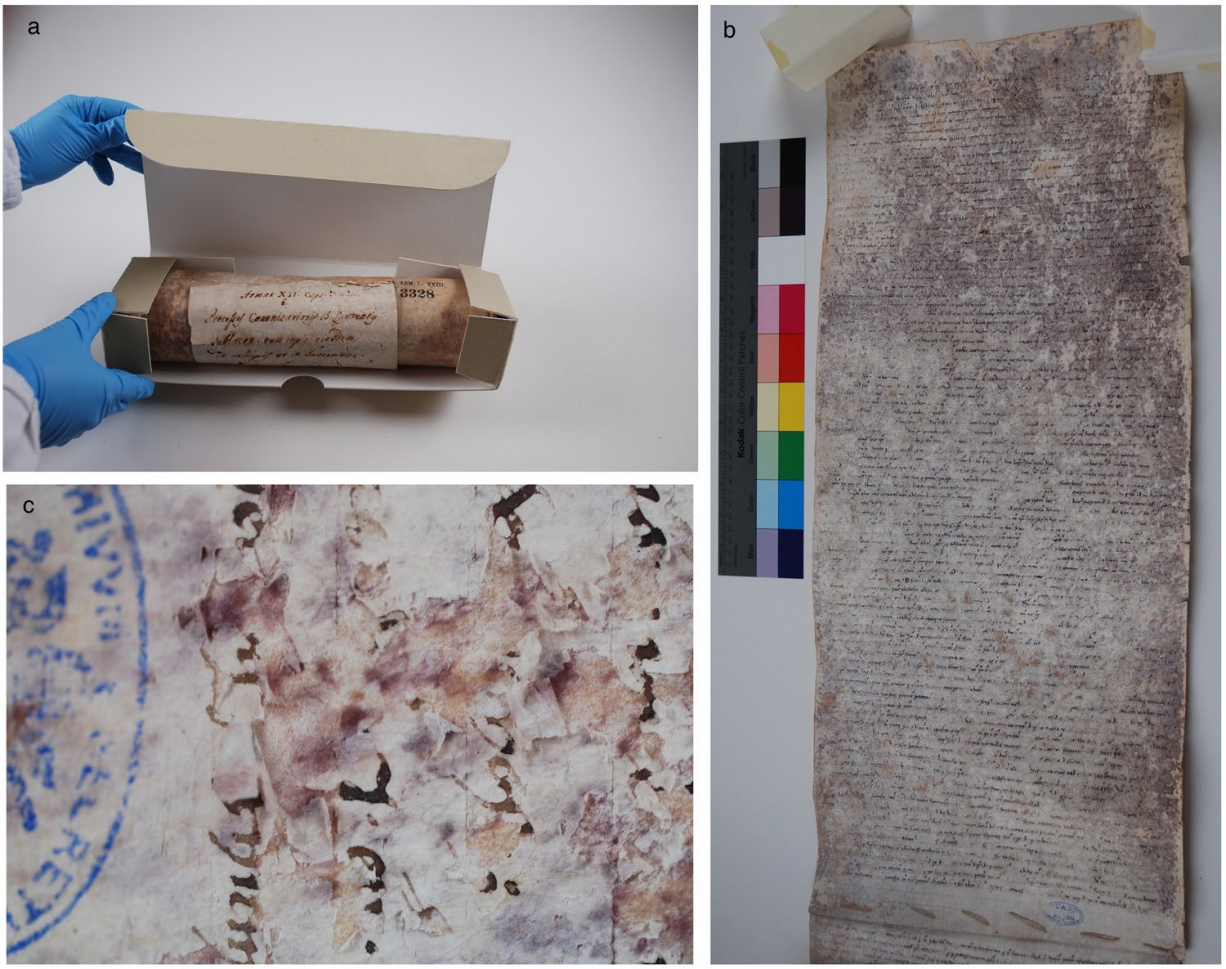
The *A.A. Arm. I-XVIII 3328* parchment. (**a**) The roll in its box before its restoration; (**b**) the first page of the roll; (**c**) close-up of the damaged roll with purple spots and deteriorated surface layer on the flesh side.

Laurentius Loricatus (Fig. 1) was a young soldier who, at the age of 15–16 (1205–6 A.D.) accidentally killed a man. To expiate he retired in a cave near Subiaco (Rome, Italy) for 34 years, self-flagellating and wearing instruments of penance. His story was written in 1244 A.D. on a 5 meters long parchment roll, reporting the investigations for his possible canonization. The roll is a goat parchment, the *A.A. Arm. I-XVIII 3328* (Fig. 2a) belonging to the oldest collection of the Archives (*Fondo* “*Archivum Arcis”*); it is stored at the Vatican Secret Archives, since the end of the 18^th^ Century.

Parchments are made from animal skin (in ancient times in Italy, usually goat or sheep, and in northern countries usually cow; R. Tinaburri, personal communication); hence, they are mainly constituted of collagen. This parchment roll shows the most frequent microbial alterations affecting ancient parchments^1, 2^, such as: isolated or coalescent purple spots, with a nucleated peripheral halo, more crowded on the flesh side. Within the spots, the collagen structure is damaged and, often, the superficial layer at the flesh side is detached. The consequent loss of readability is significant (Fig. 2c). The hair side is practically unaffected as it is observed in the great majority of parchments^3^. The damaged areas of the *A.A. Arm. I-XVIII 3328* parchment roll are: (i) the first and the last page of the document, which was completely covered by the purple spots (Fig. 2b), and (ii) the lateral margins of the internal pages in the entire document, from the edge of the parchment towards the written area, spanning from 2 to 5–6 cm. The damage of the roll most likely happened before it was moved to the Vatican Secret Archives (at the end of the 18^th^ Century), where it is now kept under controlled environmental conditions (50 ± 2% Relative Humidity, 20 ± 2 °C Temperature).

Due to the widespread expansion of this kind of damage, often present on very important ancient documents, several studies have tried to understand its causes. Marconi^4^ unsuccessfully tried an *in vitro* culturing strategy, by inoculating new parchments with purple spot material. Gallo and Strzelczyk^5^ utilized standard cultivation methods without any successful result. More recently, Piñar *et al*.^2^ applied molecular methods (DGGE fingerprinting and sequencing of 50 clones of a 16S library), to detect the archaeal, bacterial and fungal communities colonizing a damaged parchment. None of the several identified microorganisms has been unambiguously considered as a putative causal agent, and no unambiguous source of biodeteriogen microorganisms has been identified: Authors attributed colonization to air-dispersion, to animals acting as vectors, or to direct inoculation by human handling.

The aims of this study are: to identify the putative causal agents, hypothesize a successional model of biodeterioration and clarify the damage done by biodeteriogens to the structure of parchment collagen. To this purpose, different approaches were used: metagenomic data by NGS (Next Generation Sequencing) to identify the microbial communities and Raman analysis of the purple pigment, to relate the damage to a microbial agent, and last but not least, a new Light Transmitted Analysis (LTA), described later on, which reveals the kind and entity of damage suffered by the native collagen.

To our best knowledge, NGS techniques, as 454-pyrosequencing, have never been applied to the study of ancient parchment documents damaged by the purple spots, except for a preliminary study on the same parchment written in Italian and published on the journal of the Vatican Secret Archives^6^. In this last study, a clear differentiation between bacterial colonizers in the purple damaged and undamaged samples was found, such as the presence of halotolerant microorganisms only in the purple damaged samples.

The rapid and non-destructive Raman spectroscopy provides structural information on both organic and inorganic molecular compounds and it is extensively used in cultural heritage to identify pigments, inks, minerals and substrata^7^. Due to its high sensitivity in detecting carotenoids and a large variety of other microbial pigments^8, 9^, in this study it has been utilized to supply the chemical identification of the microbial pigments in the purple spots, a hitherto little explored use on cultural heritage.

The deterioration degree of the parchment can be assessed by different methods, some of them based on microimaging techniques^10^, some others on the analysis of the thermal stability of the collagen fibers^11, 12^. The Micro Hot Table (MHT) analysis investigates the fibers shrinkage induced by collagen denaturation, and quantifies the so-called shrinkage temperature Ts, associated with its hydrothermal stability^13^. The Differential Scanning Calorimetry (DSC), instead, is based on the measure of enthalpy changes associated with the denaturation process. It can provide a detailed picture of the parchment deterioration state and information on the collagen populations with different thermal stability^14, 15^ but does not allow the visual monitoring of the fibers during their transformation. In this study a new suitable method, based on the analysis of the light transmitted by the sample during its denaturation, has been used. Developed starting from a formerly presented technique^16^, it combines some capabilities of both the calorimetry and the shrinkage analysis. In particular, it enables the analysis of both the thermal stability and the heterogeneity of the different collagen populations, and the simultaneous recording of microscopic images, which describe the morphological evolution of the fibers during the shrinkage process.

Hence, the aim of this study was to identify the “culprits”, the putative causative agents of the purple spots damage on the parchment roll *A.A. Arm. I-XVIII 3328*, by characterizing both the microbial communities associated with the spots and the chemical composition of the purple stains. This information is the basis to assess the microbial colonization model leading to this kind of damage and to evaluate the deterioration pattern of the parchment structure (collagen fibers).

## Results

### Microbial analysis

**Figure 3:**
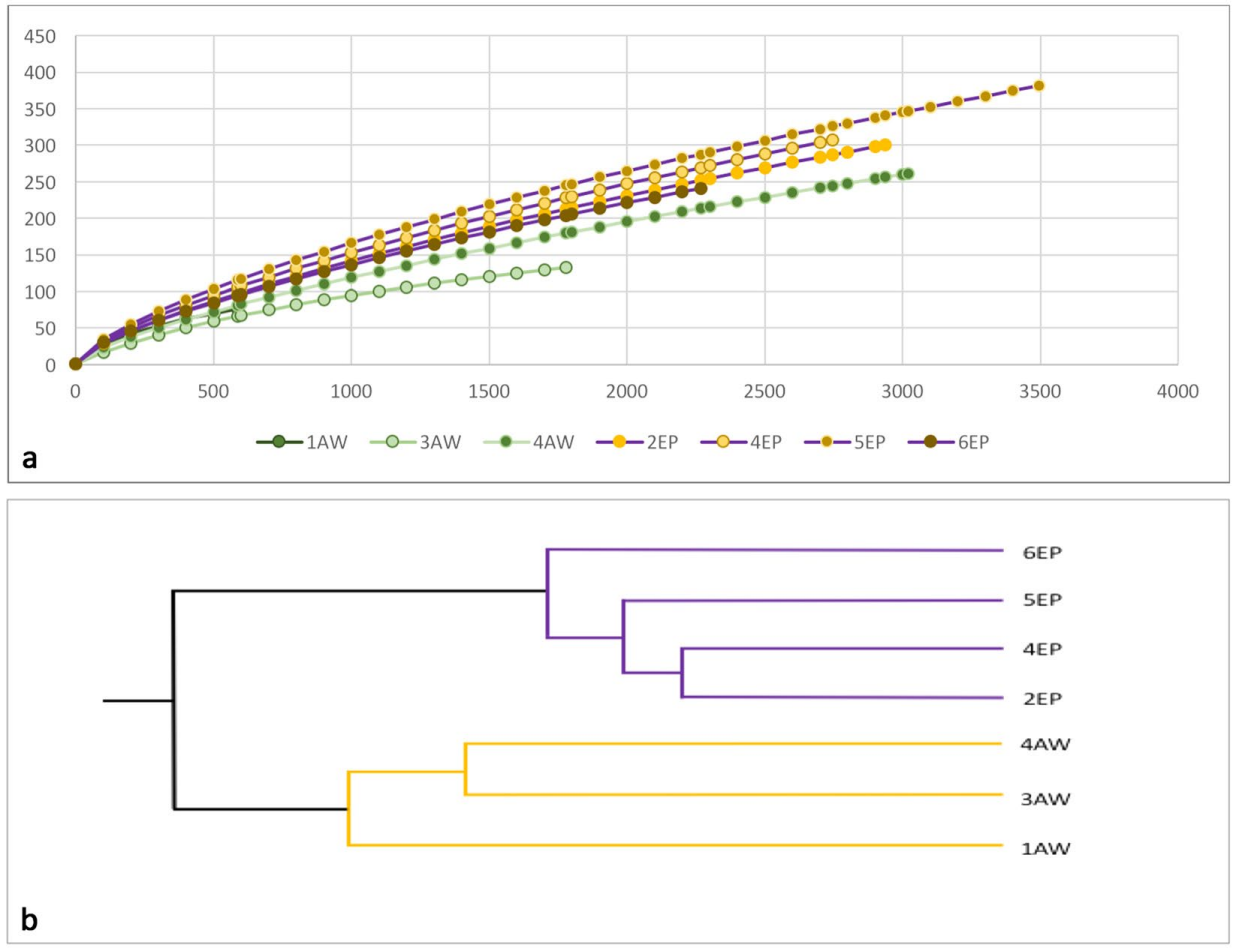
Differences in the microbial communities. (**a**) Rarefaction curves of the bacterial communities associated with 4 purple damaged (#EP) and 3 uncolored undamaged (#AW) samples of the parchment. Bray Curtis dissimilarity: (**b**) UPGMA tree of the purple damaged (#EP) and uncolored undamaged (#AW) samples (UniFrac.weighted, WScore = 1; WSig = <0.001). The UniFrac methods describe whether two or more communities have the same structure. The significance of the test statistic indicates the probability that the communities have the same structure by chance. The value does not indicate a level of similarity.

Analysis performed with MOTHUR revealed a total of 16,829 bacterial and 1 archaea sequences which were assigned to a total of 1,224 OTUs. On average, the purple spot damaged samples yielded 11,445 bacterial sequences (sample yield ranging from 2,267 to 3,496), and the uncolored undamaged samples 5,384 bacterial sequences (sample yield ranging from 588 to 3,019). To standardize differences among samples, the dataset was normalized, by random subsampling, to a common depth of 588 sequence reads per sample. Rarefaction curves were built to evaluate differences in sampling effort (Fig. 3a).

Diversity, as calculated by the Shannon diversity index (H’) was higher in the purple damaged samples than in the undamaged uncolored ones, 3.18 ± 0.15 *vs*. 2.12 ± 0.76, respectively.

The analysis of bacterial community structure, based on the OTUs abundances (Fig. 3b), showed a clear separation between the communities found on the two kinds of samples. ANOSIM and UniFrac highlighted the statistical differences between samples (normalized dataset - ANOSIM: p-value 0.025; UniFrac: p < 0.001). 140 of the 1,224 total OTUs (11.4%) were shared in the uncolored and purple sets. The unshared OTUs in the purple samples accounted for the 85.4% out of the 957 OTUs (11,445 sequences) and for the 65.6% out of the 407 OTUs (5,384 sequences) in the uncolored samples (see also Supporting Fig. S1).

The complete list of OTUs collected from both damaged and undamaged samples is reported in Supporting Table S1. Actinobacteria and Gammaproteobacteria were dominant, the prevalent orders were Pseudonocardiales and Vibrionales in undamaged and purple damaged areas, respectively. The main Alphaproteobacteria orders were Rhizobiales and Rhodobacterales, while in Firmicutes were Bacillales, mainly the family *Staphylococcaceae*, and Lactobacillales (mainly *Streptococcaceae*). Deltaproteobacteria and Epsilonproteobacteria were quite absent. Only one archaeal OTU was found (sample 4AW), belonging to Pacearchaeota.

**Figure 4:**
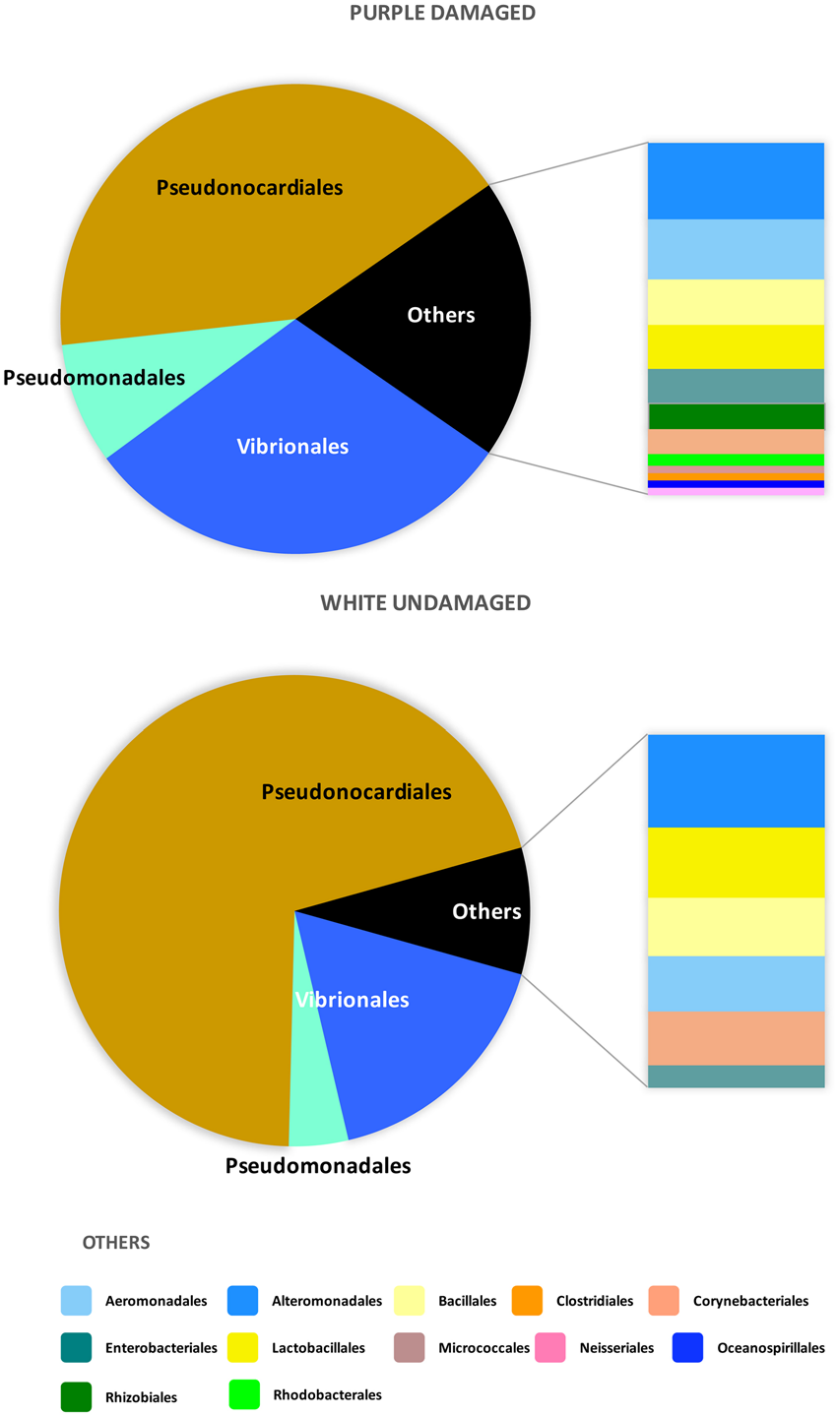
Composition of the microbial communities. Mean number of sequences per taxonomic group in the purple damaged (#EP) and uncolored undamaged (#AW) samples of the parchment.

The Fig. 4 shows how the entire set of OTUs is partitioned among damaged and undamaged samples. The relative abundance of the OTUs in each set of the damaged and undamaged samples depicts the ‘core microbiome’ composition of the two set of samples; here again, the main difference concerns the ratio, differences being driven by the orders Vibrionales and Pseudonocardiales.

In the undamaged, uncolored samples Pseudonocardiales was the dominant order (68.2% of the identified sequences), it dominated two of the samples (1AW and 3AW, corresponding to 81.6% and 91.2% of the sequences, respectively); whilst the percentage was lower in the third replicate (4AW, 54.9%), collected closely near a damaged area. In the damaged purple samples, Pseudonocardiales accounted for 41.4%, ranging from 32.3% (4EP) to 48.3% (2EP). Vibrionales are abundant in both the sample sets, accounting for 16.4% of the sequences in the uncolored undamaged samples and 29.7% in the purple damaged ones. In these last samples, the 78.7% of the Gammaproteobacteria were halotolerant aquatic or marine bacteria, like Vibrionales (62.6%), Alteromonadales (8.6%), Aeromonadales (6.5%), Oceanospirillales and Chromatiales (1%). Among them, the most frequent genera were: *Aliivibrio, Allomonas* (*Vibrio fluvialis*)*, Photobacterium* and *Vibrio* (including *Listonella*), but also *Aeromonas*, *Halomonas, Marinomonas, Pseudoalteromonas*, *Rheinheimera, Shewanella* (details in Supporting Table S1)

### Chemical analysis

**Figure 5:**
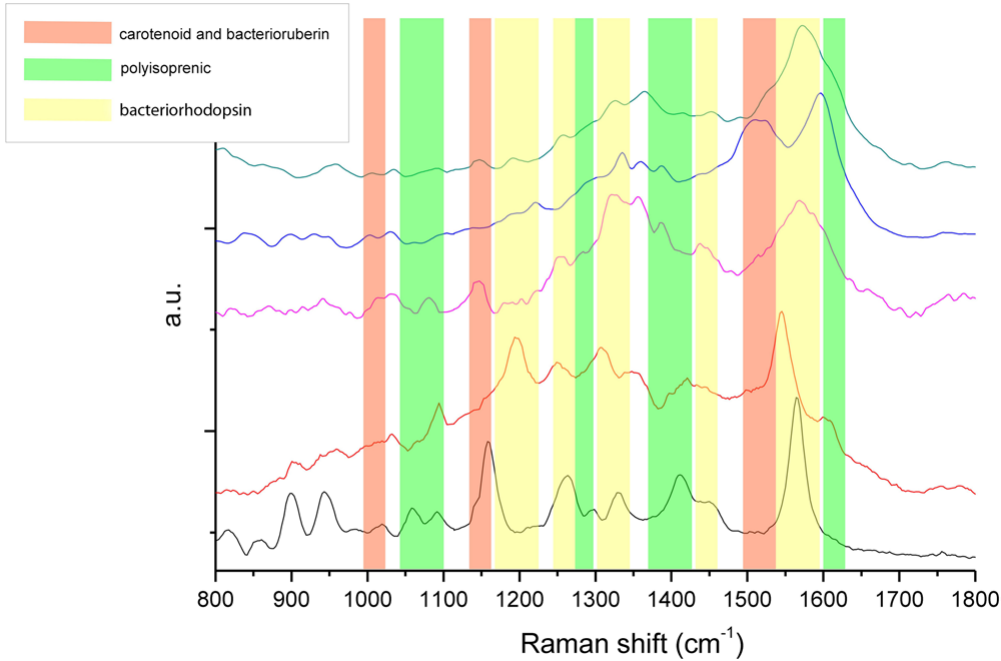
Raman spectra of different purple sampling points on Si substrate.

Raman spectra of chemically extracted pigments from damaged areas (Fig. 5), exhibited several bands and a complex shape, due to the contribution of several biomolecules; specific patterns of bands represent the fingerprint of specific biomolecules and/or functional groups. The lipid pigments extracted from purple samples were assigned to: bacteriorhodopsin (yellow area), isoprenoid derivatives (green area) and carotenoid and bacterioruberin (a 50-carbon open chain carotenoid; orange area)^17^. Raman spectra of bacteriorhodopsin or rhodopsin-like pigments were dominated by the signal of retinal isomers, whose dominant feature in the Raman spectra is a complex strong band in the 1575 cm^−1^ region. Other intense bands were located at about 1260, 1320 and 1450 cm^−1^, all of these are related to deformation modes of -CH_3_ and -CH_2_. In the case of carotenoid, C = C stretching is in the region of 1520 cm^−1^, C-C stretching around 1150 cm^−1^ and C-CH bending around 1000 cm^−1^; in the case of bacterioruberin the C = C stretching mode is located at 1506 cm^−1^.

The best represented Raman spectra in the samples were: a chromophore of retinal isomers, the bacteriorhodopsin, and small poly-isoprene molecules, maybe originated from larger carotenoids.

Carotenoids have two strong Raman bands due to in-phase *v*_1_(C = C) and *v*_2_(C-C) stretching vibrations of the polyene chain, and a feature of medium intensity corresponding to the in-plane rocking modes of the CH_3_ groups, attached to the polyene chain. The wavenumber positions of both *v*_1_ and *v*_2_ bands depend on the length of the polyene chain, a longer conjugated polyene chain causing a significant red shift of *v*_1_ band position.

However, aggregation state and chemical substitutions can strongly affect the bands positions, which can result shifted, if compared to those of the pure molecules. As a consequence, the identification of specific carotenoids in ancient complex organic matrix is particularly difficult and Raman technique cannot provide unambiguous recognition.

### Denaturation analysis

**Figure 6:**
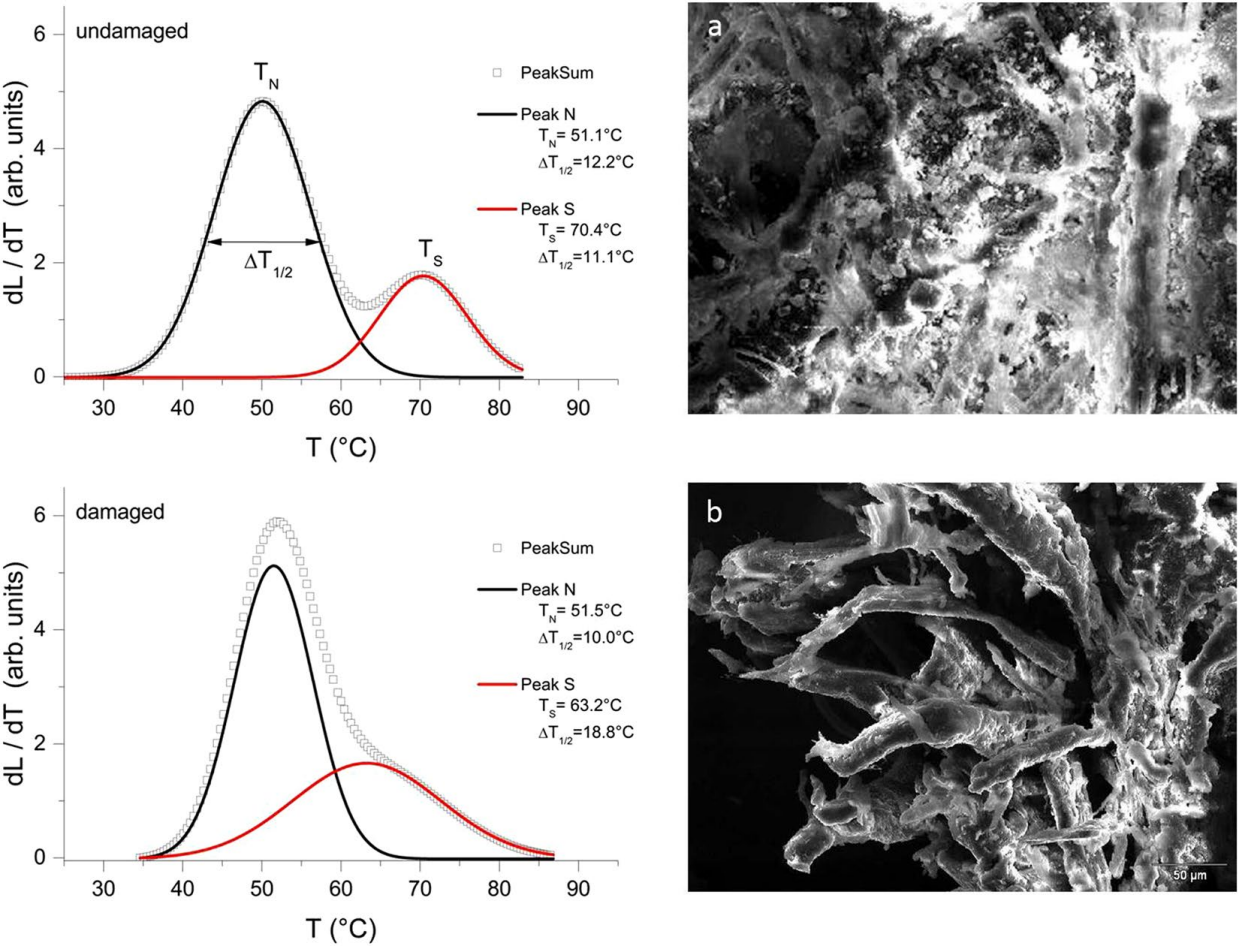
Structural alterations of the parchment. *Left*: Light Transmitted Analysis (LTA) curves. In both graphs, the grey curve corresponds to the best fit of the quantity dL/dT, derivative of the light transmitted signal amplitude L as a function of temperature, describing the strength of the overall process of hydrothermal denaturation. Each curve has been deconvoluted in two different peaks associated with the denaturation of different collagen populations having different thermal stability. The peak temperatures T_N_ and T_S_ provide information about the thermal stability of the populations, the higher the peak temperature, the higher is the stability. The full width at half maximum of the peaks T_1/2_ is related to the homogeneity of the collagen in terms of thermal stability, and therefore to the homogeneity of its preservation state; the lower the homogeneity, the larger is the peak width. *Right*: Physical appearance of parchment structure (SEM, 350x); (**a**) undamaged parchment, a diffused matrix encompassing the more robust fibers is clearly evident; (**b**) damaged sample, the encompassing matrix is completely absent in the damaged portions of the parchment.

The denaturation analysis of the collagen populations highlighted clear differences between purple (damaged) and uncolored (undamaged) parchment samples (Fig. 6). The analysis showed a slightly different behavior of the low temperature peak (black solid line), describing the denaturation of the so-called *stable native* collagen (N). This collagen is mainly located in the core region of the more robust fibers, representing the frame structures of the parchment^18^. On the other hand, the differences between the two set of samples were significant in the high temperature peak (red solid line) associated with the denaturation of the *stabilized* collagen, S, located mainly in the sheath of the big fibers^18^.

In the damaged parchment, N presented larger T_N_ (+1.5 °C) and smaller ΔT_1/2_ (−2.2 °C) than the undamaged sample, while S had a lower T_S_ (−7.2 °C) and increased ΔT_1/2_ (+7.7 °C). These responses can be explained by the different arrangement of collagen in undamaged and damaged samples. As shown in SEM images (Fig. 6, right): the diffused matrix encompassing the more robust fibers in the undamaged parchment samples is completely absent in the damaged portions of the parchment. The results suggest that the collagen intermingled in between the big fibers is essentially *native stable*, but slightly less stable and homogeneous than that, also *native*, in the core region of the more robust fibers. In fact, in the damaged samples the small increase of the N peak temperature and the decrease of its width account for a more stable and homogeneous residual N population that, after the removal of the inter-fibers network, mainly consists of the N collagen protected in the core of the fibers. The decrease of the T_S_ peak and the increase of its width depended on the sheath of the big fibers, more exposed to the attack of the bacteria.

## Discussion

The common purple damage of ancient parchments has been studied by comparing damaged and undamaged areas from the same document (the roll named *A.A. Arm. I-XVIII 3328*). An integrated multidisciplinary approach was adopted, making use of molecular, chemical and physical techniques. To the best of our knowledge these techniques have never been used in combination to analyze the purple spots damage of parchments.

This multidisciplinary approach yielded a great amount of interesting information, useful to decipher what the parchment suffered during its 800 years-long story. The new Light Transmitted Analysis (LTA) technique allows to study and localize the structural damages without introducing any bias caused by the sample preparation: in the purple spots, it showed that the alteration mainly affects the collagen matrix, saving the larger fibers. The Raman specter analysis provided clues about the bacterial pigments at the basis of the purple discoloration, detecting microbial rhodopsins: the well-known archaeal bacteriorhodopsin^19^ and/or the more recently discovered proteorhodopsins^20^, which are largely diffused in aerobic marine bacteria^21–23^. The Next Generation Sequencing (NGS) approach, yields a much deeper resolution of complex microbiota than other molecular methods. The primer pair used in this study (515F/806R, employed in the Earth Microbiome Project; www.earthmicrobiome.org) allow the identification of a wide range or microorganisms. Although Hugerth *et al*.^24^ pointed out that these primers miss some archaeal phyla, a test performed with the Probe Match tool in RDP^25^ revealed that they match the Euryarchaeota (515F = 0.95; 806R = 0.95) and even better the Halobacteria, which were actually looked for, according to the Raman results (515F = 0.97 and 806R = 0.96). As expected, the NGS output highlighted significantly different communities in the two sets of samples. The taxonomical assignation of the OTUs adds even more information, depicting the relative frequencies of the different bacterial phyla and orders. Moreover, a BLAST analysis revealed that OTUs find their best homologues within both cultured and uncultured microorganisms from environmental samples (mainly salt-related or soil ones), and human (mainly skin-associated). A detailed description of the best homologies for the OTUs occurring more than five times is shown in Supporting Table S2.

Evidences linking bacteria to the degradation process include: (i) some rhodopsins-producing microorganisms, responsible of the purple discoloration; (ii) the damage of the parchment surface linked to rarefaction and/or disappearance of the collagen matrix; (iii) the microbial community structure, actually different in the damaged and undamaged portions of the parchment; *i.e*. Actinobacteria, mainly Pseudonocardiales, dominant in the undamaged areas of the parchment, while Proteobacteria, mainly Gamma, with the prevalence of Vibrionales, prevailing in the purple spots, although Actinobacteria were also abundant.

As a whole, these results can be explained in the framework of a successional model.

### A conspiracy in several acts: a microbial succession is the model of colonization

Parchments are made from animal skins, more precisely they consist of the dermal skin layer only. The procedure for their preparation have remained the same in the course of the centuries. Since ancient times, to reach the final thickness and smoothness, skin was subjected to a series of treatments to prevent putrefaction (sea salt, sodium chloride), to remove the hair (lime, calcium hydroxide), to allow ink attachment, and for the whitening and/or smoothening of the surface^26^. The salt treatment seems not have been usually used in the northern regions^27^.

In the Southern coastal regions (e.g. Italy) or whenever it was cheap and available, sea salt was used to preserve the hides, until processed, soon after the animal were slaughter^2, 28, 29^. Salting was carried out dry or in tanks, where the skins were plunged in brine for some days^30^ so that the salt ions entered deeply into the skin. It is easy to suppose that the brine could also act as a culture/enrichment medium for salt carried halophilic and halotolerant microorganisms (archaea, marine bacteria and their resting phases), in ancient times surely present in the marine salt. After liming and soaking in water, the entire skin was then placed on a stretching frame, deeply scraped and shaved until the thin final feature was obtained. Hence, it is possible to hypothesize that at the end of the production procedures, the parchment internal environment was salty, with a gradient of decreasing salinity from the outer layers of the animal skin inwards. In this environmental conditions, halophilic archaea and halotolerant bacteria, located into the reticular collagen structure, were both preserved.

Once in the monasteries, the parchments were stored in the *armaria* ^31^ along the cloisters walls, exposed to moisture, changing temperature, and light, whenever they were unrolled in the reading rooms. Hence, parchment environment probably changed with time and local events, enabling the growth of the microbial colonizers, which were already present in the parchment. As regard the *A.A. Arm. I-XVIII 3328* parchment, it belongs to the oldest collection of the Archives, called *Fondo “Archivum Arcis”*, which was kept in Castel S. Angelo (downtown Rome) until the end of the XVIII Century. In ancient times Castel S. Angelo was exposed to frequent and important floods of the Tiber River^32^ which could have reached the library and wet the roll.

**Figure 7:**
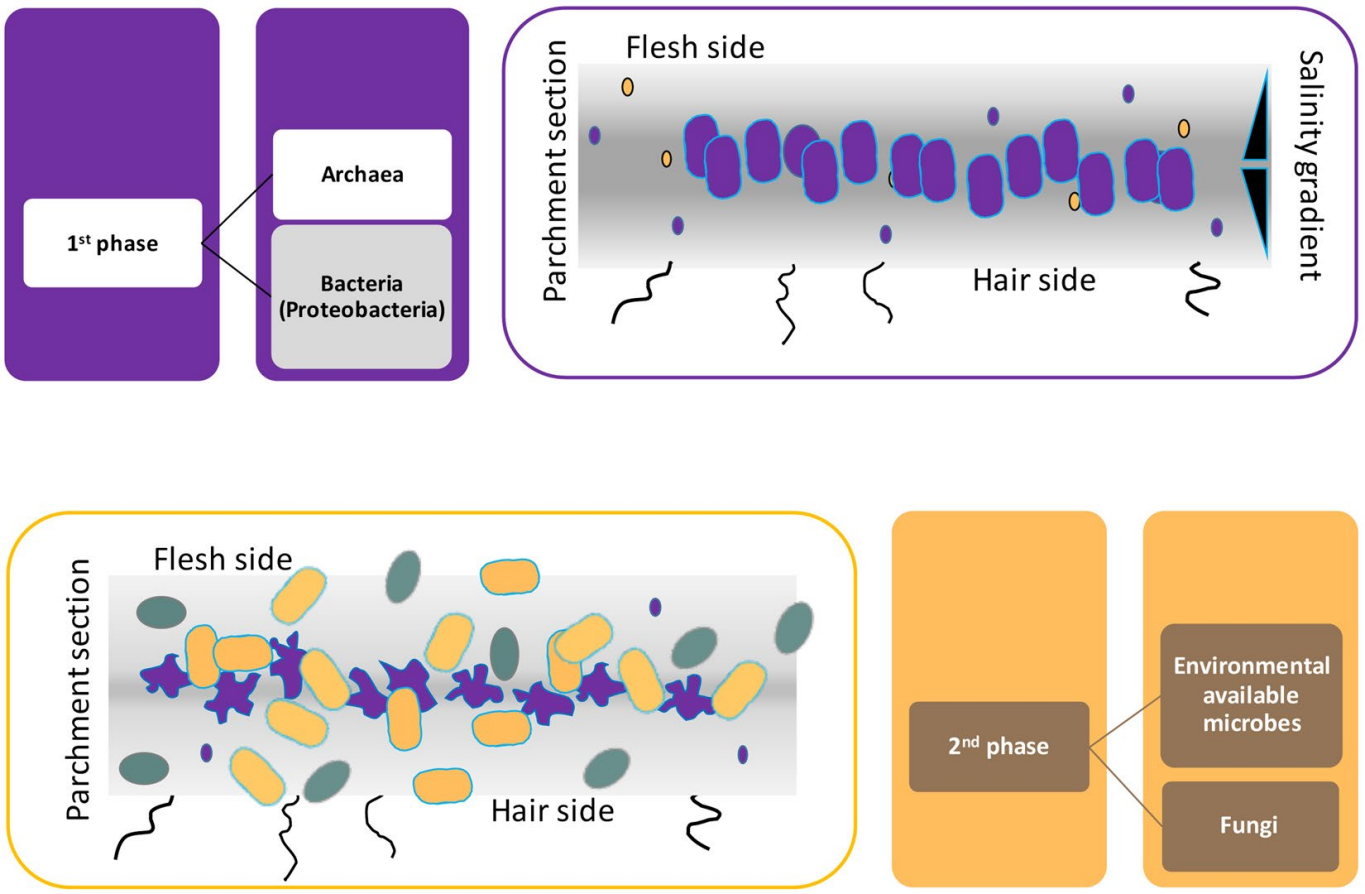
The two-phase model of parchment colonization. The microbes involved in the parchment damage are: halophilic Archaea (purple, the cells are intact in the 1^st^ phase draw or collapsed in the 2^nd^ one); Gamma-Proteobacteria (gold) and Fungi (teal).

Molecular methods reveal DNAs, which have not been removed in the production processes or later destroyed, but they do not obviously tell us the order of the events they witness. So we hypothesized a succession models, taking into account the possible provenience of the detected microorganisms, the selective forces allowing them to root, and the biological role they could have played. According to this information and to our results, the model of parchment deterioration is a microbial succession acting in two main phases (Fig. 7).

Abiotic and/or biotic agents can be considered as causative agent of the purple spot damage of the parchment, but the Raman analysis results point to the biotic ones. In this last case, a common initial colonization phase of the purple-damaged parchments can be hypothesized, having two possible scenarios, which could have been intertwined, occurring at different times or, even simultaneously, in different areas in the same parchment.

In fact, the microbial rhodopsins detected by the Raman spectrum, could originate from halophilic Archaea (Halobacteria) or from halotolerant Bacteria (probably Proteobacteria). A simpler hypothesis relies on proteorhodopsin-producing Bacteria, slowly growing in the nutrient poor parchment, staining and degrading it in several waves. Moisture, light, low temperatures and scarce availability of nutrients, all of these inducing the proteorhodopsin production could have been the triggering factors. However, the salty environment of the inner part of the dry parchment should have rather fostered the Halobacteria, dominant colonizers of the evaporation ponds and surely present in the marine salt used for the brining. With this in mind, Halobacteria should have been firstly acting as the main character in a more complex, two steps scheme, similarly to what happens nowadays in the red heat deterioration of brine-cured hides^33^. In ancient times, when parchment started to be exposed to humidity and light, only Halobacteria were allowed to grow in a sub-superficial layer of the parchment at the flesh side, where environmental conditions are permissive to them: Halobacteria need at least 5–10% NaCl for their very existence, take the dominance in low nutrient contexts, and like warm temperatures^34^. When the parchment moistened due to local events, probably in the warm season, the Halobacteria, associated to salt particles inside the parchment, began to grow in the still intact structure, starting to deteriorate the collagen matrix. The bacteriorhodopsin producing colonies formed the core of the purple spots and haloarchaeal collagenases enriched the available nutrients in the early spots. Although the 515F/806R primers have been proven suitable for the amplification of Halobacteria, most of which produce bacteriorhodopsin, no related amplicons have been observed in this as in other works^2^; however, Halobacteria lyse whenever the salt concentration lowers below the limiting value, because the envelope glycoproteins need a high concentration of NaCl for their structural stability. Hence, the released archaeal cellular content could have provided further nutrients to the fast-growing Gammaproteobacteria which, besides going on in consuming the collagen matrix, wiped away the halobacterial debris, only leaving the purple stain. This could explain why the NSG approach failed to detect Halobacteria. In a nutrient-rich environment, the fast growing marine Gammaproteobacteria, which are adapted to a salt concentration of about 3%, expand, and outcome Halobacteria, especially at low temperatures^34^. This should have been occurring in the cold season and whenever the water content inside the parchment exceeded 15%^35^. The microbial growth in neighboring areas, at the same sub-superficial level, can be responsible of the high fragility of the surface layer of the parchment and the loss of the written areas on the flesh side (Fig. 2c).

In the second phase, the colonizers identity links to the individual history of each parchment. As time passed by, other environmental factors had been intervening: dust settled on the parchment surface, bringing many new bacterial intruders, so as did the repeated human handling.

Regarding the *A.A. Arm. I-XVIII 3328*, the second phase dominant OTUs are Actinobacteria, prevalently unclassified Pseudonocardiales or *Saccharopolyspora*; on other documents^2^, the dominant OTUs were Actinobacteria or Firmicutes, alone, together or mixed with Gamma-Proteobacteria, according to the document. Different second phase colonizers were found in the history of the Archimedes’ palimpsest:^36^ Gammaproteobacteria dominated and some accident involving a eutrophic freshwater environment seems to have been occurred.

Still, the parchment itself keeps on in exerting a selective pressure, so that, for example, Actinobacteria are always mainly represented by Pseudonocardiales, whilst Streptomycetes, among the most common soil microorganisms, are present neither in our parchment nor in the other two described in the literature. Among the man derived microorganism, many are typically associated to the harsh skin environment and able to stand low moisture and/or fairly high salt concentrations (e.g. micrococci, staphylococci, *Acinetobacter*).

Of course, even in the same document, the already damaged areas offer different environments: the NGS results, indeed, show that the distribution within Pseudonocardiales is not even. OTU #1 is more than 80 folds more frequent in the damaged areas, whilst OTU #2 about tenfold in the undamaged ones. Interestingly, the best homologues to OTU #1 are some out of the uncultured *Saccharopolyspora*-like sequences found by Pinar^2^ as shown in supporting material (Table S2); so, a particular group of Pseudonocardiales could actually find a suitable niche in the modified environment of the purple damaged areas. The best scoring homologue to OTU #2 is the *Saccharopolyspora* sp. AFM 10238 (98%, KF673492) firstly isolated from the Dead Sea.

As final considerations, at the end of the colonization probably both the complexity of biological attack and the later drying of the parchment cooperated to the cracking, detachment and loss of superficial pieces at the flesh side of the parchment, including the written areas. Furthermore, in the second phase, often fungi worsened the damage; fungi are also linked to the individual history of the parchment, and it should be interesting to investigate how they were selected by the changing environments of the damaged/undamaged areas of the parchment.

In conclusion, the integrated approach including physical, chemical and microbiological tools, has been able to give a detailed picture of the processes and actors of the bacterial alterations found on the ancient parchment. The NGS is the most affordable tool to appreciate also the fine-tuned differences in the quality and quantity of the past and present microbial colonizers. The Raman analysis offers a precious clue for the actual identity of the microbial pigments in the purple spots, recalling - on a scientific basis - the analogy with the red heats affecting today’s hides. The new Light Transmitted Analysis technique allowed to assess the amount of damages affecting the structure of the collagen matrix.

The new information on the colonization and deterioration processes, including the kind of damage to the collagen fibers and the chemical composition of the purple spots, open new perspectives to the restoration and conservation of ancient damaged parchments. The empirical procedures of conservation currently in use (controlled atmosphere and moisture, protection from light) are completely sound to avoid new damages to the ancient documents, but further studies could help to understand which kind of rhodopsins are involved in the purple spots to look for possible new restoration approaches for damaged documents. Moreover, a better understanding of the possible role of Halobacteria could be useful, as they can survive for huge times^37^, being a possible “time bomb” in the ancient undamaged parchments.

## Materials and Methods

### Microbial analysis

#### Sample collection

For the microbial community characterization, small pieces of parchment of about 2–4 mm^2^, already detached and impossible to be relocated during the restoration process, were collected from purple stained and white areas in sterile conditions. Each replicate was composed of one piece of parchment: 4 replicates of purple pieces and 3 of uncoloured (white) pieces as a control were utilized. The 3 white pieces were chosen in two different areas of the parchment: two belong to a well preserved and undamaged area far from the purple spots, while the third has been collected in an uncoloured area in between a purple spotted area of the parchment.

#### Bacterial pellet and DNA extraction

To extract metagenomic DNA, each parchment piece was directly processed using the Power Soil®DNA isolation kit (Mo Bio, Carlsbad, CA, USA) according to the manufacturer’s instructions.

#### 454 pyrosequencing

Pure DNA extracts were sent to the Molecular Research LP in TX, USA (MR DNA, http://www.mrdnalab.com/ ref. 38,) for PCR amplification of the 16 S rRNA gene of Bacteria and followed by 454 pyrosequencing. The primers 515F (Forward, 5-GTGCCAGCMGCCGCGGTAA-3) and 806R (Reverse, 5-GGACTACHVGGGTWTCTAAT-3), were used to amplify the phylogenetically highly variable regions, V4^39^. Amplification of the metagenomic DNA was obtained from a single-step 30 cycle PCR using HotStarTaq Plus Master Mix Kit (Qiagen, Valencia, CA) with an initial denaturation step of 94 °C for 3 min, followed by 28 cycles of 94 °C for 30 s; 53 °C for 40 s and 72 °C for 1 min, with a final extension step of 72 °C for 5 min. Amplicon products were mixed in equal concentrations and purified using Agencourt Ampure beads (Agencourt Bioscience Corporation, MA, USA). Samples were then sequenced utilizing Roche 454 FLX titanium instruments and reagents following the manufacturer’s guidelines.

#### 454 pyrosequencing data processing

The 454-pyrosequencing raw data were processed with the open source software MOTHUR (http://www.mothur.org/), according to the 454 Standard Operating Procedure pipeline^40^ available online at the software’s website. Briefly, in the pipeline, quality trimming was performed, sequences were depleted of their barcodes and those <200 bp and with ambiguous base calls were removed. These sequences were then dereplicated, aligned against the Greengenes coreset template alignment, and screened to make them overlap in the same region. Putative chimeras were identified with the “uchime” algorithm and removed. Operational Taxonomic Units (OTUs) were defined by clustering of sequences at 3% divergence (97% similarity). A final matrix of the OTUs assigned to each sample was built in MOTHUR and used in downstream analyses. The complete set of raw sequences obtained in this study has been deposited in GenBank at the Sequence Read Archive (SRA) under the study accession no. SUB2026926; BioProject no. PRJNA34922.

High quality sequences were assigned a taxonomical identity using the “Classifier” program^41^ at the RDP site (Release 11, May 26, 2015) and applying the 50% confidence threshold suggested for the sequences shorter than 250 bp^42^. The taxonomical assignments for Actinobacteria were adjusted to match the recent taxonomy changes in this field^43, 44^.

To reveal the taxonomic diversity of the OTUs, the sequences were aligned with the Mafft v7.304b program (http://mafft.cbrc.jp/alignment/server/) with the “auto” option, and a rough tree was constructed by the UPGMA method. Tree figure was generated using the interactive Tree of Life web application (itol.embl.de)^45^.

The OTUs sequences were compared with the online NCBI database (blast.ncbi.nlm.nih.gov/Blast.cgi), using the nucleotide BLAST search program^46^.

#### Bacterial community analyses

Microbial community structure and composition were analysed in MOTHUR as follows: (i) rarefaction curves were built to evaluate differences in sampling effort (Fig. 3A); (ii) the Shannon index (H’) was used to evaluate bacterial diversity; (iii) wireframe charts were built to visualize the bacterial community composition and their relative abundances. Multivariate analyses of OTU data were also performed, unconstrained UPGMA clustering was built using the UniFrac.weighted metric within MOTHUR. To standardize differences in sampling effort among samples, the complete OTUs dataset was normalized by random subsampling to a common depth (the lowest number of sequences produced by the samples, 588). The latter allows for adequate comparisons at community level (Shannon diversity index, n-MDS analysis and UniFrac.weighted measurements).

#### Statistical analyses

n-MDS ordination was conducted on a Bray–Curtis distance matrix calculated between sampling plots with log(x + 1)-transformed OTUs abundance data. ANOSIM (n = 999 randomizations^47^;) was employed to test for significant differences in microbial communities between the two kinds of samples. The UniFrac.weighted algorithm was used in MOTHUR to evaluate if the microbial community structure of the two types of samples differed significantly based on their phylogenetic relationships and the abundances of the taxonomic groups present in the samples, and statistical significance was defined at p < 0.01. The UniFrac.weighted analysis, which measures the difference between collections of sequences as the amount of evolutionary history that is unique to each one^48, 49^ evaluated differences in the bacterial community structure, based on the OTUs phylogenetic relationships and abundance.

### Chemical analyses

#### Raman analysis

The Raman spectra was performed firstly on parchment samples; in the parchment samples fluorescence was the dominant response and moreover, the red spots were difficult to be focalized due to heterogeneity of the support and their not properly identifiable positioning on the parchment surface. Results of this analysis were thus unusable and are not reported here. Raman analyses were performed by using a micro-Raman spectrometer eXplora system (Horiba) with a laser source at 785 nm and power less than 1 mW, 100x magnification and 3 cm^−1^ spectral resolution. Samples were taken in collaboration with restorers by scraping of few mg of red spotted parchment materials. Raman analysis were performed both directly on samples and on chemically extracted pigments. The solvent extraction was performed using 2 ml of dichloromethane, few microliter of extracted solution was then evaporated on Si substrate and directly used for micro Raman measurements.

### Physical analyses

#### Light Transmitted Analysis (LTA) with visual control by microscopy

The denaturation process of the collagen populations in the parchment has been investigated by LTA associated to visual control by microscopy. The samples were prepared as follows: a small amount of parchment was immersed in distilled water, defibrated by using a scalpel and partially drained to obtain a compact fibre pulp. Single samples containing about 0.5 mg of parchment fibre were rehydrated in excess water to get a fibre density of about 0.3 g/cm^3^ and sealed inside a quartz cell of calibrated thickness (100 μm). The cell was placed into an oven equipped with a remote temperature control system and two windows for the optical access. The sample layer was lighted from the bottom window by an expanded and collimated He-Ne laser beam (λ = 633 nm) at modulated intensity. The emerging light, passed through the full thickness of the sample, is collected at the top window by a lens (4 cm focal length) and focused onto a photodiode. This process generates a periodic signal, analysed by a lock-in amplifier. The signal amplitude was recorded for the whole process of hydrothermal denaturation, every 0.05 °C during the heating scans, running from 25 to 90 °C. During the heating scan, the sample was inspected through the top window by a real time polarized microscopy system, equipped with a CCD (Charge-Coupled Device) camera. High quality images were obtained thanks to the partially transparent titanium coating of the cell bottom, which allows both the light reflection from the imaging system and the transmission of the laser beam.

#### Microstructure morphology. SEM

Images of the parchment microstructure the have been taken by scanning electron microscopy (SEM) both for well preserved and highly deteriorated samples. The employed microscope is a Hitachi S-2460N model, resolution 4 nm, magnification 20–200.000x, accelerating voltage 0.3 to 30 kV.

## Electronic supplementary material

Supplementary Information Tab. S1, S2 and Fig. S1
