# Supplementary material for: Purple spot damage dynamics investigated by an integrated approach on a 1244 A.D. parchment roll from the Secret Vatican Archive
Source: Sci Rep. 2017 Sep 7;7:9521. doi: 10.1038/s41598-017-05398-7 (PMC5589745; doi:10.1038/s41598-017-05398-7)
Supplement: Supplementary file 1 — Supplementary Information Tab. S1, S2 and Fig. S1 [file 41598_2017_5398_MOESM1_ESM.pdf]

Purple spot damage dynamics investigated by an integrated approach on a 1244 A.D. parchment roll from the Secret Vatican Archive – Migliore L., Thaller M.C., Vendittozzi G., Mejia A.Y., Mercuri F., Orlanducci S. & Rubechini A.

Table S1. OTUs identification

OTU n°	Dominion (% score)	Phylum (% score)	Class (% score)	Order (% score)	Family (% score)	Genus (% score)
OTU0001	Bacteria (100)	Actinobacteria (100)	Actinobacteria (100)	Pseudonocardiales (100)	Pseudonocardiaceae (91)	unclassified (<50)
OTU0002	Bacteria (100)	Actinobacteria (100)	Actinobacteria (100)	Pseudonocardiales (100)	Pseudonocardiaceae (79)	unclassified (<50)
OTU0003	Bacteria (100)	Proteobacteria (100)	Gammaproteobacteria (100)	Vibrionales (100)	Vibrionaceae (100)	*Vibrio* (79)
OTU0004	Bacteria (100)	Actinobacteria (100)	Actinobacteria (100)	Pseudonocardiales (100)	Pseudonocardiaceae (99)	*Saccharopolyspora* (50)
OTU0005	Bacteria (100)	Proteobacteria (100)	Gammaproteobacteria (100)	Alteromonadales (100)	Pseudoalteromonadaceae (100)	*Pseudoalteromonas* (100)
OTU0006	Bacteria (100)	Proteobacteria (100)	Gammaproteobacteria (100)	Vibrionales (100)	Vibrionaceae (100)	*Vibrio* (63)
OTU0007	Bacteria (100)	Proteobacteria (100)	Gammaproteobacteria (100)	Pseudomonadales (100)	Moraxellaceae (100)	*Acinetobacter* (100)
OTU0008	Bacteria (100)	Proteobacteria (100)	Gammaproteobacteria (100)	Aeromonadales (98)	Aeromonadaceae (98)	*Aeromonas* (98)
OTU0009	Bacteria (100)	Proteobacteria (100)	Gammaproteobacteria (100)	Vibrionales (100)	Vibrionaceae (100)	*Vibrio* (95)
OTU0010	Bacteria (100)	Proteobacteria (100)	Gammaproteobacteria (100)	Vibrionales (94)	Vibrionaceae (94)	*Photobacterium* (56)
OTU0011	Bacteria (100)	Proteobacteria (100)	Gammaproteobacteria (100)	Pseudomonadales (100)	Moraxellaceae (100)	*Acinetobacter* (100)
OTU0012	Bacteria (100)	Firmicutes (100)	Bacilli (100)	Bacillalles (100)	Staphylococcaceae (100)	*Staphylococcus* (100)
OTU0013	Bacteria (100)	Proteobacteria (100)	Gammaproteobacteria (100)	Pseudomonadales (100)	Pseudomonadaceae (100)	*Pseudomonas* (100)
OTU0014	Bacteria (100)	Firmicutes (100)	Bacilli (100)	Lactobacillales (100)	Streptococcaceae (100)	*Streptococcus* (100)
OTU0015	Bacteria (100)	Actinobacteria (100)	Actinobacteria (100)	Pseudonocardiales (100)	Pseudonocardiaceae (82)	unclassified (<50)
OTU0016	Bacteria (100)	Proteobacteria (100)	Gammaproteobacteria (100)	Enterobacteriales (100)	Enterobacteriaceae (100)	unclassified (<50)
OTU0017	Bacteria (100)	Proteobacteria (100)	Alphaproteobacteria (100)	Rhizobiales (97)	Phyllobacteriaceae (94)	*Phyllobacterium* (94)
OTU0018	Bacteria (100)	Firmicutes (100)	Bacilli (100)	Lactobacillales (99)	Enterococcaceae (98)	*Enterococcus* (98)
OTU0019	Bacteria (100)	Actinobacteria (100)	Actinobacteria (100)	Pseudonocardiales (100)	Pseudonocardiaceae (97)	*Saccharopolyspora* (74)

OTU0020	Bacteria (100)	Actinobacteria (100)	Actinobacteria (100)	Corynebacteriales (100)	Corynebacteriaceae (100)	*Corynebacterium* (100)
OTU0021	Bacteria (100)	Firmicutes (100)	Bacilli (100)	Bacillalles (100)	Staphylococcaceae (100)	*Staphylococcus* (96)
OTU0022	Bacteria (100)	Proteobacteria (100)	Gammaproteobacteria (100)	Vibrionales (100)	Vibrionaceae (100)	*Vibrio* (83)
OTU0023	Bacteria (100)	Firmicutes (100)	Bacilli (100)	Bacillales (94)	Bacillaceae1 (67)	*Bacillus* (65)
OTU0024	Bacteria (100)	Proteobacteria (100)	Gammaproteobacteria (100)	Enterobacteriales (100)	Enterobacteriaceae (100)	*Escherichia/Shigella* (84)
OTU0025	Bacteria (100)	Proteobacteria (100)	Gammaproteobacteria (100)	Vibrionales (100)	Vibrionaceae (100)	*Vibrio* (72)
OTU0026	Bacteria (100)	Actinobacteria (100)	Actinobacteria (100)	Pseudonocardiales (100)	Pseudonocardiaceae (84)	unclassified (<50)
OTU0027	Bacteria (100)	Proteobacteria (100)	Betaproteobacteria (100)	Neisseriales (96)	Neisseriaceae (96)	unclassified (<50)
OTU0028	Bacteria (100)	Actinobacteria (100)	Actinobacteria (100)	Pseudonocardiales (100)	Pseudonocardiaceae (98)	*Saccharopolyspora* (84)
OTU0029	Bacteria (100)	Actinobacteria (98)	Actinobacteria (98)	Corynebacteriales (98)	Dietziaceae (55)	*Dietzia* (55)
OTU0030	Bacteria (100)	Proteobacteria (100)	Gammaproteobacteria (100)	Vibrionales (100)	Vibrionaceae (100)	*Vibrio* (95)
OTU0031	Bacteria (100)	Actinobacteria (100)	Actinobacteria (100)	Pseudonocardiales (100)	Pseudonocardiaceae (95)	unclassified (<50)
OTU0032	Bacteria (100)	Proteobacteria (100)	Gammaproteobacteria (100)	Pseudomonadales (100)	Moraxellaceae (100)	*Acinetobacter* (100)
OTU0033	Bacteria (100)	Actinobacteria (100)	Actinobacteria (100)	Corynebacteriales (100)	Corynebacteriaceae (100)	*Turicella* (97)
OTU0034	Bacteria (100)	Proteobacteria (100)	Gammaproteobacteria (100)	Pseudomonadales (100)	Pseudomonadaceae (100)	*Pseudomonas* (86)
OTU0035	Bacteria (100)	Proteobacteria (100)	Gammaproteobacteria (100)	Aeromonadales (100)	Aeromonadaceae (100)	*Alteromonas* (95)
OTU0036	Bacteria (100)	Proteobacteria (100)	Gammaproteobacteria (100)	Pseudomonadales (100)	Moraxellaceae (100)	*Acinetobacter* (99)
OTU0037	Bacteria (100)	Proteobacteria (100)	Gammaproteobacteria (100)	Pseudomonadales (99)	Pseudomonadaceae (99)	*Pseudomonas* (94)
OTU0039	Bacteria (100)	Proteobacteria (100)	Gammaproteobacteria (100)	Vibrionales (100)	Vibrionaceae (100)	*Photobacterium* (55)
OTU0040	Bacteria (100)	Proteobacteria (100)	Betaproteobacteria (100)	Burkholderiales (100)	Oxalobacteraceae (100)	*Massilia* (91)
OTU0041	Bacteria (100)	Firmicutes (100)	Bacilli (100)	Bacillales (96)	Staphylococcaceae (94)	*Staphylococcus* (91)
OTU0042	Bacteria (100)	Firmicutes (100)	Bacilli (100)	Lactobacillales (100)	Streptococcaceae (100)	*Streptococcus* (100)

**OTU0043**	**Bacteria (100)**	**Proteobacteria (100)**	**Gammaproteobacteria (100)**	**Oceanospirillales (100)**	**Oceanospirillaceae (100)**	***Marinomonas* (100)**
**OTU0044**	**Bacteria (100)**	**Firmicutes (100)**	**Bacilli (100)**	**Lactobacillales (100)**	**Streptococcaceae (100)**	***Streptococcus* (100)**
**OTU0045**	**Bacteria (100)**	**Firmicutes (100)**	**Clostridia (100)**	**Clostridiales (100)**	**Clostridiales_Incertae Sedis XI (100)**	***Finegoldia* (100)**
**OTU0046**	**Bacteria (100)**	**Actinobacteria (100)**	**Actinobacteria (100)**	**Micrococcales (100)**	**Micrococcaceae (100)**	***Micrococcus* (100)**
**OTU0047**	**Bacteria (100)**	**Actinobacteria (100)**	**Actinobacteria (100)**	**Pseudonocardiales (100)**	**Pseudonocardiaceae (98)**	***Saccharopolyspora* (52)**
**OTU0048**	**Bacteria (100)**	**Proteobacteria (100)**	**Gammaproteobacteria (100)**	**Pseudomonadales (100)**	**Moraxellaceae (100)**	***Enhydrobacter* (100)**
**OTU0049**	**Bacteria (100)**	**Actinobacteria (100)**	**Actinobacteria (100)**	**Corynebacteriales (100)**	**Corynebacteriaceae (100)**	***Corynebacterium* (100)**
**OTU0050**	**Bacteria (100)**	**Actinobacteria (100)**	**Actinobacteria (100)**	**Corynebacteriales (100)**	**Corynebacteriaceae (100)**	***Corynebacterium* (100)**
**OTU0051**	**Bacteria (100)**	**Actinobacteria (100)**	**Actinobacteria (100)**	**Pseudonocardiales (100)**	**Pseudonocardiaceae (89)**	***Saccharopolyspora* (82)**
**OTU0052**	**Bacteria (100)**	**Proteobacteria (100)**	**Gammaproteobacteria (100)**	**Alteromonadales (100)**	**Pseudoalteromonadaceae (100)**	***Pseudoalteromonas* (100)**
**OTU0053**	**Bacteria (100)**	**Proteobacteria (100)**	**Gammaproteobacteria (100)**	**Vibrionales (90)**	**Vibrionaceae (90)**	**unclassified (<50)**
**OTU0054**	**Bacteria (100)**	**Actinobacteria (100)**	**Actinobacteria (100)**	**Pseudonocardiales (99)**	**Pseudonocardiaceae (94)**	**unclassified (<50)**
**OTU0055**	**Bacteria (100)**	**Proteobacteria (100)**	**Gammaproteobacteria (100)**	**Pseudomonadales (100)**	**Moraxellaceae (100)**	***Acinetobacter* (100)**
**OTU0056**	**Bacteria (100)**	**Proteobacteria (100)**	**Gammaproteobacteria (100)**	**Vibrionales (100)**	**Vibrionaceae (100)**	***Vibrio* (94)**
**OTU0057**	**Bacteria (100)**	**Proteobacteria (100)**	**Gammaproteobacteria (100)**	**Pseudomonadales (100)**	**Moraxellaceae (100)**	***Acinetobacter* (100)**
**OTU0058**	**Bacteria (100)**	**Proteobacteria (100)**	**Betaproteobacteria (100)**	**Burkholderiales (100)**	**Oxalobacteraceae (100)**	***Massilia* (89)**
**OTU0059**	**Bacteria (100)**	**Proteobacteria (100)**	**Gammaproteobacteria (100)**	**Pseudomonadales (100)**	**Moraxellaceae (100)**	***Acinetobacter* (100)**
**OTU0060**	**Bacteria (100)**	**Proteobacteria (100)**	**Gammaproteobacteria (100)**	**Pasteurellales (100)**	**Pasteurellaceae (100)**	***Haemophilus* (66)**
**OTU0061**	**Bacteria (100)**	**Proteobacteria (100)**	**Gammaproteobacteria (100)**	**Oceanospirillales (100)**	**Halomonadaceae (100)**	***Halomonas* (97)**
**OTU0062**	**Bacteria (100)**	**Proteobacteria (100)**	**Gammaproteobacteria (100)**	**Vibrionales (100)**	**Vibrionaceae (100)**	***Allomonas* (53)**
**OTU0063**	**Bacteria (100)**	**Firmicutes (100)**	**Bacilli (100)**	**Lactobacillales (100)**	**Carnobacteriaceae (100)**	***Dolosigranulum* (100)**
**OTU0064**	**Bacteria (100)**	**Proteobacteria (100)**	**Gammaproteobacteria (100)**	**Vibrionales (96)**	**Vibrionaceae (96)**	***Photobacterium* (55)**
**OTU0065**	**Bacteria (100)**	**Firmicutes (100)**	**Bacilli (100)**	**Lactobacillales (100)**	**Streptococcaceae (100)**	***Streptococcus* (99)**

**OTU0066**	**Bacteria (100)**	**Proteobacteria (100)**	**Gammaproteobacteria (100)**	**Pseudomonadales (100)**	**Moraxellaceae (100)**	***Acinetobacter* (100)**
**OTU0067**	**Bacteria (100)**	**Proteobacteria (100)**	**Alphaproteobacteria (100)**	**Rhodobacterales (100)**	**Rhodobacteraceae (100)**	***Paracoccus***
**OTU0068**	**Bacteria (100)**	**Proteobacteria (100)**	**Gammaproteobacteria (100)**	**Vibrionales (100)**	**Vibrionaceae (100)**	***Allomonas* (50)**
**OTU0069**	**Bacteria (100)**	**Proteobacteria (100)**	**Alphaproteobacteria (100)**	**Rhodobacterales (98)**	**Rhodobacteraceae (98)**	**unclassified (<50)**
**OTU0070**	**Bacteria (100)**	**Firmicutes (100)**	**Bacilli (100)**	**Bacillalles (100)**	**Bacillales_Incertae Sedis XII (100)**	***Gemella* (100)**
**OTU0071**	**Bacteria (100)**	**Proteobacteria (100)**	**Gammaproteobacteria (100)**	**Oceanospirillales (100)**	**Halomonadaceae (98)**	**unclassified (<50)**
**OTU0072**	**Bacteria (100)**	**Firmicutes (100)**	**Bacilli (100)**	**Lactobacillales (100)**	**unclassified (<50)**	**unclassified (<50)**
**OTU0073**	**Bacteria (100)**	**Fusobacteria (100)**	**Fusobacteriia (100)**	**Fusobacteriales (100)**	**Fusobacteriaceae (100)**	**unclassified (<50)**
**OTU0074**	**Bacteria (100)**	**Proteobacteria (100)**	**Gammaproteobacteria (100)**	**Vibrionales (99)**	**Vibrionaceae (99)**	**unclassified (<50)**
**OTU0075**	**Bacteria (100)**	**Proteobacteria (100)**	**Gammaproteobacteria (100)**	**Alteromonadales (99)**	**Shewanellaceae (98)**	***Shewanella* (98)**
**OTU0076**	**Bacteria (100)**	**Firmicutes (100)**	**Negativicutes (100)**	**Selenomonadales (100)**	**Veillonellaceae (100)**	***Veillonella* (84)**
**OTU0077**	**Bacteria (100)**	**Actinobacteria (98)**	**Actinobacteria (98)**	**Pseudonocardiales (98)**	**Pseudonocardiaceae (78)**	**unclassified (<50)**
**OTU0078**	**Bacteria (100)**	**Proteobacteria (100)**	**Gammaproteobacteria (100)**	**Vibrionales (100)**	**Vibrionaceae (100)**	***Aliivibrio* (100)**
**OTU0079**	**Bacteria (100)**	**Actinobacteria (100)**	**Actinobacteria (100)**	**Pseudonocardiales (98)**	**Pseudonocardiaceae (77)**	**unclassified (<50)**
**OTU0080**	**Bacteria (100)**	**Actinobacteria (100)**	**Actinobacteria (100)**	**Corynebacteriales (100)**	**Corynebacteriaceae (84)**	***Corynebacterium* (84)**
**OTU0081**	**Bacteria (100)**	**Firmicutes (100)**	**Clostridia (100)**	**Clostridiales (100)**	**Clostridiales_Incertae Sedis XI (100)**	***Anaerococcus* (100)**
**OTU0082**	**Bacteria (100)**	**Fusobacteria (100)**	**Fusobacteriia (100)**	**Fusobacteriales (100)**	**Fusobacteriaceae (100)**	***Fusobacterium* (98)**
**OTU0083**	**Bacteria (100)**	**Proteobacteria (100)**	**Gammaproteobacteria (100)**	**Vibrionales (100)**	**Vibrionaceae (100)**	***Allomonas* (56)**
**OTU0084**	**Bacteria (100)**	**Actinobacteria (100)**	**Actinobacteria (100)**	**Corynebacteriales (100)**	**Corynebacteriaceae (98)**	***Corynebacterium* (98)**
**OTU0085**	**Bacteria (100)**	**Actinobacteria (100)**	**Actinobacteria (100)**	**Actinomycetales (100)**	**Actinomycetaceae (100)**	***Actinomyces* (100)**
**OTU0086**	**Bacteria (100)**	**Proteobacteria (100)**	**Gammaproteobacteria (100)**	**Vibrionales (100)**	**Vibrionaceae (100)**	***Vibrio* (91)**
**OTU0087**	**Bacteria (100)**	**Proteobacteria (100)**	**Gammaproteobacteria (100)**	**Pasteurellales (100)**	**Pasteurellaceae (100)**	***Haemophilus* (91)**
**OTU0088**	**Bacteria (100)**	**Actinobacteria (100)**	**Actinobacteria (100)**	**Micrococcales (100)**	**Micrococcaceae (100)**	***Rothia* (100)**
**OTU0089**	**Bacteria (100)**	**Proteobacteria (100)**	**Gammaproteobacteria (100)**	**Pseudomonadales (98)**	**Pseudomonadaceae (98)**	***Pseudomonas* (87)**

**OUT0090**	**Bacteria (100)**	**Proteobacteria (100)**	**Gammaproteobacteria (100)**	**Vibrionales (94)**	**Vibrionaceae (94)**	***Vibrio* (67)**
**OUT0091**	**Bacteria (100)**	**Firmicutes (99)**	**Bacilli (99)**	**Lactobacillales (99)**	**Lactobacillaceae (87)**	**unclassified (<50)**
**OUT0092**	**Bacteria (100)**	**Actinobacteria (100)**	**Actinobacteria (100)**	**Corynebacteriales (100)**	**Corynebacteriaceae (100)**	***Corynebacterium* (100)**
**OUT0093**	**Bacteria (100)**	**Proteobacteria (100)**	**Gammaproteobacteria (100)**	**Aeromonadales (100)**	**Aeromonadaceae (100)**	***Aeromonas* (100)**
**OUT0094**	**Bacteria (100)**	**Actinobacteria (100)**	**Actinobacteria (100)**	**Micrococcales (100)**	**Micrococcaceae (99)**	***Kocuria* (95)**
**OUT0095**	**Bacteria (100)**	**Proteobacteria (100)**	**Gammaproteobacteria (100)**	**Pasteurellales (100)**	**Pasteurellaceae (100)**	***Haemophilus* (76)**
**OUT0096**	**Bacteria (100)**	**Actinobacteria (100)**	**Actinobacteria (100)**	**Pseudonocardiales (100)**	**Pseudonocardiaceae (73)**	**unclassified (<50)**
**OUT0097**	**Bacteria (100)**	**Proteobacteria (100)**	**Gammaproteobacteria (100)**	**Pseudomonadales (100)**	**Pseudomonadaceae (100)**	***Pseudomonas* (79)**
**OUT0098**	**Bacteria (100)**	**Proteobacteria (100)**	**Gammaproteobacteria (100)**	**Vibrionales (100)**	**Vibrionaceae (100)**	**unclassified (<50)**
**OUT0099**	**Bacteria (100)**	**Proteobacteria (100)**	**Gammaproteobacteria (100)**	**Vibrionales (100)**	**Vibrionaceae (100)**	***Allomonas* (70)**
**OUT0100**	**Bacteria (100)**	**Proteobacteria (100)**	**Alphaproteobacteria (100)**	**Rhodospirillales (100)**	**Rhodospirillaceae (100)**	***Azospirillum* (99)**
**OUT0101**	**Bacteria (100)**	**Firmicutes (100)**	**Negativicutes (100)**	**Selenomonadales (100)**	**Veillonellaceae (100)**	***Veillonella* (100)**
**OUT0102**	**Bacteria (100)**	**Proteobacteria (100)**	**Gammaproteobacteria (100)**	**Vibrionales (100)**	**Vibrionaceae (100)**	***Vibrio* (61)**
**OUT0103**	**Bacteria (100)**	**Proteobacteria (100)**	**Gammaproteobacteria (100)**	**Chromatiales (100)**	**Chromatiaceae (100)**	***Rheinheimera* (100)**
**OUT0104**	**Bacteria (100)**	**Fusobacteria (100)**	**Fusobacteriia (100)**	**Fusobacteriales (100)**	**Fusobacteriaceae (100)**	***Cetobacterium* (92)**
**OUT0105**	**Bacteria (100)**	**Actinobacteria (100)**	**Actinobacteria (100)**	**Pseudonocardiales (100)**	**Pseudonocardiaceae (97)**	**unclassified (<50)**
**OUT0106**	**Bacteria (100)**	**Actinobacteria (100)**	**Actinobacteria (100)**	**Pseudonocardiales (100)**	**Pseudonocardiaceae (99)**	**unclassified (<50)**
**OUT0107**	**Bacteria (100)**	**Actinobacteria (100)**	**Actinobacteria (100)**	**Corynebacteriales (100)**	**Corynebacteriaceae (100)**	***Corynebacterium* (100)**
**OUT0108**	**Bacteria (100)**	**Actinobacteria (100)**	**Actinobacteria (100)**	**Micrococcales (100)**	**Micrococcaceae (100)**	***Rothia* (100)**
**OUT0109**	**Bacteria (100)**	**Proteobacteria (100)**	**Gammaproteobacteria (100)**	**Vibrionales (100)**	**Vibrionaceae (100)**	***Vibrio* (59)**
**OUT0110**	**Bacteria (100)**	**Proteobacteria (100)**	**Gammaproteobacteria (100)**	**Aeromonadales (99)**	**Aeromonadaceae (99)**	***Aeromonas* (96)**
**OUT0111**	**Bacteria (100)**	**Proteobacteria (100)**	**Betaproteobacteria (94**	**Neisseriales (70)**	**Neisseriaceae (70)**	**unclassified (<50)**
**OUT0112**	**Bacteria (100)**	**Proteobacteria (100)**	**Gammaproteobacteria (100)**	**Pseudomonadales (96)**	**Pseudomonadaceae (96)**	***Pseudomonas* (75)**
**OUT0113**	**Bacteria (100)**	**Actinobacteria (100)**	**Actinobacteria (100)**	**Pseudonocardiales (100)**	**Pseudonocardiaceae (89)**	**unclassified (<50)**

OTU0114	Bacteria (100)	Firmicutes (100)	Clostridia (100)	Clostridiales (100)	Clostridiales_Incertae Sedis XI (100)	*Peptoniphilus* (100)
OTU0115	Bacteria (100)	Bacteroidetes (98)	Bacteroidia (85	Bacteroidales (85)	Prevotellaceae (77)	*Alloprevotella* (66)
OTU0116	Bacteria (100)	Actinobacteria (100)	Actinobacteria (100)	Pseudonocardiales (98)	Pseudonocardiaceae (82)	unclassified (<50)
OUT0117	Bacteria (100)	Firmicutes (99)	Bacilli (99)	Bacillales (78)	Staphylococcaceae (71)	*Staphylococcus* (71)
OUT0118	Bacteria (100)	Actinobacteria (100)	Actinobacteria (100)	Corynebacteriales (100)	Corynebacteriaceae (91)	*Corynebacterium* (91)
OUT0119	Bacteria (100)	Proteobacteria (100)	Gammaproteobacteria (100)	Vibrionales (100)	Vibrionaceae (100)	*Allomonas* (53)
OUT0120	Bacteria (100)	Actinobacteria (99)	Actinobacteria (99)	Corynebacteriales (99)	unclassified (<50)	unclassified (<50)
OUT0121	Bacteria (100)	Proteobacteria (100)	Alphaproteobacteria (100)	Rhodobacterales (87)	Rhodobacteraceae (87)	unclassified (<50)
OUT0122	Bacteria (100)	Actinobacteria (100)	Actinobacteria (100)	Pseudonocardiales (98)	Pseudonocardiaceae (85)	unclassified (<50)
OUT0123	Bacteria (100)	Proteobacteria (100)	Gammaproteobacteria (100)	Vibrionales (100)	Vibrionaceae (100)	*Vibrio* (76)
OUT0124	Bacteria (100)	Proteobacteria (100)	Gammaproteobacteria (100)	Vibrionales (94)	Vibrionaceae (94)	*Vibrio* (67)
OUT0125	Bacteria (100)	Deinococcus-Thermus (100)	Deinococci (100)	Deinococcales (100)	Deinococcaceae (100)	*Deinococcus* (100)
OUT0126	Bacteria (100)	Proteobacteria (100)	Betaproteobacteria (100)	Neisseriales (95)	Neisseriaceae (95)	unclassified (<50)
OUT0127	Bacteria (100)	Actinobacteria (100)	Actinobacteria (100)	Pseudonocardiales (100)	Pseudonocardiaceae (89)	unclassified (<50)
OUT0130	Bacteria (100)	Proteobacteria (100)	Gammaproteobacteria (100)	Vibrionales (90)	Vibrionaceae (90)	*Salinivibrio* (56)
OUT0131	Bacteria (100)	unclassified (<50)	unclassified (<50)	unclassified (<50)	unclassified (<50)	unclassified (<50)
OUT0133	Bacteria (100)	Proteobacteria (100)	Gammaproteobacteria (100)	Enterobacteriales (100)	Enterobacteriaceae (100)	unclassified (<50)
OUT0134	Bacteria (100)	Fusobacteria (100)	Fusobacteriia (100)	Fusobacteriales (100)	Fusobacteriaceae (100)	*Cetobacterium* (97)
OUT0135	Bacteria (100)	Fusobacteria (100)	Fusobacteriia (100)	Fusobacteriales (100)	Fusobacteriaceae (100)	*Fusobacterium* (97)
OUT0136	Bacteria (100)	Proteobacteria (100)	Gammaproteobacteria (100)	Xanthomonadales (100)	Xanthomonadaceae (100)	*Stenotrophomonas* (97)
OUT0137	Bacteria (100)	Firmicutes (100)	Bacilli (100)	Bacillalles (100)	Staphylococcaceae (99)	*Salinicoccus* (81)
OUT0138	Bacteria (100)	Proteobacteria (100)	Alphaproteobacteria (100)	Rhodobacterales (89)	Rhodobacteraceae (89)	unclassified (<50)

OUT0139	Bacteria (100)	Actinobacteria (100)	Actinobacteria (100)	Pseudonocardiales (100)	unclassified (<50)	unclassified (<50)
OUT0140	Bacteria (100)	Proteobacteria (100)	Gammaproteobacteria (100)	Pseudomonadales (79)	Moraxellaceae (79)	*Acinetobacter* (76)
OUT0141	Bacteria (100)	Proteobacteria (100)	Betaproteobacteria (100)	Burkholderiales (100)	Comamonadaceae (97)	*Comamonas* (86)
OUT0142	Bacteria (100)	Proteobacteria (100)	Gammaproteobacteria (100)	Alteromonadales (100)	Pseudoalteromonadaceae (93)	*Pseudoalteromonas* (92)
OUT0143	Bacteria (100)	Actinobacteria (99)	Actinobacteria (99)	Pseudonocardiales (99)	Pseudonocardiaceae (86)	unclassified (<50)
OUT0144	Bacteria (100)	Proteobacteria (100)	Gammaproteobacteria (100)	Pseudomonadales (82)	Pseudomonadaceae (82)	*Pseudomonas* (78)
OUT0145	Bacteria (100)	Actinobacteria (100)	Actinobacteria (100)	Pseudonocardiales (100)	Pseudonocardiaceae (87)	unclassified (<50)
OUT0146	Bacteria (100)	Actinobacteria (100)	Actinobacteria (100)	Pseudonocardiales (98)	Pseudonocardiaceae (86)	unclassified (<50)
OUT0147	Bacteria (100)	Proteobacteria (100)	Alphaproteobacteria (100)	Rhizobiales (100)	Methylobacteriaceae (100)	*Methylobacterium* (100)
OUT0148	Bacteria (100)	Bacteroidetes (100)	Bacteroidia (100)	Bacteroidales (100)	Porphyromonadaceae (100)	*Tannerella* (96)
OUT0149	Bacteria (100)	Actinobacteria (100)	Actinobacteria (100)	Pseudonocardiales (100)	Pseudonocardiaceae (83)	unclassified (<50)
OUT0150	Bacteria (100)	Proteobacteria (100)	Gammaproteobacteria (100)	Xanthomonadales (100)	Xanthomonadaceae (100)	*Stenotrophomonas* (90)
OUT0152	Bacteria (100)	Actinobacteria (100)	Actinobacteria (100)	Pseudonocardiales (100)	Pseudonocardiaceae (70)	unclassified (<50)
OUT0153	Bacteria (100)	Firmicutes (100)	Bacilli (100)	Lactobacillales (100)	Streptococcaceae (100)	*Streptococcus* (100)
OUT0154	Bacteria (100)	Actinobacteria (100)	Actinobacteria (100)	Pseudonocardiales (100)	unclassified (<50)	unclassified (<50)
OUT0155	Bacteria (100)	Proteobacteria (100)	Gammaproteobacteria (100)	Pseudomonadales (100)	Moraxellaceae (100)	*Psychrobacter* (65)
OUT0156	Bacteria (100)	Actinobacteria (100)	Actinobacteria (100)	Pseudonocardiales (99)	Pseudonocardiaceae (80)	unclassified (<50)
OUT0157	Bacteria (100)	Proteobacteria (100)	Alphaproteobacteria (100)	Rhodospirillales (100)	Rhodospirillaceae (100)	*Azospirillum* (99)
OUT0158	Bacteria (100)	Actinobacteria (100)	Actinobacteria (100)	Pseudonocardiales (100)	Pseudonocardiaceae (97)	unclassified (<50)
OUT0159	Bacteria (100)	Proteobacteria (100)	Gammaproteobacteria (100)	Vibrionales (92)	Vibrionaceae (92)	*Photobacterium* (55)
OUT0160	Bacteria (100)	Proteobacteria (100)	Alphaproteobacteria (100)	Rhizobiales (100)	Methylobacteriaceae (100)	*Methylobacterium* (100)
OUT0161	Bacteria (100)	Proteobacteria (100)	Gammaproteobacteria (97)	Aeromonadales (80)	Aeromonadaceae (80)	*Aeromonas* (74)
OUT0162	Bacteria (100)	Actinobacteria (98)	Actinobacteria (98)	Pseudonocardiales (98)	Pseudonocardiaceae (76)	unclassified (<50)
OUT0163	Bacteria (100)	Firmicutes (100)	Bacilli (100)	Lactobacillales (100)	Streptococcaceae (99)	*Streptococcus* (99)

OUT0164	Bacteria (100)	Proteobacteria (100)	Gammaproteobacteria (100)	Vibrionales (100)	Vibrionaceae (100)	*Allomonas* (55)
OUT0165	Bacteria (100)	Actinobacteria (100)	Actinobacteria (100)	Pseudonocardiales (100)	Pseudonocardiaceae (100)	*Saccharopolyspora* (67)
OUT0167	Bacteria (100)	Proteobacteria (100)	Gammaproteobacteria (100)	Vibrionales (100)	Vibrionaceae (100)	*Allomonas* (76)
OUT0168	Bacteria (100)	Planctomycets (99)	Planctomycetia (99)	Planctomycetales (99)	Planctomycetaceae (99)	*Blastopirellula* (51)
OUT0169	Bacteria (100)	Actinobacteria (100)	Actinobacteria (100)	Pseudonocardiales (100)	unclassified (<50)	unclassified (<50)
OUT0170	Bacteria (100)	Actinobacteria (100)	Actinobacteria (100)	Pseudonocardiales (100)	Pseudonocardiaceae (87)	unclassified (<50)
OUT0172	Bacteria (100)	Proteobacteria (100)	Gammaproteobacteria (100)	Vibrionales (100)	Vibrionaceae (100)	*Allomonas* (54)
OUT0173	Bacteria (100)	Actinobacteria (100)	Actinobacteria (100)	Pseudonocardiales (100)	Pseudonocardiaceae (80)	unclassified (<50)
OUT0174	Bacteria (100)	Actinobacteria (100)	Actinobacteria (100)	Pseudonocardiales (100)	unclassified (<50)	unclassified (<50)
OUT0175	Bacteria (100)	Firmicutes (97)	Clostridia (96)	Clostridiales (96)	Peptostreptococcaceae (51)	unclassified (<50)
OUT0176	Bacteria (100)	Proteobacteria (100)	Gammaproteobacteria (100)	Pasteurellales (100)	Pasteurellaceae (100)	*Actinobacillus* (61)
OUT0178	Bacteria (100)	Actinobacteria (100)	Actinobacteria (100)	Pseudonocardiales (100)	Pseudonocardiaceae (85)	unclassified (<50)
OUT0179	Bacteria (100)	Proteobacteria (100)	Gammaproteobacteria (100)	Vibrionales (97)	Vibrionaceae (97)	*Photobacterium* (51)
OUT0180	Bacteria (100)	Firmicutes (100)	Bacilli (100)	Lactobacillales (100)	Streptococcaceae (100)	*Streptococcus* (100)
OUT0181	Bacteria (100)	Proteobacteria (100)	Gammaproteobacteria (100)	Pseudomonadales (100)	Moraxellaceae (100)	*Acinetobacter* (100)
OUT0182	Bacteria (100)	Actinobacteria (100)	Actinobacteria (100)	Pseudonocardiales (100)	Pseudonocardiaceae (80)	unclassified (<50)
OUT0183	Bacteria (100)	Firmicutes (97)	Clostridia (92	Clostridiales (92)	unclassified (<50)	unclassified (<50)
OUT0184	Bacteria (100)	Proteobacteria (100)	Alphaproteobacteria (100)	Rhizobiales (91)	unclassified (<50)	unclassified (<50)
OUT0185	Bacteria (100)	Proteobacteria (100)	Gammaproteobacteria (100)	Vibrionales (100)	Vibrionaceae (100)	*Allomonas* (64)
OUT0186	Bacteria (100)	Proteobacteria (100)	Alphaproteobacteria (100)	Rhodobacterales (100)	Rhodobacteraceae (100)	*Silicibacter* (53)
OUT0187	Bacteria (100)	Proteobacteria (100)	Gammaproteobacteria (100)	Aeromonadales (87)	Aeromonadaceae (86)	*Aeromonas* (72)
OUT0188	Bacteria (100)	Proteobacteria (100)	Alphaproteobacteria (100)	Rhodobacterales (100)	Rhodobacteraceae (100)	unclassified (<50)
OUT0189	Bacteria (100)	Proteobacteria (100)	Alphaproteobacteria (100)	Rhizobiales (95)	Phyllobacteriaceae (93)	*Phyllobacterium* (90)

OUT0190	Bacteria (100)	Proteobacteria (100)	Gammaproteobacteria (100)	Vibrionales (100)	Vibrionaceae (100)	*Vibrio* (72)
OUT0191	Bacteria (100)	Proteobacteria (100)	Alphaproteobacteria (100)	Rhodobacterales (96)	Rhodobacteraceae (96)	*Oceanicola* (56)
OUT0192	Bacteria (100)	Proteobacteria (100)	Gammaproteobacteria (100)	Vibrionales (100)	Vibrionaceae (100)	*Lucibacterium* (65)
OUT0193	Bacteria (100)	Proteobacteria (100)	Alphaproteobacteria (100)	Rhodobacterales (96)	Rhodobacteraceae (96)	unclassified (<50)
OUT0194	Bacteria (100)	Proteobacteria (100)	Gammaproteobacteria (100)	Alteromonadales (95)	Shewanellaceae (92)	*Shewanella* (92)
OUT0195	Bacteria (100)	Proteobacteria (100)	Gammaproteobacteria (100)	Vibrionales (100)	Vibrionaceae (100)	*Vibrio* (78)
OUT0196	Bacteria (100)	Firmicutes (100)	Bacilli (100)	Bacillalles (100)	Bacillaceae (96) 1	*Anoxybacillus* (92)
OUT0197	Bacteria (100)	Bacteroidetes (100)	Flavobacteria (100)	Flavobacteriales (100)	Flavobacteriaceae (100)	*Eudoraea* (66)
OUT0198	Bacteria (100)	Bacteroidetes (79)	Bacteroidia (79)	Bacteroidales (79)	Prevotellaceae (78)	*Prevotella* (51)
OUT0199	Bacteria (100)	Actinobacteria (95)	Actinobacteria (95)	Pseudonocardiales (95)	Pseudonocardiaceae (87)	unclassified (<50)
OUT0200	Bacteria (100)	Actinobacteria (100)	Actinobacteria (100)	Pseudonocardiales (100)	unclassified (<50)	unclassified (<50)
OUT0201	Bacteria (100)	Actinobacteria (100)	Actinobacteria (100)	Pseudonocardiales (100)	Pseudonocardiaceae (86)	unclassified (<50)
OUT0202	Bacteria (100)	Actinobacteria (92)	Actinobacteria (92)	Corynebacteriales (92)	unclassified (<50)	unclassified (<50)
OUT0203	Bacteria (100)	Proteobacteria (100)	Gammaproteobacteria (100)	Vibrionales (100)	Vibrionaceae (100)	unclassified (<50)
OUT0204	Bacteria (100)	Proteobacteria (100)	Gammaproteobacteria (100)	Vibrionales (100)	Vibrionaceae (100)	*Allomonas* (52)
OUT0205	Bacteria (100)	Proteobacteria (100)	Gammaproteobacteria (100)	Vibrionales (100)	Vibrionaceae (100)	*Vibrio* (76)
OUT0206	Bacteria (100)	Bacteroidetes (99)	Bacteroidia (97)	Bacteroidales (97)	Porphyromonadaceae (94)	*Porphyromonas* (63)
OUT0207	Bacteria (100)	Actinobacteria (100)	Actinobacteria (100)	Pseudonocardiales (99)	Pseudonocardiaceae (89)	unclassified (<50)
OUT0208	Bacteria (100)	Proteobacteria (100)	Alphaproteobacteria (100)	Rhodobacterales (99)	Rhodobacteraceae (99)	*Phaeobacter* (67)
OUT0209	Bacteria (100)	Proteobacteria (100)	Gammaproteobacteria (100)	Vibrionales (100)	Vibrionaceae (100)	*Vibrio* (63)
OUT0211	Bacteria (100)	Bacteroidetes (100)	Flavobacteria (77)	Flavobacteriales (77)	Cryomorphaceae (60)	unclassified (<50)
OUT0212	Bacteria (100)	Firmicutes (100)	Clostridia (100)	Clostridiales (100)	Clostridiales_Incertae Sedis XI (100)	*Murdochiella* (100)
OUT0213	Bacteria (100)	Proteobacteria (100)	Gammaproteobacteria (100)	Vibrionales (100)	Vibrionaceae (100)	*Vibrio* (74)
OUT0214	Bacteria (100)	Proteobacteria (100)	Gammaproteobacteria (100)	Vibrionales (100)	Vibrionaceae (100)	*vibrio* (55)

**OUT0215**	**Bacteria (100)**	**Proteobacteria (100)**	**Alphaproteobacteria (100)**	**Rhizobiales (90)**	**Phyllobacteriaceae (89)**	***Phyllobacterium* (89)**
**OTU0216**	**Bacteria (100)**	**Proteobacteria (100)**	**Alphaproteobacteria (100)**	**Rhizobiales (80)**	**Phyllobacteriaceae (80)**	***Phyllobacterium* (78)**
**OTU0217**	**Bacteria (100)**	**Actinobacteria (100)**	**Actinobacteria (100)**	**Micrococcales (100)**	**Brevibacteriaceae (100)**	***Brevibacterium* (100)**
**OTU0218**	**Bacteria (100)**	**Proteobacteria (100)**	**Alphaproteobacteria (100)**	**Sphingomonadales (99)**	**Sphingomonadaceae (98)**	***Sphingomonas* (98)**
**OTU0219**	**Bacteria (100)**	**Firmicutes (100)**	**Clostridia (100)**	**Clostridiales (100)**	**Clostridiales_Incertae Sedis XI (100)**	***Anaerococcus* (100)**
**OTU0220**	**Bacteria (100)**	**Proteobacteria (100)**	**Alphaproteobacteria (100)**	**Rhizobiales (83)**	**Phyllobacteriaceae (80)**	***Phyllobacterium* (79)**
**OTU0221**	**Bacteria (100)**	**Cyanobacteria/ Chloroplast(61)**	**unclassified (<50)**	**unclassified (<50)**	**unclassified (<50)**	**unclassified (<50)**
**OTU0222**	**Bacteria (100)**	**Actinobacteria (98)**	**Actinobacteria (98)**	**Pseudonocardiales (98)**	**Pseudonocardiaceae (75)**	**unclassified (<50)**
**OTU0223**	**Bacteria (100)**	**Proteobacteria (100)**	**Gammaproteobacteria (100)**	**Alteromonadales (70)**	**unclassified (<50)**	**unclassified (<50)**
**OTU0224**	**Bacteria (100)**	**Actinobacteria (100)**	**Actinobacteria (100)**	**Pseudonocardiales (100)**	**Pseudonocardiaceae (87)**	**unclassified (<50)**
**OTU0225**	**Bacteria (100)**	**Proteobacteria (100)**	**Gammaproteobacteria (100)**	**Vibrionales (100)**	**Vibrionaceae (100)**	**unclassified (<50)**
**OTU0226**	**Bacteria (100)**	**Proteobacteria (100)**	**Gammaproteobacteria (100)**	**Vibrionales (100)**	**Vibrionaceae (100)**	***Vibrio* (75)**
**OTU0227**	**Bacteria (100)**	**Proteobacteria (100)**	**Betaproteobacteria (100)**	**Burkholderiales (100)**	**Burkholderiales_incertae Sedis (97)**	***Sphaerotilus* (97)**
**OUT0228**	**Bacteria (100)**	**Actinobacteria (100)**	**Actinobacteria (100)**	**Pseudonocardiales (100)**	**Pseudonocardiaceae (90)**	**unclassified (<50)**
**OUT0229**	**Bacteria (100)**	**Actinobacteria (100)**	**Actinobacteria (100)**	**Pseudonocardiales (99)**	**Pseudonocardiaceae (70)**	**unclassified (<50)**
**OUT0230**	**Bacteria (100)**	**Proteobacteria (100)**	**Gammaproteobacteria (99)**	**Pseudomonadales (99)**	**Pseudomonadaceae (99)**	***Pseudomonas* (50)**
**OUT0231**	**Bacteria (100)**	**Firmicutes (100)**	**Bacilli (100)**	**Lactobacillales (99)**	**Carnobacteriaceae (97)**	***Granulicatella* (92)**
**OUT0232**	**Bacteria (100)**	**Actinobacteria (100)**	**Actinobacteria (100)**	**Pseudonocardiales (100)**	**Pseudonocardiaceae (88)**	**unclassified (<50)**
**OUT0235**	**Bacteria (100)**	**Actinobacteria (100)**	**Actinobacteria (100)**	**Pseudonocardiales (100)**	**Pseudonocardiaceae (76)**	**unclassified (<50)**
**OUT0236**	**Bacteria (100)**	**Proteobacteria (100)**	**Alphaproteobacteria (100)**	**Rhodobacterales (100)**	**Rhodobacteraceae (100)**	***Donghicola* (59)**
**OUT0237**	**Bacteria (100)**	**Planctomycets (97)**	**Planctomycetia (97)**	**Planctomycetales (97)**	**Planctomycetaceae (97)**	***Planctomyces* (78)**
**OUT0238**	**Bacteria (100)**	**Bacteroidetes (100)**	**Flavobacteria (100)**	**Flavobacteriales (100)**	**Flavobacteriaceae (100)**	***Capnocytophaga* (100)**
**OUT0239**	**Bacteria (100)**	**Proteobacteria (100)**	**Gammaproteobacteria (100)**	**Vibrionales (100)**	**Vibrionaceae (100)**	***Vibrio* (68)**
**OUT0240**	**Bacteria (100)**	**Actinobacteria (100)**	**Actinobacteria (100)**	**Actinomycetales (100)**	**Actinomycetaceae (100)**	***Actinomyces* (100)**

**OTU0242**	**Bacteria (100)**	**Actinobacteria (100)**	**Actinobacteria (100)**	**Pseudonocardiales (100)**	**Pseudonocardiaceae (97)**	**unclassified (<50)**
**OTU0243**	**Bacteria (100)**	**Firmicutes (100)**	**Negativicutes (100)**	**Selenomonadales (100)**	**Veillonellaceae (100)**	***Veillonella* (100)**
**OTU0246**	**Bacteria (100)**	**Proteobacteria (100)**	**Gammaproteobacteria (100)**	**Alteromonadales (99)**	**Shewanellaceae (99)**	***Shewanella* (99)**
**OTU0251**	**Bacteria (100)**	**Proteobacteria (100)**	**Gammaproteobacteria (100)**	**Oceanospirillales (100)**	**Oceanospirillaceae (100)**	***Marinomonas* (99)**
**OTU0252**	**Bacteria (100)**	**Actinobacteria (100)**	**Actinobacteria (100)**	**Micrococcales (100)**	**Microbacteriaceae (100)**	***Zimmermannella* (81)**
**OTU0253**	**Bacteria (100)**	**Proteobacteria (100)**	**Gammaproteobacteria (100)**	**Oceanospirillales (100)**	**Oceanospirillaceae (100)**	***Marinomonas* (100)**
**OTU0254**	**Bacteria (100)**	**Actinobacteria (99)**	**Actinobacteria (99)**	**Pseudonocardiales (99)**	**Pseudonocardiaceae (83)**	**unclassified (<50)**
**OTU0255**	**Bacteria (100)**	**Proteobacteria (100)**	**Gammaproteobacteria (100)**	**Aeromonadales (90)**	**Aeromonadaceae (90)**	***Aeromonas* (90)**
**OTU0256**	**Bacteria (100)**	**Actinobacteria (100)**	**Actinobacteria (100)**	**Pseudonocardiales (99)**	**Pseudonocardiaceae (93)**	**unclassified (<50)**
**OTU0257**	**Bacteria (100)**	**Proteobacteria (100)**	**Gammaproteobacteria (100)**	**Xanthomonadales (100)**	**Xanthomonadaceae (100)**	***Xanthomonas* (99)**
**OTU0258**	**Bacteria (100)**	**Proteobacteria (100)**	**Gammaproteobacteria (100)**	**Vibrionales (100)**	**Vibrionaceae (100)**	***Allomonas* (60)**
**OTU0259**	**Bacteria (100)**	**Proteobacteria (100)**	**Gammaproteobacteria (100)**	**Vibrionales (100)**	**Vibrionaceae (100)**	***Listonella* (65)**
**OTU0261**	**Bacteria (100)**	**Proteobacteria (100)**	**Gammaproteobacteria (100)**	**Vibrionales (94)**	**Vibrionaceae (94)**	***Salinivibrio* (52)**
**OTU0263**	**Bacteria (100)**	**Proteobacteria (100)**	**Gammaproteobacteria (100)**	**Chromatiales (98)**	**Chromatiaceae (98)**	***Rheinheimera* (98)**
**OTU0264**	**Bacteria (100)**	**Proteobacteria (100)**	**Betaproteobacteria (100)**	**Burkholderiales (100)**	**Comamonadaceae (95)**	***Delftia* (76)**
**OTU0265**	**Bacteria (100)**	**Proteobacteria (100)**	**Gammaproteobacteria (99)**	**Pseudomonadales (99)**	**Moraxellaceae (99)**	***Acinetobacter* (99)**
**OTU0268**	**Bacteria (100)**	**Proteobacteria (100)**	**Alphaproteobacteria (100)**	**Rhodobacterales (100)**	**Rhodobacteraceae (100)**	**unclassified (<50)**
**OTU0269**	**Bacteria (100)**	**Proteobacteria (100)**	**Gammaproteobacteria (100)**	**Chromatiales (96)**	**Chromatiaceae (96)**	***Rheinheimera* (96)**
**OTU0270**	**Bacteria (100)**	**Actinobacteria (100)**	**Actinobacteria (100)**	**Micrococcales (100)**	**Micrococcaceae (100)**	***Nesterenkonia* (81)**
**OTU0271**	**Bacteria (100)**	**Actinobacteria (100)**	**Actinobacteria (100)**	**Pseudonocardiales (99)**	**Pseudonocardiaceae (90)**	**unclassified (<50)**
**OTU0272**	**Bacteria (100)**	**Bacteroidetes (99)**	**Bacteroidia (98)**	**Bacteroidales (98)**	**Porphyromonadaceae (97)**	***Porphyromonas* (79)**
**OTU0273**	**Bacteria (100)**	**Actinobacteria (100)**	**Actinobacteria (100)**	**Pseudonocardiales (100)**	**Pseudonocardiaceae (82)**	**unclassified (<50)**
**OTU0274**	**Bacteria (100)**	**Proteobacteria (100)**	**Gammaproteobacteria (100)**	**Vibrionales (99)**	**Vibrionaceae (99)**	***Allomonas* (94)**
**OTU0275**	**Bacteria (100)**	**Actinobacteria (100)**	**Actinobacteria (100)**	**Pseudonocardiales (100)**	**Pseudonocardiaceae (90)**	**unclassified (<50)**

**OTU0276**	**Bacteria (100)**	**Actinobacteria (100)**	**Actinobacteria (100)**	**Micrococcales (100)**	**Micrococcaceae (97)**	***Nesterenkonia* (54)**
**OUT0277**	**Bacteria (100)**	**Actinobacteria (100)**	**Actinobacteria (100)**	**Pseudonocardiales (100)**	**Pseudonocardiaceae (87)**	**unclassified (<50)**
**OUT0280**	**Bacteria (100)**	**Actinobacteria (100)**	**Actinobacteria (100)**	**Pseudonocardiales (100)**	**Pseudonocardiaceae (87)**	**unclassified (<50)**
**OUT0281**	**Bacteria (100)**	**Actinobacteria (100)**	**Actinobacteria (100)**	**Corynebacteriales (100)**	**Corynebacteriaceae (94)**	***Nesterenkonia* (87)**
**OUT0283**	**Bacteria (100)**	**Proteobacteria (100)**	**Gammaproteobacteria (100)**	**Vibrionales (100)**	**Vibrionaceae (100)**	***Allomonas* (51)**
**OUT0284**	**Bacteria (100)**	**Proteobacteria (100)**	**Betaproteobacteria (100)**	**Rhodocyclales (94)**	**Rhodocyclaceae (94)**	**unclassified (<50)**
**OUT0285**	**Bacteria (100)**	**Proteobacteria (100)**	**Alphaproteobacteria (100)**	**Rhizobiales (85)**	**Phyllobacteriaceae (82)**	***Phyllobacterium* (82)**
**OUT0288**	**Bacteria (100)**	**Actinobacteria (100)**	**Actinobacteria (100)**	**Pseudonocardiales (100)**	**Pseudonocardiaceae (95)**	**unclassified (<50)**
**OUT0289**	**Bacteria (100)**	**Proteobacteria (100)**	**Gammaproteobacteria (100)**	**Oceanospirillales (98)**	**Oceanospirillaceae (98)**	***Marinomonas* (98)**
**OUT0290**	**Bacteria (100)**	**Proteobacteria (100)**	**Gammaproteobacteria (100)**	**Vibrionales (100)**	**Vibrionaceae (100)**	***Allomonas* (78)**
**OUT0291**	**Bacteria (100)**	**Actinobacteria (100)**	**Actinobacteria (100)**	**Pseudonocardiales (100)**	**Pseudonocardiaceae (86)**	**unclassified (<50)**
**OUT0293**	**Bacteria (100)**	**Bacteroidetes (100)**	**Bacteroidia (99)**	**Bacteroidales (99)**	**Porphyromonadaceae (95)**	***Porphyromonas* (70)**
**OUT0294**	**Bacteria (100)**	**Actinobacteria (99)**	**Actinobacteria (99)**	**Pseudonocardiales (99)**	**Pseudonocardiaceae (78)**	**unclassified (<50)**
**OUT0295**	**Bacteria (100)**	**Proteobacteria (100)**	**Gammaproteobacteria (100)**	**Pseudomonadales (100)**	**Moraxellaceae (100)**	***Acinetobacter* (100)**
**OUT0296**	**Bacteria (99)**	**Bacteroidetes (82)**	**Bacteroidia (77)**	**Bacteroidales (77)**	**Prevotellaceae (64)**	***Prevotella* (56)**
**OUT0297**	**Bacteria (100)**	**Planctomycets (88)**	**Planctomycetia (88)**	**Planctomycetales (88)**	**Planctomycetaceae (88)**	***Pirellula* (56)**
**OUT0298**	**Bacteria (100)**	**Bacteroidetes (100)**	**Flavobacteria (98)**	**Flavobacteriales (98)**	**Cryomorphaceae (98)**	**unclassified (<50)**
**OUT0299**	**Bacteria (100)**	**Actinobacteria (100)**	**Actinobacteria (100)**	**Corynebacteriales (100)**	**Corynebacteriaceae (94)**	***Corynebacterium* (91)**
**OUT0300**	**Bacteria (100)**	**Actinobacteria (100)**	**Actinobacteria (100)**	**Pseudonocardiales (100)**	**Pseudonocardiaceae (59)**	**unclassified (<50)**
**OUT0301**	**Bacteria (100)**	**Proteobacteria (100)**	**Alphaproteobacteria (100)**	**Caulobacterales (100)**	**Caulobacteraceae (100)**	***Brevundimonas* (100)**
**OUT0302**	**Bacteria (100)**	**Proteobacteria (100)**	**Gammaproteobacteria (100)**	**Vibrionales (95)**	**Vibrionaceae (95)**	***Listonella* (55)**
**OUT0303**	**Bacteria (100)**	**Proteobacteria (100)**	**Alphaproteobacteria (100)**	**Rhodobacterales (88)**	**Rhodobacteraceae (99)**	***Oceanicola* (84)**
**OUT0304**	**Bacteria (100)**	**Proteobacteria (100)**	**Gammaproteobacteria (73)**	**Alteromonadales (70)**	**Pseudoalteromonadaceae (59)**	***Pseudoalteromonas* (56)**
**OUT0305**	**Bacteria (100)**	**Firmicutes (100)**	**Negativicutes (98)**	**Selenomonadales (98)**	**Veillonellaceae (98)**	***Veillonella* (87)**

**OUT0306**	**Bacteria (100)**	**Proteobacteria (100)**	**Gammaproteobacteria (100)**	**Pseudomonadales (99)**	**Moraxellaceae (99)**	***Acinetobacter* (98)**
**OTU0307**	**Bacteria (100)**	**Firmicutes (99)**	**Clostridia (99**	**Clostridiales (98)**	**Ruminococcaceae (89)**	***Clostridium IV* (69)**
**OTU0308**	**Bacteria (100)**	**Proteobacteria (100)**	**Gammaproteobacteria (100)**	**Pseudomonadales (100)**	**Moraxellaceae (100)**	***Acinetobacter* (98)**
**OTU0309**	**Bacteria (100)**	**Proteobacteria (100)**	**Gammaproteobacteria (100)**	**Vibrionales (100)**	**Vibrionaceae (100)**	***Lucibacterium* (60)**
**OTU0310**	**Bacteria (100)**	**Proteobacteria (100)**	**Gammaproteobacteria (100)**	**Vibrionales (93)**	**Vibrionaceae (93)**	***Photobacterium* (62)**
**OTU0311**	**Bacteria (100)**	**Proteobacteria (100)**	**Alphaproteobacteria (99)**	**Rhizobiales (99)**	**Hyphomicrobiaceae (65)**	***Rhodoplanes* (58)**
**OTU0312**	**Bacteria (100)**	**Actinobacteria (100)**	**Actinobacteria (100)**	**Pseudonocardiales (99)**	**unclassified (<50)**	**unclassified (<50)**
**OTU0313**	**Bacteria (100)**	**Firmicutes (100)**	**Bacilli (100)**	**Lactobacillales (100)**	**Carnobacteriaceae (100)**	***Dolosigranulum* (100)**
**OTU0314**	**Bacteria (100)**	**Proteobacteria (100)**	**Gammaproteobacteria (100)**	**Vibrionales (100)**	**Vibrionaceae (100)**	***Allomonas* (81)**
**OTU0315**	**Bacteria (100)**	**Actinobacteria (100)**	**Actinobacteria (100)**	**Micrococcales (100)**	**unclassified (<50)**	**unclassified (<50)**
**OTU0316**	**Bacteria (100)**	**Firmicutes (100)**	**Bacilli (100)**	**Bacillalles (100)**	**Bacillales_Incertae Sedis XII (100)**	***Gemella* (100)**
**OTU0317**	**Bacteria (100)**	**Proteobacteria (100)**	**Gammaproteobacteria (100)**	**Vibrionales (98)**	**Vibrionaceae (98)**	***Allomonas* (81)**
**OTU0318**	**Bacteria (100)**	**Firmicutes (100)**	**Bacilli (100)**	**Lactobacillales (100)**	**Aerococcaceae (100)**	***Aerococcus* (100)**
**OTU0321**	**Bacteria (100)**	**Firmicutes (100)**	**Bacilli (100)**	**Lactobacillales (100)**	**Lactobacillaceae (95)**	***Lactobacillus* (94)**
**OTU0322**	**Bacteria (100)**	**Proteobacteria (100)**	**Gammaproteobacteria (100)**	**Vibrionales (100)**	**Vibrionaceae (100)**	**unclassified (<50)**
**OTU0323**	**Bacteria (100)**	**Actinobacteria (100)**	**Actinobacteria (100)**	**Actinomycetales (100)**	**Actinomycetaceae (100)**	***Varibaculum* (100)**
**OTU0324**	**Bacteria (100)**	**Proteobacteria (100)**	**Betaproteobacteria (100)**	**Neisseriales (100)**	**Neisseriaceae (100)**	***Neisseria* (100)**
**OTU0325**	**Bacteria (100)**	**Firmicutes (100)**	**Bacilli (100)**	**Bacillales (98)**	**Staphylococcaceae (93)**	***Staphylococcus* (89)**
**OTU0326**	**Bacteria (100)**	**Proteobacteria (100)**	**Gammaproteobacteria (100)**	**Vibrionales (100)**	**Vibrionaceae (100)**	***Vibrio* (60)**
**OTU0327**	**Bacteria (100)**	**Proteobacteria (100)**	**Gammaproteobacteria (100)**	**Vibrionales (99)**	**Vibrionaceae (99)**	**unclassified (<50)**
**OTU0328**	**Bacteria (100)**	**Actinobacteria (100)**	**Actinobacteria (100)**	**Pseudonocardiales (100)**	**Pseudonocardiaceae (70)**	**unclassified (<50)**
**OTU0329**	**Bacteria (100)**	**Firmicutes (100)**	**Clostridia (100)**	**Clostridiales (100)**	**Peptostreptococcaceae (100)**	***Peptostreptococcus* (100)**
**OTU0330**	**Bacteria (100)**	**Actinobacteria (99)**	**Actinobacteria (99)**	**Pseudonocardiales (98)**	**unclassified (<50)**	**unclassified (<50)**

**OTU0332**	**Bacteria (100)**	**Proteobacteria (100)**	**Gammaproteobacteria (100)**	**Vibrionales (96)**	**Vibrionaceae (96)**	***Vibrio* (64)**
**OTU0334**	**Bacteria (100)**	**Actinobacteria (100)**	**Actinobacteria (100)**	**Pseudonocardiales (100)**	**Pseudonocardiaceae (77)**	**unclassified (<50)**
**OTU0335**	**Bacteria (100)**	**Firmicutes (100)**	**Bacilli (100)**	**Bacillalles (100)**	**Bacillaceae (78) 1**	***Anoxybacillus* (68)**
**OTU0337**	**Bacteria (100)**	**Actinobacteria (99)**	**Actinobacteria (99)**	**Pseudonocardiales (99)**	**Pseudonocardiaceae (72)**	**unclassified (<50)**
**OTU0338**	**Bacteria (100)**	**Proteobacteria (100)**	**Gammaproteobacteria (100)**	**Pseudomonadales (99)**	**Pseudomonadaceae (99)**	**unclassified (<50)**
**OTU0339**	**Bacteria (100)**	**Bacteroidetes (100)**	**Bacteroidia (100)**	**Bacteroidales (100)**	**Prevotellaceae (100)**	***Prevotella* (100)**
**OTU0340**	**Bacteria (100)**	**Proteobacteria (100)**	**Gammaproteobacteria (100)**	**Vibrionales (100)**	**Vibrionaceae (100)**	**unclassified (<50)**
**OTU0341**	**Bacteria (100)**	**Proteobacteria (100)**	**Gammaproteobacteria (100)**	**Oceanospirillales (100)**	**Oceanospirillaceae (100)**	***Marinomonas* (100)**
**OTU0342**	**Bacteria (100)**	**Actinobacteria (99)**	**Actinobacteria (99)**	**Pseudonocardiales (99)**	**unclassified (<50)**	**unclassified (<50)**
**OTU0343**	**Bacteria (100)**	**Firmicutes (97)**	**Bacilli (97)**	**Bacillales (93)**	**Staphylococcaceae (89)**	***Staphylococcus* (85)**
**OTU0344**	**Bacteria (100)**	**Actinobacteria (100)**	**Actinobacteria (100)**	**Pseudonocardiales (100)**	**Pseudonocardiaceae (92)**	**unclassified (<50)**
**OTU0345**	**Bacteria (100)**	**Proteobacteria (100)**	**Gammaproteobacteria (100)**	**Vibrionales (100)**	**Vibrionaceae (100)**	***Allomonas* (57)**
**OTU0346**	**Bacteria (100)**	**Proteobacteria (100)**	**Gammaproteobacteria (100)**	**Aeromonadales (73)**	**Aeromonadaceae (73)**	***Aeromonas* (71)**
**OTU0347**	**Bacteria (100)**	**Proteobacteria (100)**	**Gammaproteobacteria (100)**	**Chromatiales (99)**	**Chromatiaceae (99)**	***Rheinheimera* (99)**
**OTU0348**	**Bacteria (100)**	**Proteobacteria (99)**	**Gammaproteobacteria (99)**	**Xanthomonadales (88)**	**Xanthomonadaceae (88)**	***Pseudoxanthomonas* (75)**
**OTU0349**	**Bacteria (100)**	**Proteobacteria (100)**	**Alphaproteobacteria**	**Rhizobiales (57)**	**Phyllobacteriaceae (54)**	**unclassified (<50)**
**OTU0350**	**Bacteria (100)**	**Proteobacteria (100)**	**Alphaproteobacteria (100)**	**Rhodobacterales (98)**	**Rhodobacteraceae (98)**	**unclassified (<50)**
**OTU0353**	**Bacteria (100)**	**Proteobacteria (100)**	**Gammaproteobacteria (100)**	**Vibrionales (100)**	**Vibrionaceae (100)**	***Allomonas* (72)**
**OTU0354**	**Bacteria (100)**	**Proteobacteria (100)**	**Gammaproteobacteria (100)**	**Vibrionales (97)**	**Vibrionaceae (97)**	***Photobacterium* (50)**
**OTU0356**	**Bacteria (100)**	**Proteobacteria (100)**	**Gammaproteobacteria (100)**	**Vibrionales (100)**	**Vibrionaceae (100)**	***Allomonas* (76)**
**OTU0358**	**Bacteria (100)**	**Actinobacteria (98)**	**Actinobacteria (98)**	**Pseudonocardiales (98)**	**Pseudonocardiaceae (90)**	**unclassified (<50)**
**OTU0359**	**Bacteria (100)**	**Proteobacteria (100)**	**Gammaproteobacteria (100)**	**Pseudomonadales (100)**	**Moraxellaceae (100)**	***Acinetobacter* (100)**
**OTU0360**	**Bacteria (100)**	**Firmicutes (100)**	**Negativicutes (100)**	**Selenomonadales (100)**	**Veillonellaceae (100)**	***Selenomonas* (90)**
**OTU0361**	**Bacteria (100)**	**Proteobacteria (100)**	**Alphaproteobacteria (100)**	**Rhizobiales (87)**	**Phyllobacteriaceae (85)**	***Phyllobacterium* (85)**

**OTU0362**	**Bacteria (100)**	**Proteobacteria (100)**	**Gammaproteobacteria (100)**	**Vibrionales (100)**	**Vibrionaceae (100)**	***vibrio* (55)**
**OTU0363**	**Bacteria (100)**	**Proteobacteria (100)**	**Gammaproteobacteria (100)**	**Vibrionales (100)**	**Vibrionaceae (100)**	***Listonella* (87)**
**OTU0364**	**Bacteria (100)**	**Proteobacteria (100)**	**Alphaproteobacteria (100)**	**Rhodobacterales (91)**	**Rhodobacteraceae (91)**	**unclassified (<50)**
**OTU0365**	**Bacteria (100)**	**Proteobacteria (100)**	**Betaproteobacteria (100)**	**Burkholderiales (100)**	**Alcaligenaceae (100)**	***Achromobacter* (64)**
**OTU0366**	**Bacteria (100)**	**Actinobacteria (100)**	**Actinobacteria (100)**	**Pseudonocardiales (100)**	**Pseudonocardiaceae (93)**	**unclassified (<50)**
**OTU0367**	**Bacteria (100)**	**Cyanobacteria/ Chloroplast(56)**	**unclassified (<50)**	**unclassified (<50)**	**unclassified (<50)**	**unclassified (<50)**
**OTU0368**	**Bacteria (100)**	**Actinobacteria (100)**	**Actinobacteria (100)**	**Pseudonocardiales (100)**	**Pseudonocardiaceae (93)**	**unclassified (<50)**
**OTU0369**	**Bacteria (100)**	**Actinobacteria (99)**	**Actinobacteria (99)**	**Pseudonocardiales (99)**	**Pseudonocardiaceae (77)**	**unclassified (<50)**
**OTU0371**	**Bacteria (100)**	**Proteobacteria (100)**	**Gammaproteobacteria (100)**	**Vibrionales (99)**	**Vibrionaceae (99)**	***Listonella* (84)**
**OTU0372**	**Bacteria (100)**	**Actinobacteria (99)**	**Actinobacteria (99)**	**Pseudonocardiales (99)**	**Pseudonocardiaceae (86)**	**unclassified (<50)**
**OTU0373**	**Bacteria (100)**	**Actinobacteria (100)**	**Actinobacteria (100)**	**Pseudonocardiales (100)**	**Pseudonocardiaceae (70)**	**unclassified (<50)**
**OTU0374**	**Bacteria (100)**	**Proteobacteria (100)**	**Gammaproteobacteria (100)**	**Vibrionales (99)**	**Vibrionaceae (99)**	**unclassified (<50)**
**OTU0376**	**Bacteria (100)**	**Proteobacteria (100)**	**Betaproteobacteria (100)**	**Burkholderiales (100)**	**Comamonadaceae (100)**	***Schlegelella* (92)**
**OTU0377**	**Bacteria (100)**	**Proteobacteria (100)**	**Gammaproteobacteria (100)**	**Vibrionales (100)**	**Vibrionaceae (100)**	***Allomonas* (84)**
**OTU0378**	**Bacteria (100)**	**Proteobacteria (100)**	**Alphaproteobacteria (100)**	**Rhodobacterales (97)**	**Rhodobacteraceae (97)**	**unclassified (<50)**
**OTU0379**	**Bacteria (100)**	**Actinobacteria (100)**	**Actinobacteria (100)**	**Actinomycetales (100)**	**Actinomycetaceae (100)**	***Actinomyces* (100)**
**OTU0380**	**Bacteria (100)**	**Proteobacteria (100)**	**Gammaproteobacteria (100)**	**Vibrionales (94)**	**Vibrionaceae (94)**	***Photobacterium* (69)**
**OTU0382**	**Bacteria (100)**	**Proteobacteria (100)**	**Gammaproteobacteria (100)**	**Vibrionales (92)**	**Vibrionaceae (92)**	***Salinivibrio* (58)**
**OTU0384**	**Bacteria (100)**	**Actinobacteria (100)**	**Actinobacteria (100)**	**Pseudonocardiales (100)**	**Pseudonocardiaceae (78)**	**unclassified (<50)**
**OTU0385**	**Bacteria (100)**	**Proteobacteria (100)**	**Gammaproteobacteria (100)**	**Vibrionales (100)**	**Vibrionaceae (100)**	***Allomonas* (75)**
**OTU0386**	**Bacteria (100)**	**Proteobacteria (100)**	**Alphaproteobacteria (100)**	**Rhizobiales (93)**	**unclassified (<50)**	**unclassified (<50)**
**OTU0387**	**Bacteria (100)**	**Actinobacteria (100)**	**Actinobacteria (100)**	**Pseudonocardiales (98)**	**Pseudonocardiaceae (85)**	**unclassified (<50)**
**OTU0388**	**Bacteria (100)**	**Proteobacteria (100)**	**Alphaproteobacteria (100)**	**Rhodobacterales (98)**	**Rhodobacteraceae (98)**	**unclassified (<50)**
**OTU0389**	**Bacteria (100)**	**Actinobacteria (100)**	**Actinobacteria (100)**	**Corynebacteriales (100)**	**Corynebacteriaceae (94)**	***Corynebacterium* (90)**

OTU0390	Bacteria (100)	Actinobacteria (100)	Actinobacteria (100)	Pseudonocardiales (100)	Pseudonocardiaceae (96)	unclassified (<50)
OUT0391	Bacteria (100)	Proteobacteria (100)	Gammaproteobacteria (100)	Alteromonadales (99)	Pseudoalteromonadaceae (99)	*Pseudoalteromonas* (97)
OUT0393	Bacteria (100)	Actinobacteria (80)	Actinobacteria (80)	Pseudonocardiales (79)	Pseudonocardiaceae (66)	unclassified (<50)
OUT0394	Bacteria (100)	Firmicutes (100)	Bacilli (100)	Lactobacillales (100)	Streptococcaceae (100)	*Streptococcus* (100)
OUT0395	Bacteria (100)	Proteobacteria (100)	Gammaproteobacteria (100)	unclassified (<50)	unclassified (<50)	unclassified (<50)
OUT0396	Bacteria (100)	Proteobacteria (100)	Gammaproteobacteria (100)	Xanthomonadales (100)	Xanthomonadaceae (100)	*Pseudoxanthomonas* (100)
OUT0397	Bacteria (100)	Proteobacteria (100)	Alphaproteobacteria (100)	Rhodobacterales (98)	Rhodobacteraceae (98)	unclassified (<50)
OUT0398	Bacteria (100)	Proteobacteria (100)	Gammaproteobacteria (100)	Vibrionales (89)	Vibrionaceae (89)	unclassified (<50)
OUT0399	Bacteria (100)	Proteobacteria (100)	Gammaproteobacteria (100)	Chromatiales (100)	Chromatiaceae (100)	*Rheinheimera* (100)
OUT0400	Bacteria (100)	Proteobacteria (100)	Betaproteobacteria (100)	Burkholderiales (99)	Comamonadaceae (99)	*Acidovorax* (87)
OUT0402	Bacteria (100)	Proteobacteria (100)	Gammaproteobacteria (100)	Pseudomonadales (77)	Moraxellaceae (77)	*Acinetobacter* (71)
OUT0403	Bacteria (100)	Firmicutes (100)	Bacilli (100)	Lactobacillales (100)	Streptococcaceae (100)	*Streptococcus* (100)
OUT0404	Bacteria (100)	Actinobacteria (99)	Actinobacteria (99)	Pseudonocardiales (98)	Pseudonocardiaceae (69)	unclassified (<50)
OUT0405	Bacteria (100)	Proteobacteria (100)	Gammaproteobacteria (100)	Vibrionales (100)	Vibrionaceae (100)	*Allomonas* (71)
OUT0406	Bacteria (100)	Actinobacteria (100)	Actinobacteria (100)	Pseudonocardiales (99)	Pseudonocardiaceae (90)	unclassified (<50)
OUT0407	Bacteria (100)	Proteobacteria (100)	Gammaproteobacteria (100)	Vibrionales (100)	Vibrionaceae (100)	*Vibrio* (88)
OUT0408	Bacteria (100)	Firmicutes (100)	Bacilli (100)	Lactobacillales (100)	Streptococcaceae (100)	*Streptococcus* (100)
OUT0409	Bacteria (100)	Proteobacteria (100)	Gammaproteobacteria (100)	Aeromonadales (100)	Aeromonadaceae (100)	*Aeromonas* (100)
OUT0410	Bacteria (100)	Actinobacteria (100)	Actinobacteria (100)	Pseudonocardiales (100)	Pseudonocardiaceae (82)	unclassified (<50)
OUT0412	Bacteria (100)	Proteobacteria (100)	Gammaproteobacteria (100)	Pseudomonadales (100)	Moraxellaceae (100)	*Acinetobacter* (100)
OUT0414	Bacteria (100)	Proteobacteria (100)	Gammaproteobacteria (99)	Vibrionales (88)	Vibrionaceae (88)	*Allomonas* (74)
OUT0415	Bacteria (100)	Proteobacteria (99)	Gammaproteobacteria (99)	Alteromonadales (99)	Pseudoalteromonadaceae (88)	*Pseudoalteromonas* (85)
OUT0416	Bacteria (100)	Actinobacteria (100)	Actinobacteria (100)	Corynebacteriales (100)	Corynebacteriaceae (89)	*Corynebacterium* (78)
OUT0417	Bacteria (100)	Proteobacteria (100)	Gammaproteobacteria (94)	Alteromonadales (94)	Pseudoalteromonadaceae (88)	*Pseudoalteromonas* (81)

**OUT0418**	**Bacteria (100)**	**Actinobacteria (100)**	**Actinobacteria (100)**	**Pseudonocardiales (100)**	**Pseudonocardiaceae (94)**	**unclassified (<50)**
**OUT0419**	**Bacteria (100)**	**Proteobacteria (100)**	**Gammaproteobacteria (100)**	**Vibrionales (100)**	**Vibrionaceae (100)**	***Allomonas* (53)**
**OUT0420**	**Bacteria (100)**	**Actinobacteria (100)**	**Actinobacteria (100)**	**Pseudonocardiales (100)**	**Pseudonocardiaceae (72)**	**unclassified (<50)**
**OUT0421**	**Bacteria (100)**	**Proteobacteria (100)**	**Gammaproteobacteria (100)**	**Pseudomonadales (100)**	**Moraxellaceae (100)**	***Acinetobacter* (95)**
**OUT0422**	**Bacteria (100)**	**Proteobacteria (100)**	**Betaproteobacteria (100)**	**Burkholderiales (100)**	**Comamonadaceae (92)**	**unclassified (<50)**
**OUT0423**	**Bacteria (100)**	**Proteobacteria (100)**	**Alphaproteobacteria (100)**	**Rhodobacterales (99)**	**Rhodobacteraceae (99)**	**unclassified (<50)**
**OUT0424**	**Bacteria (100)**	**Actinobacteria (100)**	**Actinobacteria (100)**	**Pseudonocardiales (100)**	**Pseudonocardiaceae (100)**	***Actinomycetospora* (99)**
**OUT0425**	**Bacteria (100)**	**Proteobacteria (100)**	**Alphaproteobacteria (100)**	**Rhizobiales (96)**	**Phyllobacteriaceae (95)**	***Phyllobacterium* (93)**
**OUT0426**	**Bacteria (100)**	**Actinobacteria (100)**	**Actinobacteria (100)**	**Pseudonocardiales (97)**	**Pseudonocardiaceae (88)**	**unclassified (<50)**
**OUT0427**	**Bacteria (100)**	**Actinobacteria (100)**	**Actinobacteria (100)**	**Pseudonocardiales (99)**	**Pseudonocardiaceae (97)**	**unclassified (<50)**
**OUT0428**	**Bacteria (100)**	**Proteobacteria (100)**	**Gammaproteobacteria (100)**	**Pseudomonadales (100)**	**Moraxellaceae (100)**	***Acinetobacter* (100)**
**OUT0429**	**Bacteria (100)**	**Proteobacteria (100)**	**Gammaproteobacteria (100)**	**Vibrionales (95)**	**Vibrionaceae (95)**	**unclassified (<50)**
**OUT0430**	**Bacteria (100)**	**Proteobacteria (100)**	**Gammaproteobacteria (100)**	**Vibrionales (98)**	**Vibrionaceae (98)**	**unclassified (<50)**
**OUT0431**	**Bacteria (100)**	**Proteobacteria (100)**	**Gammaproteobacteria (100)**	**Enterobacteriales (100)**	**Enterobacteriaceae (100)**	**unclassified (<50)**
**OUT0432**	**Bacteria (100)**	**Proteobacteria (100)**	**Gammaproteobacteria (100)**	**Vibrionales (100)**	**Vibrionaceae (100)**	***Allomonas* (51)**
**OUT0433**	**Bacteria (100)**	**Proteobacteria (100)**	**Gammaproteobacteria (100)**	**Vibrionales (100)**	**Vibrionaceae (100)**	***Vibrio* (75)**
**OUT0434**	**Bacteria (100)**	**Actinobacteria (100)**	**Actinobacteria (100)**	**Corynebacteriales (100)**	**Corynebacteriaceae (96)**	***Corynebacterium* (94)**
**OUT0435**	**Bacteria (100)**	**Proteobacteria (100)**	**Alphaproteobacteria (100)**	**Rhodobacterales (56)**	**Rhodobacteraceae (56)**	**unclassified (<50)**
**OUT0436**	**Bacteria (100)**	**Actinobacteria (99)**	**Actinobacteria (99)**	**Pseudonocardiales (99)**	**unclassified (<50)**	**unclassified (<50)**
**OUT0437**	**Bacteria (100)**	**Actinobacteria (100)**	**Actinobacteria (100)**	**Pseudonocardiales (98)**	**Pseudonocardiaceae (84)**	***Saccharopolyspora* (65)**
**OUT0438**	**Bacteria (100)**	**Actinobacteria (100)**	**Actinobacteria (100)**	**Pseudonocardiales (100)**	**Pseudonocardiaceae (70)**	**unclassified (<50)**
**OUT0439**	**Bacteria (100)**	**Proteobacteria (100)**	**Gammaproteobacteria (100)**	**Pseudomonadales (99)**	**Pseudomonadaceae (99)**	***Pseudomonas* (91)**
**OUT0440**	**Bacteria (100)**	**Proteobacteria (100)**	**Gammaproteobacteria (100)**	**Vibrionales (100)**	**Vibrionaceae (100)**	***Vibrio* (88)**

OUT0441	Bacteria (100)	Proteobacteria (100)	Deltaproteobacteria (100)	Desulfobacterales (100)	Desulfobacteraceae (100)	*Desulfatibacillum* (100)
OUT0442	Bacteria (100)	Proteobacteria (100)	Gammaproteobacteria (100)	Pseudomonadales (98)	Moraxellaceae (98)	*Acinetobacter* (96)
OUT0443	Bacteria (100)	Actinobacteria (100)	Actinobacteria (100)	Pseudonocardiales (100)	Pseudonocardiaceae (90)	unclassified (<50)
OUT0444	Bacteria (100)	Actinobacteria (100)	Actinobacteria (100)	Pseudonocardiales (100)	unclassified (<50)	unclassified (<50)
OUT0445	Bacteria (100)	Proteobacteria (100)	Alphaproteobacteria (100)	Rhizobiales (98)	Phyllobacteriaceae (98)	*Phyllobacterium* (98)
OUT0446	Bacteria (100)	Proteobacteria (100)	Alphaproteobacteria (100)	Rhizobiales (68)	Phyllobacteriaceae (65)	*Phyllobacterium* (65)
OUT0447	Bacteria (100)	Proteobacteria (100)	Gammaproteobacteria (100)	Oceanospirillales (100)	Alcanivoraceae (100)	*Alcanivorax* (100)
OUT0448	Bacteria (100)	Proteobacteria (100)	Gammaproteobacteria (100)	Aeromonadales (70)	Aeromonadaceae (68)	unclassified (<50)
OUT0449	Bacteria (100)	Proteobacteria (100)	Gammaproteobacteria (100)	Vibrionales (83)	Vibrionaceae (83)	*Photobacterium* (52)
OUT0450	Bacteria (100)	Proteobacteria (100)	Gammaproteobacteria (100)	Pseudomonadales (100)	Moraxellaceae (100)	*Acinetobacter* (100)
OUT0451	Bacteria (99)	unclassified (<50)	unclassified (<50)	unclassified (<50)	unclassified (<50)	unclassified (<50)
OUT0452	Bacteria (100)	Actinobacteria (100)	Actinobacteria (100)	Pseudonocardiales (100)	unclassified (<50)	unclassified (<50)
OUT0453	Bacteria (100)	Proteobacteria (100)	Gammaproteobacteria (99)	Pseudomonadales (96)	Moraxellaceae (96)	*Acinetobacter* (86)
OUT0454	Bacteria (100)	Firmicutes (100)	Bacilli (100)	Lactobacillales (100)	Lactobacillaceae (100)	*Lactobacillus* (95)
OUT0455	Bacteria (100)	Proteobacteria (100)	Gammaproteobacteria (100)	Vibrionales (100)	Vibrionaceae (100)	*Allomonas* (84)
OUT0456	Bacteria (100)	Proteobacteria (100)	Gammaproteobacteria (100)	Vibrionales (88)	Vibrionaceae (88)	unclassified (<50)
OUT0457	Bacteria (100)	Actinobacteria (100)	Actinobacteria (100)	Pseudonocardiales (99)	Pseudonocardiaceae (86)	unclassified (<50)
OUT0458	Bacteria (100)	Proteobacteria (100)	Gammaproteobacteria (100)	Vibrionales (100)	Vibrionaceae (100)	*Allomonas* (79)
OUT0459	Bacteria (100)	Proteobacteria (100)	Gammaproteobacteria (100)	Enterobacteriales (100)	Enterobacteriaceae (100)	unclassified (<50)
OUT0460	Bacteria (100)	Proteobacteria (100)	Gammaproteobacteria (100)	Vibrionales (100)	Vibrionaceae (100)	*Allomonas* (50)
OUT0461	Bacteria (100)	Actinobacteria (100)	Actinobacteria (100)	Pseudonocardiales (100)	unclassified (<50)	unclassified (<50)
OUT0462	Bacteria (100)	Proteobacteria (100)	Gammaproteobacteria (100)	Vibrionales (100)	Vibrionaceae (100)	*Vibrio* (85)
OUT0463	Bacteria (100)	Proteobacteria (100)	Gammaproteobacteria (100)	Vibrionales (100)	Vibrionaceae (100)	*Vibrio* (58)
OUT0464	Bacteria (99)	Planctomycetes (56)	Planctomycetia (56)	unclassified (<50)	unclassified (<50)	unclassified (<50)

**OUT0465**	**Bacteria (100)**	**Proteobacteria (100)**	**Betaproteobacteria (100)**	**Burkholderiales (99)**	**Burkholderiaceae (99)**	***Burkholderia* (99)**
**OUT0466**	**Bacteria (100)**	**Actinobacteria (99)**	**Actinobacteria (99)**	**Pseudonocardiales (99)**	**unclassified (<50)**	**unclassified (<50)**
**OUT0467**	**Bacteria (100)**	**Proteobacteria (100)**	**Gammaproteobacteria (100)**	**Alteromonadales (100)**	**Pseudoalteromonadaceae (98)**	***Pseudoalteromonas* (98)**
**OUT0468**	**Bacteria (100)**	**Actinobacteria (100)**	**Actinobacteria (100)**	**Pseudonocardiales (100)**	**Pseudonocardiaceae (93)**	**unclassified (<50)**
**OUT0469**	**Bacteria (100)**	**Proteobacteria (100)**	**Gammaproteobacteria (100)**	**Vibrionales (96)**	**Vibrionaceae (96)**	**unclassified (<50)**
**OUT0470**	**Bacteria (100)**	**Actinobacteria (99)**	**Actinobacteria (99)**	**Pseudonocardiales (99)**	**Pseudonocardiaceae (75)**	**unclassified (<50)**
**OUT0471**	**Bacteria (100)**	**Firmicutes (100)**	**Bacilli (100)**	**Bacillalles (100)**	**Bacillales_Incertae Sedis XII (100)**	***Exiguobacterium* (100)**
**OUT0472**	**Bacteria (100)**	**Proteobacteria (100)**	**Gammaproteobacteria (100)**	**Vibrionales (98)**	**Vibrionaceae (98)**	***Vibrio* (62)**
**OUT0473**	**Bacteria (100)**	**Proteobacteria (100)**	**Gammaproteobacteria (100)**	**Pseudomonadales (96)**	**Pseudomonadaceae (96)**	***Pseudomonas* (74)**
**OUT0474**	**Bacteria (100)**	**Actinobacteria (100)**	**Actinobacteria (100)**	**Pseudonocardiales (100)**	**unclassified (<50)**	**unclassified (<50)**
**OUT0475**	**Bacteria (100)**	**Proteobacteria (100)**	**Gammaproteobacteria (100)**	**Aeromonadales (100)**	**Aeromonadaceae (59)**	**unclassified (<50)**
**OUT0476**	**Bacteria (100)**	**Firmicutes (100)**	**Bacilli (100)**	**Bacillalles (100)**	**Bacillaceae (100)**	***Oceanobacillus* (100)**
**OUT0477**	**Bacteria (100)**	**Proteobacteria (100)**	**Gammaproteobacteria (100)**	**Vibrionales (100)**	**Vibrionaceae (100)**	***Vibrio* (66)**
**OUT0478**	**Bacteria (100)**	**Bacteroidetes (100)**	**Sphingobacteria (100)**	**Sphingobacteriales (100)**	**Sphingobacteriaceae (100)**	***Sphingobacterium* (100)**
**OUT0479**	**Bacteria (100)**	**Proteobacteria (100)**	**Alphaproteobacteria (100)**	**Rhodobacterales (62)**	**Rhodobacteraceae (62)**	**unclassified (<50)**
**OUT0480**	**Bacteria (100)**	**Actinobacteria (100)**	**Actinobacteria (100)**	**Pseudonocardiales (100)**	**Pseudonocardiaceae (72)**	**unclassified (<50)**
**OUT0481**	**Bacteria (100)**	**Proteobacteria (100)**	**Alphaproteobacteria (100)**	**Rhodobacterales (93)**	**Rhodobacteraceae (93)**	***Amaricoccus* (81)**
**OUT0482**	**Bacteria (100)**	**Firmicutes (100)**	**Bacilli (100)**	**Bacillalles (100)**	**Staphylococcaceae (100)**	***Jeotgalicoccus* (90)**
**OUT0483**	**Bacteria (98)**	**Bacteroidetes (82)**	**unclassified (<50)**	**unclassified (<50)**	**unclassified (<50)**	**unclassified (<50)**
**OUT0484**	**Bacteria (100)**	**Proteobacteria (100)**	**Gammaproteobacteria (100)**	**Vibrionales (100)**	**Vibrionaceae (100)**	***Listonella* (57)**
**OUT0485**	**Bacteria (100)**	**Actinobacteria (100)**	**Actinobacteria (100)**	**Pseudonocardiales (100)**	**Pseudonocardiaceae (80)**	**unclassified (<50)**
**OUT0486**	**Bacteria (100)**	**Proteobacteria (100)**	**Alphaproteobacteria (100)**	**Rhodobacterales (74)**	**Rhodobacteraceae (74)**	**unclassified (<50)**
**OUT0487**	**Bacteria (100)**	**Proteobacteria (100)**	**Gammaproteobacteria (100)**	**Xanthomonadales (100)**	**Xanthomonadaceae (100)**	***Stenotrophomonas* (86)**
**OUT0488**	**Bacteria (100)**	**Bacteroidetes (100)**	**Bacteroidia (100)**	**Bacteroidales (100)**	**Prevotellaceae (100)**	***Prevotella* (100)**

**OUT0490**	**Bacteria (100)**	**Actinobacteria (96)**	**Actinobacteria (96)**	**Pseudonocardiales (95)**	**Pseudonocardiaceae (52)**	**unclassified (<50)**
**OUT0491**	**Bacteria (100)**	**Actinobacteria (97)**	**Actinobacteria (97)**	**Pseudonocardiales (95)**	**Pseudonocardiaceae (69)**	***Labedaea*** **(52)**
**OUT0492**	**Bacteria (100)**	**Actinobacteria (99)**	**Actinobacteria (99)**	**Pseudonocardiales (98)**	**unclassified (<50)**	**unclassified (<50)**
**OUT0494**	**Bacteria (100)**	**Firmicutes (99)**	**Bacilli (97)**	**Lactobacillales (96)**	**Streptococcaceae (94)**	***Streptococcus*** **(94)**
**OUT0495**	**Bacteria (100)**	**Proteobacteria (98)**	**Gammaproteobacteria (98**	**Enterobacteriales (95)**	**Enterobacteriaceae (95)**	**unclassified (<50)**
**OUT0497**	**Bacteria (100)**	**Actinobacteria (100)**	**Actinobacteria (100)**	**Pseudonocardiales (100)**	**Pseudonocardiaceae (66)**	**unclassified (<50)**
**OUT0498**	**Bacteria (100)**	**Actinobacteria (100)**	**Actinobacteria (100)**	**Pseudonocardiales (99)**	**Pseudonocardiaceae (88)**	**unclassified (<50)**
**OUT0499**	**Bacteria (100)**	**Proteobacteria (99)**	**Gammaproteobacteria (98**	**Pseudomonadales (92)**	**Moraxellaceae (92)**	***Acinetobacter*** **(58)**
**OUT0500**	**Bacteria (100)**	**Firmicutes (100)**	**Bacilli (96)**	**Bacillales (88)**	**Staphylococcaceae (75)**	***Staphylococcus*** **(65)**
**OUT0501**	**Bacteria (100)**	**Proteobacteria (100)**	**Gammaproteobacteria (100)**	**Vibrionales (100)**	**Vibrionaceae (100)**	***Listonella*** **(88)**
**OUT0504**	**Bacteria (100)**	**Proteobacteria (100)**	**Gammaproteobacteria (100)**	**Aeromonadales (92)**	**Aeromonadaceae (92)**	***Aeromonas*** **(89)**
**OUT0506**	**Bacteria (100)**	**Firmicutes (100)**	**Clostridia (100)**	**Clostridiales (100)**	**Clostridiales_Incertae Sedis XI**	***Anaerococcus*** **(99)**
**OUT0507**	**Bacteria (100)**	**Actinobacteria (100)**	**Actinobacteria (100)**	**Pseudonocardiales (98)**	**Pseudonocardiaceae (63)**	**unclassified (<50)**
**OUT0511**	**Bacteria (100)**	**Proteobacteria (100)**	**Gammaproteobacteria (100)**	**Vibrionales (100)**	**Vibrionaceae (100)**	***Allomonas*** **(74)**
**OUT0512**	**Bacteria (100)**	**Proteobacteria (100)**	**Gammaproteobacteria (100)**	**Vibrionales (100)**	**Vibrionaceae (100)**	***Allomonas*** **(85)**
**OUT0513**	**Bacteria (100)**	**Firmicutes (99)**	**Clostridia (99**	**Clostridiales (99)**	**Peptostreptococcaceae (80)**	**unclassified (<50)**
**OUT0514**	**Bacteria (100)**	**Proteobacteria (100)**	**Gammaproteobacteria (98**	**Alteromonadales (58)**	**unclassified (<50)**	**unclassified (<50)**
**OUT0516**	**Bacteria (100)**	**Actinobacteria (82)**	**Actinobacteria (82)**	**Pseudonocardiales (82)**	**Pseudonocardiaceae (61)**	**unclassified (<50)**
**OUT0517**	**Bacteria (100)**	**Proteobacteria (100)**	**Gammaproteobacteria (100)**	**Vibrionales (100)**	**Vibrionaceae (100)**	***Allomonas*** **(53)**
**OUT0519**	**Bacteria (100)**	**Proteobacteria (100)**	**Gammaproteobacteria (99)**	**Alteromonadales (82)**	**unclassified (<50)**	**unclassified (<50)**
**OUT0520**	**Bacteria (100)**	**Firmicutes (100)**	**Negativicutes (100)**	**Selenomonadales (100)**	**Veillonellaceae (100)**	***Dialister*** **(100)**
**OUT0521**	**Bacteria (100)**	**Actinobacteria (99)**	**Actinobacteria (99)**	**Pseudonocardiales (99)**	**Pseudonocardiaceae (64)**	**unclassified (<50)**
**OUT0523**	**Bacteria (100)**	**Actinobacteria (99)**	**Actinobacteria (99)**	**Pseudonocardiales (98)**	**Pseudonocardiaceae (79)**	**unclassified (<50)**
**OUT0524**	**Bacteria (100)**	**Actinobacteria (99)**	**Actinobacteria (99)**	**Pseudonocardiales (99)**	**Pseudonocardiaceae (84)**	**unclassified (<50)**

OUT0525	Bacteria (100)	Proteobacteria (100)	Gammaproteobacteria (100)	Vibrionales (80)	Vibrionaceae (80)	unclassified (<50)
OUT0530	Bacteria (100)	Proteobacteria (100)	Gammaproteobacteria (100)	Vibrionales (100)	Vibrionaceae (100)	*Allomonas* (82)
OUT0531	Bacteria (100)	Proteobacteria (100)	Gammaproteobacteria (100)	Vibrionales (100)	Vibrionaceae (100)	*Lucibacterium* (69)
OUT0532	Bacteria (100)	Actinobacteria (100)	Actinobacteria (100)	Pseudonocardiales (100)	Pseudonocardiaceae (75)	unclassified (<50)
OUT0533	Bacteria (100)	Actinobacteria (100)	Actinobacteria (100)	Actinomycetales (100)	Brevibacteriaceae (98)	*Brevibacterium* (98)
OUT0534	Bacteria (100)	Actinobacteria (100)	Actinobacteria (100)	Solirubrobacterales (100)	Solirubrobacteraceae (85)	*Solirubrobacter* (85)
OUT0535	Bacteria (100)	Proteobacteria (100)	Gammaproteobacteria (100)	Vibrionales (99)	Vibrionaceae (99)	*Lucibacterium* (69)
OUT0536	Bacteria (100)	Actinobacteria (99)	Actinobacteria (99)	Corynebacteriales (99)	Corynebacteriaceae (96)	*Corynebacterium* (93)
OUT0539	Bacteria (100)	Firmicutes (99)	Bacilli (97)	Bacillales (93)	Staphylococcaceae (93)	*Staphylococcus* (86)
OUT0542	Bacteria (100)	Proteobacteria (100)	Alphaproteobacteria (100)	Rhodobacterales (100)	Rhodobacteraceae (100)	unclassified (<50)
OUT0543	Bacteria (100)	Proteobacteria (93)	Gammaproteobacteria (93)	Vibrionales (92)	Vibrionaceae (92)	*Allomonas* (82)
OUT0544	Bacteria (100)	Proteobacteria (100)	Gammaproteobacteria (100)	Vibrionales (100)	Vibrionaceae (100)	*Vibrio* (82)
OUT0545	Bacteria (100)	Proteobacteria (97)	Betaproteobacteria (87	Neisseriales (82)	Neisseriaceae (82)	unclassified (<50)
OUT0546	Bacteria (100)	Proteobacteria (100)	Gammaproteobacteria (100)	Enterobacteriales (87)	Enterobacteriaceae (87)	*Escherichia/Shigella* (77)
OUT0547	Bacteria (100)	Proteobacteria (100)	Gammaproteobacteria (100)	Vibrionales (100)	Vibrionaceae (100)	*Listonella* (98)
OUT0548	Bacteria (100)	Proteobacteria (100)	Gammaproteobacteria (99)	Vibrionales (82)	Vibrionaceae (82)	unclassified (<50)
OUT0549	Bacteria (100)	Proteobacteria (100)	Gammaproteobacteria (100)	Vibrionales (99)	Vibrionaceae (99)	*Lucibacterium* (54)
OUT0550	Bacteria (100)	Proteobacteria (100)	Gammaproteobacteria (99)	Alteromonadales (97)	Pseudoalteromonadaceae (80)	*Pseudoalteromonas* (70)
OUT0551	Bacteria (100)	Proteobacteria (100)	Gammaproteobacteria (100)	Chromatiales (97)	Chromatiaceae (97)	*Rheinheimera* (97)
OUT0553	Bacteria (100)	Proteobacteria (100)	Alphaproteobacteria (100)	Rhizobiales (71)	unclassified (<50)	unclassified (<50)
OUT0555	Bacteria (100)	Actinobacteria (100)	Actinobacteria (100)	Pseudonocardiales (100)	unclassified (<50)	unclassified (<50)
OUT0556	Bacteria (100)	Proteobacteria (100)	Gammaproteobacteria (100)	Vibrionales (100)	Vibrionaceae (100)	*Aliivibrio* (100)
OUT0557	Bacteria (100)	Proteobacteria (100)	Gammaproteobacteria (100)	Vibrionales (100)	Vibrionaceae (100)	*Vibrio* (73)
OUT0558	Bacteria (100)	Firmicutes (100)	Negativicutes (100)	Selenomonadales (100)	Veillonellaceae (100)	*Negativicoccus* (100)

**OUT0560**	**Bacteria (100)**	**Proteobacteria (100)**	**Gammaproteobacteria (100)**	**Vibrionales (100)**	**Vibrionaceae (100)**	***Allomonas* (56)**
**OUT0562**	**Bacteria (100)**	**Actinobacteria (100)**	**Actinobacteria (100)**	**Pseudonocardiales (98)**	**unclassified (<50)**	**unclassified (<50)**
**OUT0565**	**Bacteria (100)**	**Actinobacteria (100)**	**Actinobacteria (100)**	**Micrococcales (99)**	**Micrococcaceae (99)**	***Micrococcus* (98)**
**OUT0566**	**Bacteria (100)**	**Actinobacteria (100)**	**Actinobacteria (100)**	**Micrococcales (100)**	**Micrococcaceae (100)**	***Micrococcus* (100)**
**OUT0567**	**Bacteria (100)**	**Actinobacteria (100)**	**Actinobacteria (100)**	**Pseudonocardiales (100)**	**Pseudonocardiaceae (75)**	**unclassified (<50)**
**OUT0568**	**Bacteria (100)**	**Proteobacteria (100)**	**Alphaproteobacteria (100)**	**Rhodospirillales (100)**	**Acetobacteraceae (100)**	**unclassified (<50)**
**OUT0570**	**Bacteria (100)**	**Proteobacteria (100)**	**Gammaproteobacteria (100)**	**Aeromonadales (89)**	**Aeromonadaceae (89)**	***Aeromonas* (87)**
**OUT0571**	**Bacteria (100)**	**Proteobacteria (100)**	**Gammaproteobacteria (100)**	**Vibrionales (100)**	**Vibrionaceae (100)**	***Listonella* (82)**
**OUT0572**	**Bacteria (100)**	**Proteobacteria (100)**	**Gammaproteobacteria (100)**	**Vibrionales (74)**	**Vibrionaceae (74)**	**unclassified (<50)**
**OUT0573**	**Bacteria (100)**	**Proteobacteria (100)**	**Gammaproteobacteria (97)**	**Pseudomonadales (96)**	**Moraxellaceae (96)**	***Acinetobacter* (94)**
**OUT0575**	**Bacteria (100)**	**Proteobacteria (100)**	**Gammaproteobacteria (100)**	**Vibrionales (100)**	**Vibrionaceae (100)**	***Allomonas* (83)**
**OUT0578**	**Bacteria (100)**	**Actinobacteria (100)**	**Actinobacteria (100)**	**Pseudonocardiales (100)**	**Pseudonocardiaceae (85)**	**unclassified (<50)**
**OUT0579**	**Bacteria (100)**	**Proteobacteria (100)**	**Alphaproteobacteria (100)**	**Rhodobacterales (100)**	**Rhodobacteraceae (100)**	***Gemmobacter* (96)**
**OUT0582**	**Bacteria (100)**	**Proteobacteria (98)**	**Alphaproteobacteria (94)**	**Rhodospirillales (88)**	**Rhodospirillaceae (71)**	**unclassified (<50)**
**OUT0583**	**Bacteria (100)**	**Proteobacteria (100)**	**Gammaproteobacteria (100)**	**Vibrionales (100)**	**Vibrionaceae (100)**	***Listonella* (71)**
**OUT0585**	**Bacteria (100)**	**Proteobacteria (99)**	**Gammaproteobacteria (99)**	**Pseudomonadales (81)**	**Pseudomonadaceae (81)**	**unclassified (<50)**
**OUT0586**	**Bacteria (100)**	**Actinobacteria (100)**	**Actinobacteria (100)**	**Pseudonocardiales (100)**	**Pseudonocardiaceae (74)**	**unclassified (<50)**
**OUT0587**	**Bacteria (100)**	**Proteobacteria (100)**	**Gammaproteobacteria (100)**	**Vibrionales (100)**	**Vibrionaceae (100)**	***Allomonas* (72)**
**OUT0589**	**Bacteria (100)**	**Proteobacteria (100)**	**Gammaproteobacteria (100)**	**Vibrionales (100)**	**Vibrionaceae (100)**	***Allomonas* (61)**
**OUT0590**	**Bacteria (100)**	**Proteobacteria (100)**	**Gammaproteobacteria (100)**	**Vibrionales (100)**	**Vibrionaceae (100)**	***Allomonas* (53)**
**OUT0591**	**Bacteria (100)**	**Proteobacteria (100)**	**Gammaproteobacteria (100)**	**Vibrionales (82)**	**Vibrionaceae (82)**	**unclassified (<50)**
**OUT0592**	**Bacteria (100)**	**Actinobacteria (98)**	**Actinobacteria (98)**	**Pseudonocardiales (98)**	**Pseudonocardiaceae (64)**	**unclassified (<50)**
**OUT0594**	**Bacteria (100)**	**Actinobacteria (99)**	**Actinobacteria (99)**	**Pseudonocardiales (99)**	**Pseudonocardiaceae (80)**	**unclassified (<50)**
**OUT0596**	**Bacteria (100)**	**Proteobacteria (100)**	**Gammaproteobacteria (100)**	**Vibrionales (100)**	**Vibrionaceae (100)**	***Allomonas* (72)**

**OUT0597**	**Bacteria (100)**	**Proteobacteria (100)**	**Gammaproteobacteria (100)**	**Vibrionales (100)**	**Vibrionaceae (100)**	***Allomonas* (78)**
**OUT0598**	**Bacteria (100)**	**Proteobacteria (100)**	**Gammaproteobacteria (100)**	**Vibrionales (100)**	**Vibrionaceae (100)**	***Listonella* (70)**
**OUT0599**	**Bacteria (100)**	**Proteobacteria (100)**	**Gammaproteobacteria (100)**	**Vibrionales (99)**	**Vibrionaceae (99)**	***Lucibacterium* (67)**
**OUT0600**	**Bacteria (100)**	**Proteobacteria (100)**	**Gammaproteobacteria (100)**	**Vibrionales (100)**	**Vibrionaceae (100)**	***Listonella* (89)**
**OUT0601**	**Bacteria (100)**	**Proteobacteria (100)**	**Gammaproteobacteria (100)**	**Vibrionales (100)**	**Vibrionaceae (100)**	***Allomonas* (90)**
**OUT0602**	**Bacteria (100)**	**Firmicutes (100)**	**Bacilli (100)**	**Lactobacillales (98)**	**Enterococcaceae (74)**	**unclassified (<50)**
**OUT0603**	**Bacteria (100)**	**Firmicutes (99)**	**Clostridia (99**	**Clostridiales (99)**	**Clostridiales_Incertae Sedis XI (99)**	***Anaerococcus* (99)**
**OUT0606**	**Bacteria (100)**	**Proteobacteria (100)**	**Gammaproteobacteria (100)**	**Pseudomonadales (100)**	**Moraxellaceae (100)**	***Acinetobacter* (99)**
**OUT0607**	**Bacteria (100)**	**Proteobacteria (100)**	**Gammaproteobacteria (100)**	**Vibrionales (88)**	**Vibrionaceae (88)**	**unclassified (<50)**
**OUT0609**	**Bacteria (100)**	**Actinobacteria (80)**	**Actinobacteria (80)**	**unclassified (<50)**	**unclassified (<50)**	**unclassified (<50)**
**OUT0610**	**Bacteria (100)**	**Proteobacteria (100)**	**Gammaproteobacteria (100)**	**Vibrionales (100)**	**Vibrionaceae (100)**	***Allomonas* (60)**
**OUT0611**	**Bacteria (100)**	**Proteobacteria (100)**	**Gammaproteobacteria (100)**	**Vibrionales (100)**	**Vibrionaceae (100)**	***Listonella* (64)**
**OUT0612**	**Bacteria (100)**	**Proteobacteria (100)**	**Gammaproteobacteria (100)**	**Pseudomonadales (100)**	**Pseudomonadaceae (100)**	***Pseudomonas* (84)**
**OUT0614**	**Bacteria (100)**	**Proteobacteria (100)**	**Gammaproteobacteria (100)**	**Vibrionales (100)**	**Vibrionaceae (100)**	***Vibrio* (51)**
**OUT0615**	**Bacteria (100)**	**Actinobacteria (100)**	**Actinobacteria (100)**	**Pseudonocardiales (100)**	**Pseudonocardiaceae (92)**	**unclassified (<50)**
**OUT0616**	**Bacteria (100)**	**Actinobacteria (100)**	**Actinobacteria (100)**	**Pseudonocardiales (100)**	**unclassified (<50)**	**unclassified (<50)**
**OUT0617**	**Bacteria (100)**	**Proteobacteria (99)**	**Gammaproteobacteria (99)**	**Oceanospirillales (84)**	**Halomonadaceae (80)**	***Halomonas* (52)**
**OUT0621**	**Bacteria (100)**	**Proteobacteria (100)**	**Gammaproteobacteria (100)**	**Aeromonadales (100)**	**Aeromonadaceae (100)**	***Aeromonas* (100)**
**OUT0622**	**Bacteria (100)**	**Proteobacteria (100)**	**Gammaproteobacteria (100)**	**Vibrionales (100)**	**Vibrionaceae (100)**	***Listonella* (93)**
**OUT0623**	**Bacteria (100)**	**Proteobacteria (100)**	**Gammaproteobacteria (100)**	**Vibrionales (100)**	**Vibrionaceae (100)**	***Vibrio* (61)**
**OUT0624**	**Bacteria (100)**	**Actinobacteria (100)**	**Actinobacteria (100)**	**Pseudonocardiales (98)**	**Pseudonocardiaceae (72)**	**unclassified (<50)**
**OUT0625**	**Bacteria (100)**	**Actinobacteria (98)**	**Actinobacteria (98)**	**Pseudonocardiales (98)**	**unclassified (<50)**	**unclassified (<50)**
**OUT0626**	**Bacteria (100)**	**Actinobacteria (100)**	**Actinobacteria (100)**	**Pseudonocardiales (98)**	**Pseudonocardiaceae (97)**	**unclassified (<50)**
**OUT0627**	**Bacteria (100)**	**Proteobacteria (100)**	**Gammaproteobacteria (100)**	**Vibrionales (100)**	**Vibrionaceae (100)**	***Listonella* (80)**

**OUT0628**	**Bacteria (100)**	**Proteobacteria (100)**	**Gammaproteobacteria (100)**	**Vibrionales (100)**	**Vibrionaceae (100)**	***Lucibacterium* (75)**
**OUT0629**	**Bacteria (100)**	**Actinobacteria (100)**	**Actinobacteria (100)**	**Pseudonocardiales (96)**	**Pseudonocardiaceae (70)**	**unclassified (<50)**
**OUT0630**	**Bacteria (100)**	**Actinobacteria (100)**	**Actinobacteria (100)**	**Pseudonocardiales (100)**	**Pseudonocardiaceae (97)**	**unclassified (<50)**
**OUT0633**	**Bacteria (100)**	**Proteobacteria (100)**	**Gammaproteobacteria (100)**	**Vibrionales (100)**	**Vibrionaceae (100)**	***Listonella* (51)**
**OUT0635**	**Bacteria (100)**	**Proteobacteria (100)**	**Gammaproteobacteria (100)**	**Vibrionales (99)**	**Vibrionaceae (99)**	***Allomonas* (69)**
**OUT0636**	**Bacteria (100)**	**unclassified (<50)**	**unclassified (<50)**	**unclassified (<50)**	**unclassified (<50)**	**unclassified (<50)**
**OUT0639**	**Bacteria (100)**	**Proteobacteria (100)**	**Gammaproteobacteria (100)**	**Vibrionales (100)**	**Vibrionaceae (100)**	***Lucibacterium* (61)**
**OUT0640**	**Bacteria (100)**	**Proteobacteria (100)**	**Alphaproteobacteria (100)**	**Sphingomonadales (100)**	**Sphingomonadaceae (100)**	***Sphingomonas* (100)**
**OUT0641**	**Bacteria (100)**	**Actinobacteria (100)**	**Actinobacteria (100)**	**Pseudonocardiales (100)**	**Pseudonocardiaceae (72)**	**unclassified (<50)**
**OUT0644**	**Bacteria (100)**	**Actinobacteria (99)**	**Actinobacteria (99)**	**Pseudonocardiales (98)**	**Pseudonocardiaceae (72)**	**unclassified (<50)**
**OUT0646**	**Bacteria (100)**	**Actinobacteria (100)**	**Actinobacteria (100)**	**Pseudonocardiales (100)**	**Pseudonocardiaceae (74)**	**unclassified (<50)**
**OUT0648**	**Bacteria (100)**	**Actinobacteria (99)**	**Actinobacteria (99)**	**Micrococcales (99)**	**Intrasporangiaceae (97)**	**unclassified (<50)**
**OUT0649**	**Bacteria (100)**	**Proteobacteria (100)**	**Gammaproteobacteria (100)**	**Chromatiales (100)**	**Chromatiaceae (100)**	***Rheinheimera* (100)**
**OUT0650**	**Bacteria (100)**	**Proteobacteria (100)**	**Gammaproteobacteria (100)**	**Vibrionales (100)**	**Vibrionaceae (100)**	***Lucibacterium* (64)**
**OUT0652**	**Bacteria (100)**	**Proteobacteria (100)**	**Gammaproteobacteria (100)**	**Aeromonadales (73)**	**Aeromonadaceae (73)**	***Aeromonas* (62)**
**OUT0654**	**Bacteria (100)**	**Proteobacteria (100)**	**Gammaproteobacteria (100)**	**Vibrionales (95)**	**Vibrionaceae (95)**	***Allomonas* (64)**
**OUT0655**	**Bacteria (100)**	**Proteobacteria (100)**	**Gammaproteobacteria (100)**	**Alteromonadales (100)**	**Alteromonadaceae (100)**	***Salinimonas* (68)**
**OUT0656**	**Bacteria (100)**	**Proteobacteria (100)**	**Gammaproteobacteria (100)**	**Vibrionales (100)**	**Vibrionaceae (100)**	***Allomonas* (79)**
**OUT0657**	**Bacteria (100)**	**Proteobacteria (100)**	**Gammaproteobacteria (99)**	**Xanthomonadales (98)**	**Xanthomonadaceae (98)**	***Luteimonas* (84)**
**OUT0658**	**Bacteria (100)**	**Actinobacteria (100)**	**Actinobacteria (100)**	**Pseudonocardiales (100)**	**Pseudonocardiaceae (85)**	**unclassified (<50)**
**OUT0659**	**Bacteria (100)**	**Actinobacteria (87)**	**Actinobacteria (87)**	**Pseudonocardiales (87)**	**unclassified (<50)**	**unclassified (<50)**
**OUT0661**	**Bacteria (100)**	**Actinobacteria (100)**	**Actinobacteria (100)**	**Micrococcales (100)**	**Dermabacteraceae (95)**	***Dermabacter* (73)**
**OUT0662**	**Bacteria (100)**	**Actinobacteria (100)**	**Actinobacteria (100)**	**Pseudonocardiales (99)**	**Pseudonocardiaceae (69)**	**unclassified (<50)**
**OUT0664**	**Bacteria (100)**	**Actinobacteria (98)**	**Actinobacteria (98)**	**Pseudonocardiales (98)**	**Pseudonocardiaceae (90)**	**unclassified (<50)**

OUT0665	Bacteria (100)	Proteobacteria (100)	Gammaproteobacteria (100)	Vibrionales (100)	Vibrionaceae (100)	*Allomonas* (72)
OUT0666	Bacteria (100)	Actinobacteria (98)	Actinobacteria (98)	Pseudonocardiales (98)	Pseudonocardiaceae (66)	unclassified (<50)
OUT0667	Bacteria (100)	Actinobacteria (100)	Actinobacteria (100)	Pseudonocardiales (100)	Pseudonocardiaceae (100)	unclassified (<50)
OUT0668	Bacteria (100)	Proteobacteria (100)	Gammaproteobacteria (99)	Pseudomonadales (99)	Moraxellaceae (99)	*Psychrobacter* (89)
OUT0669	Bacteria (100)	Actinobacteria (100)	Actinobacteria (100)	Pseudonocardiales (100)	Pseudonocardiaceae (71)	unclassified (<50)
OUT0670	Bacteria (100)	Actinobacteria (100)	Actinobacteria (100)	Pseudonocardiales (100)	Pseudonocardiaceae (72)	unclassified (<50)
OUT0671	Bacteria (100)	Proteobacteria (100)	Gammaproteobacteria (100)	Aeromonadales (100)	Aeromonadaceae (100)	*Alteromonas* (70)
OUT0672	Bacteria (100)	Proteobacteria (100)	Gammaproteobacteria (100)	Enterobacteriales (99)	Enterobacteriaceae (99)	unclassified (<50)
OUT0673	Bacteria (100)	Actinobacteria (100)	Actinobacteria (100)	Pseudonocardiales (100)	Pseudonocardiaceae (72)	unclassified (<50)
OUT0674	Bacteria (100)	Proteobacteria (100)	Alphaproteobacteria (100)	Rhodobacterales (84)	Rhodobacteraceae (84)	unclassified (<50)
OUT0675	Bacteria (100)	Proteobacteria (100)	Gammaproteobacteria (100)	Vibrionales (100)	Vibrionaceae (100)	*Allomonas* (83)
OUT0676	Bacteria (100)	Proteobacteria (100)	Gammaproteobacteria (100)	Pasteurellales (100)	Pasteurellaceae (100)	unclassified (<50)
OUT0677	Bacteria (100)	Actinobacteria (100)	Actinobacteria (100)	Pseudonocardiales (98)	Pseudonocardiaceae (80)	unclassified (<50)
OUT0678	Bacteria (100)	Proteobacteria (98)	Alphaproteobacteria (98)	Rhodospirillales (96)	Rhodospirillaceae (96)	*Pelagibius* (69)
OUT0679	Bacteria (100)	Proteobacteria (98)	Gammaproteobacteria (98)	Pseudomonadales (98)	Moraxellaceae (98)	*Acinetobacter* (91)
OUT0680	Bacteria (100)	Proteobacteria (100)	Gammaproteobacteria (100)	Pseudomonadales (100)	Moraxellaceae (100)	*Acinetobacter* (98)
OUT0681	Bacteria (100)	Proteobacteria (100)	Gammaproteobacteria (100)	Aeromonadales (79)	Aeromonadaceae (78)	*Aeromonas* (77)
OUT0682	Bacteria (100)	Proteobacteria (100)	Gammaproteobacteria (100)	Vibrionales (100)	Vibrionaceae (100)	*Allomonas* (86)
OUT0683	Bacteria (100)	Proteobacteria (100)	Gammaproteobacteria (100)	Alteromonadales (100)	Alteromonadaceae (100)	unclassified (<50)
OUT0684	Bacteria (100)	Proteobacteria (99)	Gammaproteobacteria (99)	Vibrionales (99)	Vibrionaceae (99)	*Allomonas* (88)
OUT0686	Bacteria (100)	Proteobacteria (100)	Gammaproteobacteria (100)	Alteromonadales (100)	Pseudoalteromonadaceae (97)	*Pseudoalteromonas* (94)
OUT0687	Bacteria (100)	Proteobacteria (100)	Alphaproteobacteria (100)	Rhodobacterales (100)	Rhodobacteraceae (100)	*Paracoccus* (82)
OUT0689	Bacteria (100)	Actinobacteria (100)	Actinobacteria (100)	Pseudonocardiales (100)	Pseudonocardiaceae (62)	unclassified (<50)
OUT0690	Bacteria (100)	Proteobacteria (100)	Gammaproteobacteria (100)	Pseudomonadales (84)	Pseudomonadaceae (82)	unclassified (<50)

OUT0691	Bacteria (100)	Proteobacteria (100)	Gammaproteobacteria (100)	Vibrionales (100)	Vibrionaceae (100)	*Allomonas* (92)
OUT0693	Bacteria (100)	Actinobacteria (100)	Actinobacteria (100)	Micrococcales (100)	Micrococcaceae (100)	*Rothia* (100)
OUT0694	Bacteria (100)	Actinobacteria (100)	Actinobacteria (100)	Actinomycetales (99)	unclassified (<50)	unclassified (<50)
OUT0695	Bacteria (100)	Proteobacteria (100)	Gammaproteobacteria (99)	Enterobacteriales (99)	Enterobacteriaceae (99)	unclassified (<50)
OUT0696	Bacteria (100)	Actinobacteria (100)	Actinobacteria (100)	Pseudonocardiales (99)	Pseudonocardiaceae (77)	unclassified (<50)
OUT0697	Bacteria (100)	Actinobacteria (98)	Actinobacteria (98)	Pseudonocardiales (98)	Pseudonocardiaceae (70)	unclassified (<50)
OUT0699	Bacteria	Actinobacteria (98)	Actinobacteria (98)	Pseudonocardiales (98)	Pseudonocardiaceae (59)	unclassified (<50)
OUT0700	Bacteria (100)	Proteobacteria (100)	Gammaproteobacteria (100)	Vibrionales (98)	Vibrionaceae (98)	*Salinivibrio* (54)
OUT0701	Bacteria (100)	Proteobacteria (100)	Gammaproteobacteria (100)	Pseudomonadales (99)	Pseudomonadaceae (99)	*Pseudomonas* (78)
OUT0702	Bacteria (100)	Actinobacteria (100)	Actinobacteria (100)	Actinomycetales (99)	unclassified (<50)	unclassified (<50)
OUT0703	Bacteria (100)	Proteobacteria (100)	Gammaproteobacteria (100)	Vibrionales (100)	Vibrionaceae (100)	*Allomonas* (70)
OUT0704	Bacteria (100)	Actinobacteria (99)	Actinobacteria (99)	Pseudonocardiales (97)	Pseudonocardiaceae (65)	unclassified (<50)
OUT0705	Bacteria (100)	Actinobacteria (100)	Actinobacteria (100)	Pseudonocardiales (98)	Pseudonocardiaceae (79)	unclassified (<50)
OUT0708	Bacteria (100)	Proteobacteria (100)	Gammaproteobacteria (100)	Vibrionales (100)	Vibrionaceae (100)	*Allomonas* (84)
OUT0709	Bacteria (100)	Proteobacteria (100)	Gammaproteobacteria (100)	Pseudomonadales (66)	Moraxellaceae (66)	*Acinetobacter* (64)
OUT0710	Bacteria (100)	Firmicutes (100)	Bacilli (100)	Lactobacillales (100)	Carnobacteriaceae (100)	*Dolosigranulum* (100)
OUT0711	Bacteria (100)	Actinobacteria (100)	Actinobacteria (100)	Actinomycetales (100)	unclassified (<50)	unclassified (<50)
OUT0712	Bacteria (100)	Proteobacteria (100)	Alphaproteobacteria (100)	Sphingomonadales (100)	Erythrobacteraceae (99)	*Erythrobacter* (98)
OUT0714	Bacteria (100)	Actinobacteria (100)	Actinobacteria (100)	Pseudonocardiales (98)	Pseudonocardiaceae (80)	unclassified (<50)
OUT0717	Bacteria (100)	Proteobacteria (100)	Gammaproteobacteria (100)	Vibrionales (100)	Vibrionaceae (100)	*Allomonas* (76)
OUT0719	Bacteria (100)	Firmicutes (100)	Clostridia (100)	Clostridiales (100)	Clostridiales_Incertae Sedis XI	*Anaerococcus* (99)
OUT0720	Bacteria (100)	Proteobacteria (100)	Gammaproteobacteria (100)	Vibrionales (100)	Vibrionaceae (100)	*Allomonas* (57)
OUT0721	Bacteria (100)	Proteobacteria (99)	Gammaproteobacteria (99)	Vibrionales (87)	Vibrionaceae (87)	*Allomonas* (62)
OUT0722	Bacteria (100)	Proteobacteria (100)	Gammaproteobacteria (100)	Pasteurellales (99)	Pasteurellaceae (99)	unclassified (<50)

**OUT0723**	**Bacteria (100)**	**Proteobacteria (100)**	**Gammaproteobacteria (100)**	**Vibrionales (100)**	**Vibrionaceae (100)**	***Allomonas* (87)**
**OUT0724**	**Bacteria (100)**	**Actinobacteria (99)**	**Actinobacteria (99)**	**Acidimicrobiales (84)**	**Acidimicrobiaceae (74)**	**unclassified (<50)**
**OUT0725**	**Bacteria (100)**	**Actinobacteria (100)**	**Actinobacteria (100)**	**Micrococcales (100)**	**Micrococcaceae (100)**	***Micrococcus* (97)**
**OUT0726**	**Bacteria (100)**	**Proteobacteria (100)**	**Gammaproteobacteria (100)**	**Alteromonadales (100)**	**Alteromonadaceae (100)**	***Salinimonas* (58)**
**OUT0728**	**Bacteria (100)**	**Proteobacteria (100)**	**Gammaproteobacteria (99)**	**Pseudomonadales (87)**	**Pseudomonadaceae (87)**	**unclassified (<50)**
**OUT0730**	**Bacteria (100)**	**Proteobacteria (100)**	**Gammaproteobacteria (100)**	**Vibrionales (90)**	**Vibrionaceae (90)**	***Salinivibrio* (50)**
**OUT0731**	**Bacteria (100)**	**Proteobacteria (100)**	**Gammaproteobacteria (100)**	**Vibrionales (100)**	**Vibrionaceae (100)**	***Allomonas* (79)**
**OUT0732**	**Bacteria (100)**	**Proteobacteria (100)**	**Gammaproteobacteria (100)**	**Aeromonadales (99)**	**Aeromonadaceae (99)**	***Aeromonas* (99)**
**OUT0733**	**Bacteria (100)**	**Bacteroidetes (100)**	**Bacteroidia (100)**	**Bacteroidales (100)**	**Prevotellaceae (100)**	***Prevotella* (83)**
**OUT0735**	**Bacteria (100)**	**Proteobacteria (100)**	**Gammaproteobacteria (100)**	**Enterobacteriales (100)**	**Enterobacteriaceae (100)**	***Escherichia/Shigella* (89)**
**OUT0736**	**Bacteria (100)**	**Proteobacteria (100)**	**Gammaproteobacteria (100)**	**Vibrionales (100)**	**Vibrionaceae (100)**	***Allomonas* (62)**
**OTU0737**	**Bacteria (100)**	**Proteobacteria (100)**	**Gammaproteobacteria (100)**	**Alteromonadales (100)**	**Idiomarinaceae (100)**	***Idiomarina* (100)**
**OTU0738**	**Bacteria (100)**	**Proteobacteria (100)**	**Gammaproteobacteria (99)**	**Pseudomonadales (99)**	**Moraxellaceae (99)**	***Acinetobacter* (99)**
**OTU0739**	**Bacteria (100)**	**Proteobacteria (100)**	**Gammaproteobacteria (100)**	**Vibrionales (100)**	**Vibrionaceae (100)**	***Allomonas* (80)**
**OTU0741**	**Bacteria (100)**	**Proteobacteria (99)**	**Gammaproteobacteria (98**	**Pseudomonadales (69)**	**Pseudomonadaceae (68)**	***Pseudomonas* (60)**
**OTU0742**	**Bacteria (100)**	**Proteobacteria (100)**	**Gammaproteobacteria (100)**	**Vibrionales (100)**	**Vibrionaceae (100)**	***Allomonas* (70)**
**OTU0743**	**Bacteria (100)**	**Proteobacteria (100)**	**Gammaproteobacteria (100)**	**Vibrionales (97)**	**Vibrionaceae (97)**	**unclassified (<50)**
**OTU0744**	**Bacteria (100)**	**Actinobacteria (100)**	**Actinobacteria (100)**	**Pseudonocardiales (98)**	**Pseudonocardiaceae (78)**	**unclassified (<50)**
**OTU0745**	**Bacteria (100)**	**Proteobacteria (100)**	**Gammaproteobacteria (100)**	**Enterobacteriales (100)**	**Enterobacteriaceae (100)**	**unclassified (<50)**
**OTU0746**	**Bacteria (100)**	**Proteobacteria (100)**	**Gammaproteobacteria (100)**	**Vibrionales (100)**	**Vibrionaceae (100)**	***Listonella* (83)**
**OTU0747**	**Bacteria (100)**	**Actinobacteria (100)**	**Actinobacteria (100)**	**Pseudonocardiales (100)**	**Pseudonocardiaceae (70)**	**unclassified (<50)**
**OTU0748**	**Bacteria (100)**	**Proteobacteria (99)**	**Gammaproteobacteria (99)**	**Pseudomonadales (99)**	**Moraxellaceae (99)**	***Acinetobacter* (95)**
**OTU0750**	**Bacteria (100)**	**Actinobacteria (100)**	**Actinobacteria (100)**	**Pseudonocardiales (99)**	**Pseudonocardiaceae (90)**	**unclassified (<50)**

OTU0752	Bacteria (100)	Firmicutes (97)	Bacilli (96)	Lactobacillales (95)	Streptococcaceae (94)	*Streptococcus* (92)
OTU0754	Bacteria (100)	Actinobacteria (100)	Actinobacteria (100)	Bifidobacteriales (100)	Bifidobacteriaceae (100)	*Scardovia* (100)
OTU0757	Bacteria (100)	Proteobacteria (100)	Gammaproteobacteria (100)	Vibrionales (100)	Vibrionaceae (100)	*Listonella* (93)
OTU0759	Bacteria (100)	Proteobacteria (100)	Gammaproteobacteria (100)	Enterobacteriales (100)	Enterobacteriaceae (100)	*Proteus* (99)
OTU0760	Bacteria (100)	Firmicutes (100)	Bacilli (100)	Bacillalles (100)	Staphylococcaceae (99)	*Staphylococcus* (95)
OTU0762	Bacteria (100)	Proteobacteria (100)	Gammaproteobacteria (100)	Vibrionales (99)	Vibrionaceae (99)	*Allomonas* (53)
OTU0763	Bacteria (100)	Proteobacteria (100)	Gammaproteobacteria (100)	Vibrionales (100)	Vibrionaceae (100)	*Allomonas* (84)
OTU0764	Bacteria (100)	Proteobacteria (100)	Gammaproteobacteria (100)	Vibrionales (100)	Vibrionaceae (100)	*Allomonas* (72)
OTU0767	Bacteria (100)	Proteobacteria (100)	Gammaproteobacteria (100)	Alteromonadales (100)	Pseudoalteromonadaceae (100)	*Pseudoalteromonas* (99)
OTU0768	Bacteria (100)	Proteobacteria (100)	Gammaproteobacteria (100)	Vibrionales (100)	Vibrionaceae (100)	*Allomonas* (53)
OTU0770	Bacteria (100)	Proteobacteria (100)	Gammaproteobacteria (100)	Vibrionales (100)	Vibrionaceae (100)	*Vibrio* (77)
OTU0771	Bacteria (100)	Proteobacteria (100)	Gammaproteobacteria (100)	Vibrionales (100)	Vibrionaceae (100)	*Listonella* (70)
OTU0772	Bacteria (100)	Firmicutes (100)	Clostridia (100)	Clostridiales (100)	Clostridiales_Incertae Sedis XI (100)	*Anaerococcus* (100)
OUT0773	Bacteria (100)	Proteobacteria (100)	Gammaproteobacteria (100)	Pseudomonadales (100)	Moraxellaceae (100)	*Acinetobacter* (98)
OUT0776	Bacteria (100)	Proteobacteria (100)	Gammaproteobacteria (99)	Vibrionales (99)	Vibrionaceae (99)	*Listonella* (87)
OUT0778	Bacteria (100)	Proteobacteria (100)	Gammaproteobacteria (100)	Vibrionales (100)	Vibrionaceae (100)	*Allomonas* (85)
OUT0780	Bacteria (100)	Actinobacteria (100)	Actinobacteria (100)	Corynebacteriales (100)	Corynebacteriaceae (100)	*Corynebacterium* (100)
OUT0781	Bacteria (100)	Proteobacteria (100)	Gammaproteobacteria (100)	Vibrionales (100)	Vibrionaceae (100)	*Allomonas* (54)
OUT0782	Bacteria (100)	Bacteroidetes (100)	Flavobacteria (100)	Flavobacteriales (100)	Flavobacteriaceae (100)	*Chryseobacterium* (99)
OUT0783	Bacteria (100)	Bacteroidetes (100)	Bacteroidia (90)	Bacteroidales (90)	Porphyromonadaceae (69)	unclassified (<50)
OUT0784	Bacteria (100)	Proteobacteria (100)	Gammaproteobacteria (100)	Alteromonadales (100)	Pseudoalteromonadaceae (98)	*Pseudoalteromonas* (98)
OUT0786	Bacteria (100)	Proteobacteria (100)	Gammaproteobacteria (100)	Vibrionales (100)	Vibrionaceae (100)	*Lucibacterium* (63)
OUT0787	Bacteria (100)	Firmicutes (90)	Bacilli (86	Lactobacillales (84)	Streptococcaceae (70)	*Streptococcus* (70)

**OUT0788**	**Bacteria (100)**	**Proteobacteria (100)**	**Gammaproteobacteria (100)**	**Enterobacteriales (100)**	**Enterobacteriaceae (100)**	**unclassified (<50)**
**OUT0789**	**Bacteria (100)**	**Actinobacteria (100)**	**Actinobacteria (100)**	**Actinomycetales (100)**	**unclassified (<50)**	**unclassified (<50)**
**OUT0790**	**Bacteria (100)**	**Proteobacteria (100)**	**Gammaproteobacteria (100)**	**Vibrionales (100)**	**Vibrionaceae (100)**	***Allomonas* (58)**
**OUT0791**	**Bacteria (100)**	**Firmicutes (100)**	**Bacilli (100)**	**Lactobacillales (93)**	**Enterococcaceae (67)**	***Enterococcus* (50)**
**OUT0792**	**Bacteria (100)**	**Proteobacteria (100)**	**Gammaproteobacteria (100)**	**Aeromonadales (73)**	**Aeromonadaceae (73)**	***Aeromonas* (67)**
**OUT0793**	**Bacteria (100)**	**Actinobacteria (100)**	**Actinobacteria (100)**	**Actinomycetales (100)**	**unclassified (<50)**	**unclassified (<50)**
**OUT0794**	**Bacteria (100)**	**Proteobacteria (100)**	**Gammaproteobacteria (100)**	**Aeromonadales (76)**	**Aeromonadaceae (76)**	***Aeromonas* (74)**
**OUT0795**	**Bacteria (100)**	**Actinobacteria (100)**	**Actinobacteria (100)**	**Pseudonocardiales (100)**	**Pseudonocardiaceae (78)**	**unclassified (<50)**
**OUT0796**	**Bacteria (100)**	**Actinobacteria (100)**	**Actinobacteria (100)**	**Pseudonocardiales (98)**	**Pseudonocardiaceae (83)**	**unclassified (<50)**
**OUT0797**	**Bacteria (100)**	**Actinobacteria (99)**	**Actinobacteria (99)**	**Actinomycetales (99)**	**unclassified (<50)**	**unclassified (<50)**
**OUT0798**	**Bacteria (100)**	**Actinobacteria (100)**	**Actinobacteria (100)**	**Actinomycetales (99)**	**unclassified (<50)**	**unclassified (<50)**
**OUT0799**	**Bacteria (100)**	**Proteobacteria (98)**	**Alphaproteobacteria (100)**	**Rhizobiales (88)**	**Rhodobiaceae (69)**	***Anderseniella* (62)**
**OUT0800**	**Bacteria (100)**	**Proteobacteria (100)**	**Betaproteobacteria (100)**	**Burkholderiales (99)**	**unclassified (<50)**	**unclassified (<50)**
**OTU0801**	**Bacteria (100)**	**Proteobacteria (100)**	**Gammaproteobacteria (99)**	**Pasteurellales (99)**	**Pasteurellaceae (99)**	**unclassified (<50)**
**OTU0802**	**Bacteria (100)**	**Proteobacteria (100)**	**Gammaproteobacteria (100)**	**Pasteurellales (100)**	**Pasteurellaceae (100)**	***Haemophilus* (64)**
**OTU0803**	**Bacteria (100)**	**Actinobacteria (100)**	**Actinobacteria (100)**	**Pseudonocardiales (100)**	**Pseudonocardiaceae (86)**	**unclassified (<50)**
**OTU0804**	**Bacteria (100)**	**Actinobacteria (100)**	**Actinobacteria (100)**	**Pseudonocardiales (100)**	**Pseudonocardiaceae (90)**	**unclassified (<50)**
**OTU0805**	**Bacteria (100)**	**Proteobacteria (99)**	**Gammaproteobacteria (98)**	**Aeromonadales (51)**	**Aeromonadaceae (51)**	**unclassified (<50)**
**OTU0806**	**Bacteria (99)**	**Actinobacteria (98)**	**Actinobacteria (98)**	**Pseudonocardiales (97)**	**Pseudonocardiaceae (77)**	**unclassified (<50)**
**OTU0807**	**Bacteria (100)**	**Proteobacteria (100)**	**Alphaproteobacteria (100)**	**Rhizobiales (100)**	**Methylobacteriaceae (89)**	***Microvirga* (89)**
**OTU0808**	**Bacteria (100)**	**Proteobacteria (100)**	**Gammaproteobacteria (100)**	**Vibrionales (98)**	**Vibrionaceae (98)**	***Allomonas* (86)**
**OTU0809**	**Bacteria (100)**	**Actinobacteria (100)**	**Actinobacteria (100)**	**Actinomycetales (100)**	**unclassified (<50)**	**unclassified (<50)**
**OTU0810**	**Bacteria (100)**	**Proteobacteria (99)**	**Alphaproteobacteria (99)**	**Rhizobiales (99)**	**Phyllobacteriaceae (99)**	***Phyllobacterium* (99)**

**OTU0811**	**Bacteria (100)**	**Proteobacteria (100)**	**Betaproteobacteria (100)**	**Burkholderiales (100)**	**unclassified (<50)**	**unclassified (<50)**
**OTU0812**	**Bacteria (100)**	**Actinobacteria (100)**	**Actinobacteria (100)**	**Pseudonocardiales (100)**	**Pseudonocardiaceae (74)**	**unclassified (<50)**
**OTU0813**	**Bacteria (100)**	**Actinobacteria (100)**	**Actinobacteria (100)**	**Pseudonocardiales (99)**	**Pseudonocardiaceae (87)**	**unclassified (<50)**
**OTU0814**	**Bacteria (100)**	**Actinobacteria (100)**	**Actinobacteria (100)**	**Bifidobacteriales (100)**	**Bifidobacteriaceae (100)**	***Gardnerella* (99)**
**OTU0815**	**Bacteria (100)**	**Actinobacteria (100)**	**Actinobacteria (100)**	**Bifidobacteriales (100)**	**Bifidobacteriaceae (100)**	***Gardnerella* (99)**
**OTU0816**	**Bacteria (100)**	**Actinobacteria (100)**	**Actinobacteria (100)**	**Actinomycetales (99)**	**unclassified (<50)**	**unclassified (<50)**
**OTU0817**	**Bacteria (100)**	**Proteobacteria (100)**	**Gammaproteobacteria (100)**	**Vibrionales (95)**	**Vibrionaceae (95)**	***Photobacterium* (88)**
**OTU0818**	**Bacteria (100)**	**Proteobacteria (100)**	**Betaproteobacteria (100)**	**Burkholderiales (100)**	**Comamonadaceae (86)**	***Diaphorobacter* (53)**
**OTU0819**	**Bacteria (100)**	**Actinobacteria (100)**	**Actinobacteria (100)**	**Pseudonocardiales (100)**	**Pseudonocardiaceae (90)**	**unclassified (<50)**
**OTU0820**	**Bacteria (100)**	**Actinobacteria (98)**	**Actinobacteria (98)**	**Pseudonocardiales (96)**	**Pseudonocardiaceae (82)**	***Umezawaea* (67)**
**OTU0821**	**Bacteria (100)**	**Proteobacteria (100)**	**Gammaproteobacteria (100)**	**Enterobacteriales (100)**	**Enterobacteriaceae (100)**	***Escherichia/Shigella* (98)**
**OTU0822**	**Bacteria (100)**	**Planctomycets (90)**	**Planctomycetia (90)**	**Planctomycetales (90)**	**Planctomycetaceae (90)**	**unclassified (<50)**
**OUT0823**	**Bacteria**	**Actinobacteria (84)**	**Actinobacteria (84)**	**Pseudonocardiales (78)**	**Pseudonocardiaceae (50)**	**unclassified (<50)**
**OUT0824**	**Bacteria (100)**	**Proteobacteria (100)**	**Gammaproteobacteria (100)**	**Vibrionales (100)**	**Vibrionaceae (100)**	***Listonella* (70)**
**OTU0825**	**Bacteria (100)**	**Proteobacteria (100)**	**Betaproteobacteria (100)**	**Burkholderiales (100)**	**Oxalobacteraceae (100)**	***Massilia* (58)**
**OTU0826**	**Bacteria (100)**	**Actinobacteria (100)**	**Actinobacteria (100)**	**Pseudonocardiales (100)**	**Pseudonocardiaceae (96)**	**unclassified (<50)**
**OTU0827**	**Bacteria (100)**	**Proteobacteria (100)**	**Gammaproteobacteria (100)**	**Pseudomonadales (98)**	**Moraxellaceae (98)**	***Acinetobacter* (95)**
**OTU0828**	**Bacteria (100)**	**Firmicutes (100)**	**Bacilli (100)**	**Lactobacillales (100)**	**Streptococcaceae (100)**	***Streptococcus* (100)**
**OTU0829**	**Bacteria (100)**	**Proteobacteria (100)**	**Gammaproteobacteria (100)**	**Pseudomonadales (99)**	**Pseudomonadaceae (88)**	***Pseudomonas* (83)**
**OTU0830**	**Bacteria (100)**	**Proteobacteria (100)**	**Alphaproteobacteria (100)**	**Rhizobiales (100)**	**Methylobacteriaceae (98)**	***Methylobacterium* (98)**
**OTU0831**	**Bacteria (100)**	**Firmicutes (100)**	**Clostridia (100)**	**Clostridiales (100)**	**Clostridiales_Incertae Sedis XI (100)**	***Anaerococcus* (100)**
**OTU0832**	**Bacteria (100)**	**Proteobacteria (100)**	**Gammaproteobacteria (100)**	**Vibrionales (100)**	**Vibrionaceae (100)**	***Allomonas* (87)**
**OTU0833**	**Bacteria (100)**	**Actinobacteria (100)**	**Actinobacteria (100)**	**Pseudonocardiales (100)**	**Pseudonocardiaceae (76)**	**unclassified (<50)**

OTU0834	Bacteria (100)	Proteobacteria (100)	Gammaproteobacteria (100)	Pasteurellales (100)	Pasteurellaceae (100)	*Aggregatibacter* (55)
OTU0835	Bacteria (100)	Proteobacteria (100)	Gammaproteobacteria (100)	Aeromonadales (88)	Aeromonadaceae (88)	*Aeromonas* (81)
OTU0836	Bacteria (100)	Proteobacteria (100)	Gammaproteobacteria (100)	Vibrionales (100)	Vibrionaceae (100)	*Listonella* (70)
OTU0837	Bacteria (100)	Proteobacteria (100)	Gammaproteobacteria (100)	Enterobacteriales (100)	Enterobacteriaceae (100)	*Cronobacter* (53)
OTU0838	Bacteria (100)	Proteobacteria (100)	Gammaproteobacteria (100)	Vibrionales (100)	Vibrionaceae (100)	*Allomonas* (92)
OTU0839	Bacteria (100)	Proteobacteria (100)	Gammaproteobacteria (100)	Vibrionales (100)	Vibrionaceae (100)	*Lucibacterium* (50)
OTU0840	Bacteria (100)	Fusobacteria (100)	Fusobacteriia (100)	Fusobacteriales (100)	Fusobacteriaceae (100)	*Fusobacterium* (100)
OTU0841	Bacteria (100)	Proteobacteria (100)	Gammaproteobacteria (100)	Alteromonadales (100)	Pseudoalteromonadaceae (97)	*Pseudoalteromonas* (96)
OTU0842	Bacteria (100)	Actinobacteria (100)	Actinobacteria (100)	Pseudonocardiales (100)	Pseudonocardiaceae (85)	unclassified (<50)
OTU0843	Bacteria (100)	Proteobacteria (100)	Alphaproteobacteria (100)	Rhodobacterales (98)	Rhodobacteraceae (98)	*Paracoccus* (67)
OTU0844	Bacteria (100)	Firmicutes (97)	Bacilli (84)	Bacillales (70)	Staphylococcaceae (64)	unclassified (<50)
OTU0845	Bacteria (100)	Firmicutes (99)	Bacilli (97)	Bacillales (89)	Bacillaceae2 (65)	unclassified (<50)
OTU0846	Bacteria (100)	Fusobacteria (100)	Fusobacteriia (100)	Fusobacteriales (100)	Fusobacteriaceae (100)	*Cetobacterium* (96)
OTU0847	Bacteria (100)	Proteobacteria (100)	Alphaproteobacteria (100)	unclassified (<50)	unclassified (<50)	unclassified (<50)
OTU0848	Bacteria (100)	Actinobacteria (100)	Actinobacteria (100)	Pseudonocardiales (100)	Pseudonocardiaceae (84)	unclassified (<50)
OUT0849	Bacteria (100)	Actinobacteria (100)	Actinobacteria (100)	Pseudonocardiales (100)	Pseudonocardiaceae (85)	unclassified (<50)
OUT0850	Bacteria (100)	Proteobacteria (100)	Gammaproteobacteria (100)	Vibrionales (100)	Vibrionaceae (100)	*Listonella* (81)
OUT0851	Bacteria (100)	Actinobacteria (100)	Actinobacteria (100)	Actinomycetales (100)	unclassified (<50)	unclassified (<50)
OUT0852	Bacteria (100)	Actinobacteria (100)	Actinobacteria (100)	Actinomycetales (100)	unclassified (<50)	unclassified (<50)
OUT0853	Bacteria (100)	Proteobacteria (100)	Gammaproteobacteria (100)	Vibrionales (100)	Vibrionaceae (100)	*Vibrio* (68)
OUT0854	Bacteria (100)	Actinobacteria (99)	Actinobacteria (99)	Pseudonocardiales (97)	Pseudonocardiaceae (75)	unclassified (<50)
OUT0855	Bacteria (100)	Actinobacteria (100)	Actinobacteria (100)	Bifidobacteriales (100)	Bifidobacteriaceae (100)	*Scardovia* (100)
OUT0856	Bacteria (100)	Actinobacteria (100)	Actinobacteria (100)	Actinomycetales (99)	unclassified (<50)	unclassified (<50)

**OUT0857**	**Bacteria (100)**	**Actinobacteria (100)**	**Actinobacteria (100)**	**Micrococcales (100)**	**Micrococcaceae (97)**	***Rothia* (97)**
**OUT0858**	**Bacteria (100)**	**Actinobacteria (100)**	**Actinobacteria (100)**	**Pseudonocardiales (100)**	**Pseudonocardiaceae (65)**	**unclassified (<50)**
**OUT0859**	**Bacteria (100)**	**Proteobacteria (100)**	**Gammaproteobacteria (100)**	**Enterobacteriales (100)**	**Enterobacteriaceae (100)**	***Escherichia/Shigella* (81)**
**OUT0860**	**Bacteria (100)**	**Actinobacteria (99)**	**Actinobacteria (99)**	**Pseudonocardiales (99)**	**Pseudonocardiaceae (62)**	**unclassified (<50)**
**OUT0861**	**Bacteria (100)**	**Proteobacteria (100)**	**Alphaproteobacteria (100)**	**Rhizobiales (95)**	**unclassified (<50)**	**unclassified (<50)**
**OUT0862**	**Bacteria (100)**	**Proteobacteria (100)**	**Gammaproteobacteria (100)**	**Vibrionales (100)**	**Vibrionaceae (100)**	***Listonella* (73)**
**OUT0863**	**Bacteria (100)**	**Actinobacteria (98)**	**Actinobacteria (98)**	**Corynebacteriales (96)**	**Corynebacteriaceae (91)**	***Corynebacterium* (88)**
**OUT0864**	**Bacteria (100)**	**Proteobacteria (100)**	**Gammaproteobacteria (100)**	**Vibrionales (100)**	**Vibrionaceae (100)**	***Listonella* (90)**
**OUT0865**	**Bacteria (100)**	**Actinobacteria (100)**	**Actinobacteria (100)**	**Actinomycetales (100)**	**unclassified (<50)**	**unclassified (<50)**
**OUT0866**	**Bacteria (100)**	**Proteobacteria (100)**	**Gammaproteobacteria (100)**	**Aeromonadales (95)**	**Aeromonadaceae (95)**	***Aeromonas* (95)**
**OUT0867**	**Bacteria (100)**	**Proteobacteria (100)**	**Gammaproteobacteria (98)**	**Alteromonadales (77)**	**Shewanellaceae (64)**	***Shewanella* (64)**
**OUT0868**	**Bacteria (100)**	**Actinobacteria (100)**	**Actinobacteria (100)**	**Pseudonocardiales (97)**	**Pseudonocardiaceae (88)**	**unclassified (<50)**
**OUT0869**	**Bacteria (100)**	**Bacteroidetes (100)**	**Bacteroidia (100)**	**Bacteroidales (100)**	**Porphyromonadaceae (100)**	***Porphyromonas* (100)**
**OUT0870**	**Bacteria (100)**	**Actinobacteria (100)**	**Actinobacteria (100)**	**Pseudonocardiales (99)**	**Pseudonocardiaceae (87)**	**unclassified (<50)**
**OUT0871**	**Bacteria (100)**	**Proteobacteria (100)**	**Gammaproteobacteria (100)**	**Pseudomonadales (90)**	**Pseudomonadaceae (90)**	***Pseudomonas* (51)**
**OUT0872**	**Bacteria (100)**	**Actinobacteria (96)**	**Actinobacteria (96)**	**Pseudonocardiales (95)**	**Pseudonocardiaceae (75)**	***Labedaea* (55)**
**OTU0873**	**Bacteria (100)**	**Proteobacteria (100)**	**Alphaproteobacteria (99)**	**unclassified (<50)**	**unclassified (<50)**	**unclassified (<50)**
**OTU0874**	**Bacteria (100)**	**Actinobacteria (98)**	**Actinobacteria (98)**	**Pseudonocardiales (98)**	**Pseudonocardiaceae (68)**	**unclassified (<50)**
**OTU0875**	**Bacteria (100)**	**Proteobacteria (100)**	**Alphaproteobacteria (100)**	**Rhizobiales (93)**	**unclassified (<50)**	**unclassified (<50)**
**OTU0876**	**Bacteria (100)**	**Actinobacteria (100)**	**Actinobacteria (100)**	**Actinomycetales (100)**	**unclassified (<50)**	**unclassified (<50)**
**OTU0877**	**Bacteria (100)**	**Actinobacteria (100)**	**Actinobacteria (100)**	**Pseudonocardiales (100)**	**Pseudonocardiaceae (67)**	**unclassified (<50)**
**OTU0878**	**Bacteria (100)**	**Proteobacteria (100)**	**Gammaproteobacteria (100)**	**Vibrionales (100)**	**Vibrionaceae (100)**	***Allomonas* (70)**
**OTU0879**	**Bacteria (100)**	**Actinobacteria (98)**	**Actinobacteria (98)**	**Actinomycetales (96)**	**unclassified (<50)**	**unclassified (<50)**

**OTU0880**	**Bacteria (100)**	**Proteobacteria (100)**	**Gammaproteobacteria (100)**	**Vibrionales (100)**	**Vibrionaceae (100)**	***Listonella* (96)**
**OTU0881**	**Bacteria (100)**	**Proteobacteria (100)**	**Gammaproteobacteria (100)**	**Pseudomonadales (100)**	**Moraxellaceae (100)**	***Acinetobacter* (100)**
**OTU0882**	**Bacteria (100)**	**Firmicutes (100)**	**Bacilli (100)**	**Bacillales (92)**	**unclassified (<50)**	**unclassified (<50)**
**OTU0883**	**Bacteria (100)**	**Actinobacteria (100)**	**Actinobacteria (100)**	**Actinomycetales (100)**	**unclassified (<50)**	**unclassified (<50)**
**OTU0884**	**Bacteria (100)**	**Actinobacteria (100)**	**Actinobacteria (100)**	**Actinomycetales (100)**	**unclassified (<50)**	**unclassified (<50)**
**OTU0885**	**Bacteria (100)**	**Firmicutes (99)**	**Negativicutes (90)**	**Selenomonadales (90)**	**Veillonellaceae (90)**	**unclassified (<50)**
**OTU0886**	**Bacteria (100)**	**Actinobacteria (100)**	**Actinobacteria (100)**	**Pseudonocardiales (100)**	**Pseudonocardiaceae (79)**	**unclassified (<50)**
**OTU0887**	**Bacteria (100)**	**Firmicutes (100)**	**Bacilli (99)**	**Bacillales (99)**	**Bacillales_Incertae Sedis XI (97)**	***Gemella* (97)**
**OTU0888**	**Bacteria (100)**	**Proteobacteria (100)**	**Gammaproteobacteria (100)**	**Pseudomonadales (100)**	**Pseudomonadaceae (100)**	***Pseudomonas* (84)**
**OTU0888**	**Bacteria (100)**	**Proteobacteria (100)**	**Gammaproteobacteria (100)**	**Pseudomonadales (98)**	**Pseudomonadaceae (98)**	***Azotobacter* (63)**
**OTU0889**	**Bacteria (100)**	**Proteobacteria (100)**	**Alphaproteobacteria (100)**	**Rhodobacterales (90)**	**Rhodobacteraceae (90)**	**unclassified (<50)**
**OTU0889**	**Bacteria (100)**	**Proteobacteria (100)**	**Alphaproteobacteria (100)**	**Rhizobiales (55)**	**Phyllobacteriaceae (53)**	***Phyllobacterium* (53)**
**OTU0890**	**Bacteria (100)**	**Firmicutes (100)**	**Bacilli (100)**	**Bacillalles (100)**	**Planococcaceae (84)**	***Chryseomicrobium* (80)**
**OTU0891**	**Bacteria (100)**	**Actinobacteria (99)**	**Actinobacteria (99)**	**Pseudonocardiales (99)**	**Pseudonocardiaceae (58)**	**unclassified (<50)**
**OTU0892**	**Bacteria (100)**	**Proteobacteria (100)**	**Gammaproteobacteria (100)**	**Aeromonadales (85)**	**Aeromonadaceae (85)**	***Aeromonas* (81)**
**OTU0892**	**Bacteria (100)**	**Proteobacteria (100)**	**Gammaproteobacteria (100)**	**Aeromonadales (97)**	**Aeromonadaceae (97)**	***Aeromonas* (97)**
**OTU0893**	**Bacteria (100)**	**Proteobacteria (100)**	**Alphaproteobacteria (100)**	**unclassified (<50)**	**unclassified (<50)**	**unclassified (<50)**
**OUT0894**	**Bacteria (100)**	**Firmicutes (99)**	**Bacilli (99)**	**Bacillales (53)**	**unclassified (<50)**	**unclassified (<50)**
**OUT0895**	**Bacteria (100)**	**Actinobacteria (100)**	**Actinobacteria (100)**	**Pseudonocardiales (99)**	**Pseudonocardiaceae (78)**	**unclassified (<50)**
**OUT0896**	**Bacteria (100)**	**Actinobacteria (100)**	**Actinobacteria (100)**	**Pseudonocardiales (100)**	**Pseudonocardiaceae (70)**	**unclassified (<50)**
**OUT0897**	**Bacteria (100)**	**Firmicutes (100)**	**Bacilli (100)**	**Lactobacillales (92)**	**Enterococcaceae (61)**	**unclassified (<50)**
**OUT0898**	**Bacteria (100)**	**Actinobacteria (100)**	**Actinobacteria (100)**	**Actinomycetales (100)**	**Actinomycetaceae (100)**	***Actinomyces* (100)**
**OUT0899**	**Bacteria (100)**	**Actinobacteria (89)**	**Actinobacteria (89)**	**Pseudonocardiales (88)**	**Pseudonocardiaceae (75)**	***Labedaea* (54)**

OUT0900	Bacteria (100)	Actinobacteria (100)	Actinobacteria (100)	Geodermatophylales (100)	Geodermatophylaceae (98)	*Geodermatophylus* (69)
OUT0901	Bacteria (100)	Actinobacteria (100)	Actinobacteria (100)	Bifidobacteriales (100)	Bifidobacteriaceae (100)	*Gardnerella* (98)
OUT0902	Bacteria (100)	Actinobacteria (99)	Actinobacteria (99)	Pseudonocardiales (99)	Pseudonocardiaceae (80)	*Labedaea* (52)
OUT0903	Bacteria (100)	Actinobacteria (100)	Actinobacteria (100)	Pseudonocardiales (100)	Pseudonocardiaceae (89)	unclassified (<50)
OUT0904	Bacteria (100)	Proteobacteria (99)	Alphaproteobacteria (99)	Rhizobiales (59)	Phyllobacteriaceae (56)	*Phyllobacterium* (56)
OUT0905	Bacteria (100)	Actinobacteria (99)	Actinobacteria (99)	Pseudonocardiales (96)	Pseudonocardiaceae (85)	unclassified (<50)
OUT0906	Bacteria (100)	Firmicutes (100)	Bacilli (100)	Bacillales (92)	Bacillaceae 2 (55)	unclassified (<50)
OUT0907	Bacteria (100)	Proteobacteria (100)	Gammaproteobacteria (100)	Vibrionales (96)	Vibrionaceae (96)	unclassified (<50)
OUT0908	Bacteria (100)	Actinobacteria (100)	Actinobacteria (100)	Pseudonocardiales (100)	Pseudonocardiaceae (88)	unclassified (<50)
OUT0909	Bacteria (100)	Proteobacteria (100)	Gammaproteobacteria (99)	Pseudomonadales (90)	Pseudomonadaceae (90)	unclassified (<50)
OUT0910	Bacteria (100)	Firmicutes (100)	Bacilli (100)	Lactobacillales (90)	unclassified (<50)	unclassified (<50)
OUT0911	Bacteria (100)	Actinobacteria (100)	Actinobacteria (100)	Pseudonocardiales (94)	Pseudonocardiaceae (76)	unclassified (<50)
OUT0912	Bacteria (100)	Firmicutes (100)	Negativicutes (99)	Selenomonadales (99)	Veillonellaceae (99)	*Veillonella* (62)
OUT0913	Bacteria (100)	Actinobacteria (100)	Actinobacteria (100)	Pseudonocardiales (100)	Pseudonocardiaceae (85)	unclassified (<50)
OUT0914	Bacteria (100)	Proteobacteria (100)	Gammaproteobacteria (100)	Vibrionales (100)	Vibrionaceae (100)	*Allomonas* (78)
OUT0915	Bacteria (100)	Actinobacteria (100)	Actinobacteria (100)	Pseudonocardiales (100)	Pseudonocardiaceae (78)	unclassified (<50)
OUT0916	Bacteria (100)	Proteobacteria (100)	Gammaproteobacteria (99)	Pseudomonadales (94)	Pseudomonadaceae (94)	*Serpens* (60)
OUT0917	Bacteria (100)	Proteobacteria (100)	Alphaproteobacteria (100)	Rhizobiales (78)	Phyllobacteriaceae (78)	*Phyllobacterium* (76)
OTU0918	Bacteria (100)	Actinobacteria (99)	Actinobacteria (99)	Pseudonocardiales (98)	Pseudonocardiaceae (75)	unclassified (<50)
OTU0919	Bacteria (100)	Actinobacteria (100)	Actinobacteria (100)	Actinomycetales (100)	unclassified (<50)	unclassified (<50)
OTU0920	Bacteria (100)	Proteobacteria (100)	Gammaproteobacteria (100)	Vibrionales (97)	Vibrionaceae (97)	*Allomonas* (80)
OTU0921	Bacteria (100)	Actinobacteria (100)	Actinobacteria (100)	Actinomycetales (100)	unclassified (<50)	unclassified (<50)
OTU0922	Bacteria (100)	Proteobacteria (100)	Alphaproteobacteria (100)	Rhodobacterales (99)	Rhodobacteraceae (99)	unclassified (<50)

**OTU0923**	**Bacteria (100)**	**Actinobacteria (100)**	**Actinobacteria (100)**	**Actinomycetales (100)**	**unclassified (<50)**	**unclassified (<50)**
**OTU0924**	**Bacteria (100)**	**Proteobacteria (100)**	**Gammaproteobacteria (99)**	**Pseudomonadales (99)**	**Moraxellaceae (99)**	***Acinetobacter* (98)**
**OTU0925**	**Bacteria (100)**	**Actinobacteria (100)**	**Actinobacteria (100)**	**Pseudonocardiales (100)**	**Pseudonocardiaceae (83)**	**unclassified (<50)**
**OTU0926**	**Bacteria (100)**	**Proteobacteria (100)**	**Alphaproteobacteria (100)**	**Rhizobiales (53)**	**Phyllobacteriaceae (51)**	**unclassified (50)**
**OTU0927**	**Bacteria (100)**	**Actinobacteria (100)**	**Actinobacteria (100)**	**Actinomycetales (100)**	**unclassified (<50)**	**unclassified (<50)**
**OTU0928**	**Bacteria (100)**	**Actinobacteria (100)**	**Actinobacteria (100)**	**Pseudonocardiales (100)**	**Pseudonocardiaceae (79)**	**unclassified (<50)**
**OTU0929**	**Bacteria (100)**	**Actinobacteria (100)**	**Actinobacteria (100)**	**Pseudonocardiales (100)**	**Pseudonocardiaceae (79)**	**unclassified (<50)**
**OTU0930**	**Bacteria (100)**	**Actinobacteria (99)**	**Actinobacteria (99)**	**Actinomycetales (99)**	**unclassified (<50)**	**unclassified (<50)**
**OTU0931**	**Bacteria (100)**	**Actinobacteria (100)**	**Actinobacteria (100)**	**Actinomycetales (99)**	**unclassified (<50)**	**unclassified (<50)**
**OTU0932**	**Bacteria (100)**	**Proteobacteria (100)**	**Alphaproteobacteria (100)**	**Rhizobiales (90)**	**Phyllobacteriaceae (86)**	***Phyllobacterium* (84)**
**OTU0933**	**Bacteria (100)**	**Proteobacteria (100)**	**Betaproteobacteria (100)**	**Burkholderiales (89)**	**Comamonadaceae (66)**	***Brachymonas* (62)**
**OTU0934**	**Bacteria (100)**	**Proteobacteria (100)**	**Alphaproteobacteria (100)**	**unclassified (<50)**	**unclassified (<50)**	**unclassified (<50)**
**OTU0935**	**Bacteria (100)**	**Actinobacteria (100)**	**Actinobacteria (100)**	**Pseudonocardiales (100)**	**Pseudonocardiaceae (75)**	**unclassified (<50)**
**OTU0936**	**Bacteria (100)**	**Firmicutes (100)**	**Bacilli (100)**	**Bacillales (98)**	**Bacillaceae (81) 2**	**unclassified (<50)**
**OTU0937**	**Bacteria (100)**	**Actinobacteria (100)**	**Actinobacteria (100)**	**Actinomycetales (100)**	**unclassified (<50)**	**unclassified (<50)**
**OTU0938**	**Bacteria (100)**	**Actinobacteria (100)**	**Actinobacteria (100)**	**Actinomycetales (100)**	**unclassified (<50)**	**unclassified (<50)**
**OTU0939**	**Bacteria (100)**	**Proteobacteria (100)**	**Alphaproteobacteria (100)**	**Rhizobiales (97)**	**Rhodobiaceae (67)**	***Anderseniella* (63)**
**OTU0940**	**Bacteria (100)**	**Proteobacteria (100)**	**Gammaproteobacteria (100)**	**Vibrionales (100)**	**Vibrionaceae (100)**	***Allomonas* (84)**
**OTU0941**	**Bacteria (100)**	**Actinobacteria (100)**	**Actinobacteria (100)**	**Actinomycetales (100)**	**unclassified (<50)**	**unclassified (<50)**
**OTU0942**	**Bacteria (100)**	**Firmicutes (100)**	**Bacilli (100)**	**Lactobacillales (100)**	**Enterococcaceae (79)**	**unclassified (<50)**
**OTU0943**	**Bacteria (100)**	**Proteobacteria (100)**	**Gammaproteobacteria (98)**	**Vibrionales (94)**	**Vibrionaceae (94)**	***Allomonas* (60)**
**OTU0944**	**Bacteria (100)**	**Proteobacteria (100)**	**Betaproteobacteria (100)**	**Burkholderiales (100)**	**Oxalobacteraceae (100)**	***Duganella* (100)**
**OTU0945**	**Bacteria (100)**	**Actinobacteria (100)**	**Actinobacteria (100)**	**Actinomycetales (100)**	**unclassified (<50)**	**unclassified (<50)**

**OTU0946**	**Bacteria (100)**	**Proteobacteria (100)**	**Gammaproteobacteria (100)**	**Vibrionales (90)**	**Vibrionaceae (90)**	***Photobacterium* (53)**
**OTU0947**	**Bacteria (100)**	**Actinobacteria (100)**	**Actinobacteria (100)**	**Actinomycetales (100)**	**unclassified (<50)**	**unclassified (<50)**
**OTU0948**	**Bacteria (100)**	**Proteobacteria (100)**	**Alphaproteobacteria (100)**	**Rhodobacterales (99)**	**Rhodobacteraceae (99)**	**unclassified (<50)**
**OTU0949**	**Bacteria (100)**	**Actinobacteria (100)**	**Actinobacteria (100)**	**Pseudonocardiales (99)**	**Pseudonocardiaceae (66)**	**unclassified (<50)**
**OTU0950**	**Bacteria (100)**	**Proteobacteria (100)**	**Gammaproteobacteria (100)**	**Pseudomonadales (93)**	**Pseudomonadaceae (93)**	***Pseudomonas* (90)**
**OTU0951**	**Bacteria (100)**	**Actinobacteria (100)**	**Actinobacteria (100)**	**Pseudonocardiales (100)**	**Pseudonocardiaceae (75)**	**unclassified (<50)**
**OTU0952**	**Bacteria (100)**	**Actinobacteria (100)**	**Actinobacteria (100)**	**Pseudonocardiales (100)**	**Pseudonocardiaceae (87)**	**unclassified (<50)**
**OTU0953**	**Bacteria (100)**	**Actinobacteria (100)**	**Actinobacteria (100)**	**Pseudonocardiales (100)**	**Pseudonocardiaceae (89)**	**unclassified (<50)**
**OTU0954**	**Bacteria (100)**	**Actinobacteria (97)**	**Actinobacteria (96)**	**Pseudonocardiales (96)**	**Pseudonocardiaceae (69)**	**unclassified (<50)**
**OTU0955**	**Bacteria (100)**	**Proteobacteria (100)**	**Gammaproteobacteria (100)**	**Pseudomonadales (100)**	**Pseudomonadaceae (100)**	***Pseudomonas* (96)**
**OTU0956**	**Bacteria (100)**	**Actinobacteria (98)**	**Actinobacteria (98)**	**Pseudonocardiales (97)**	**Pseudonocardiaceae (78)**	**unclassified (<50)**
**OTU0957**	**Bacteria (100)**	**Proteobacteria (100)**	**Gammaproteobacteria (100)**	**Vibrionales (100)**	**Vibrionaceae (100)**	***Lucibacterium* (80)**
**OTU0958**	**Bacteria (100)**	**Actinobacteria (100)**	**Actinobacteria (100)**	**Pseudonocardiales (100)**	**Pseudonocardiaceae (78)**	**unclassified (<50)**
**OTU0959**	**Bacteria (100)**	**unclassified (<50)**	**unclassified (<50)**	**unclassified (<50)**	**unclassified (<50)**	**unclassified (<50)**
**OTU0961**	**Bacteria (100)**	**Proteobacteria (100)**	**Gammaproteobacteria (100)**	**Pseudomonadales (100)**	**Moraxellaceae (100)**	***Acinetobacter* (99)**
**OTU0962**	**Bacteria (100)**	**Actinobacteria (100)**	**Actinobacteria (100)**	**Pseudonocardiales (99)**	**Pseudonocardiaceae (82)**	**unclassified (<50)**
**OTU0964**	**Bacteria (100)**	**Actinobacteria (100)**	**Actinobacteria (100)**	**Actinomycetales (100)**	**unclassified (<50)**	**unclassified (<50)**
**OTU0967**	**Bacteria (100)**	**Firmicutes (96)**	**Bacilli (96)**	**Lactobacillales (88)**	**Streptococcaceae (88)**	***Streptococcus* (88)**
**OTU0968**	**Bacteria (100)**	**Proteobacteria (100)**	**Gammaproteobacteria (100)**	**Vibrionales (100)**	**Vibrionaceae (100)**	***Vibrio* (61)**
**OTU0969**	**Bacteria (100)**	**Proteobacteria (100)**	**Alphaproteobacteria (100)**	**Rhizobiales (99)**	**Methylobacteriaceae (88)**	***Methylobacterium* (84)**
**OUT0970**	**Bacteria (100)**	**Proteobacteria (100)**	**Gammaproteobacteria (100)**	**Vibrionales (100)**	**Vibrionaceae (100)**	***Lucibacterium* (76)**
**OUT0972**	**Bacteria (99)**	**Bacteroidetes (98)**	**Bacteroidia (73**	**Bacteroidales (73)**	**Prevotellaceae (68)**	***Alloprevotella* (56)**
**OUT0973**	**Bacteria (100)**	**Actinobacteria (100)**	**Actinobacteria (100)**	**Pseudonocardiales (100)**	**Pseudonocardiaceae (84)**	**unclassified (<50)**

**OUT0974**	**Bacteria (99)**	**Actinobacteria (96)**	**Actinobacteria (96)**	**Pseudonocardiales (94)**	**Pseudonocardiaceae (75)**	**unclassified (<50)**
**OUT0975**	**Bacteria (100)**	**Actinobacteria (99)**	**Actinobacteria (99)**	**Pseudonocardiales (97)**	**Pseudonocardiaceae (70)**	**unclassified (<50)**
**OUT0978**	**Bacteria (100)**	**Proteobacteria (100)**	**Gammaproteobacteria (100)**	**Vibrionales (99)**	**Vibrionaceae (99)**	***Allomonas* (52)**
**OUT0979**	**Bacteria (100)**	**Proteobacteria (100)**	**Gammaproteobacteria (100)**	**Vibrionales (100)**	**Vibrionaceae (100)**	***Listonella* (93)**
**OUT0980**	**Bacteria (100)**	**Actinobacteria (100)**	**Actinobacteria (100)**	**Pseudonocardiales (99)**	**Pseudonocardiaceae (90)**	**unclassified (<50)**
**OUT0983**	**Bacteria (100)**	**Firmicutes (100)**	**Bacilli (100)**	**Lactobacillales (100)**	**Carnobacteriaceae (100)**	***Alloiococcus* (100)**
**OUT0984**	**Bacteria (100)**	**Proteobacteria (100)**	**Gammaproteobacteria (100)**	**Vibrionales (100)**	**Vibrionaceae (100)**	***Allomonas* (92)**
**OUT0985**	**Bacteria (100)**	**Proteobacteria (100)**	**Alphaproteobacteria (100)**	**Rhizobiales (79)**	**Phyllobacteriaceae (68)**	***Phyllobacterium* (68)**
**OUT0987**	**Bacteria (100)**	**Firmicutes (99)**	**Bacilli (96)**	**Bacillales (90)**	**Staphylococcaceae (70)**	***Staphylococcus* (64)**
**OUT0988**	**Bacteria (100)**	**Proteobacteria (100)**	**Gammaproteobacteria (100)**	**Vibrionales (100)**	**Vibrionaceae (100)**	***Allomonas* (77)**
**OUT0989**	**Bacteria (100)**	**Actinobacteria (84)**	**Actinobacteria (84)**	**Actinomycetales (84)**	**unclassified (<50)**	**unclassified (<50)**
**OUT0991**	**Bacteria (100)**	**Proteobacteria (100)**	**Gammaproteobacteria (99)**	**Pseudomonadales (98)**	**Moraxellaceae (98)**	***Acinetobacter* (86)**
**OUT0992**	**Bacteria (100)**	**Proteobacteria (92)**	**Alphaproteobacteria (90)**	**unclassified (<50)**	**unclassified (<50)**	**unclassified (<50)**
**OUT0993**	**Bacteria (100)**	**Actinobacteria (100)**	**Actinobacteria (100)**	**Pseudonocardiales (100)**	**Pseudonocardiaceae (89)**	**unclassified (<50)**
**OUT0994**	**Bacteria (100)**	**Actinobacteria (100)**	**Actinobacteria (100)**	**Pseudonocardiales (100)**	**Pseudonocardiaceae (84)**	**unclassified (<50)**
**OUT0995**	**Bacteria (100)**	**Actinobacteria (100)**	**Actinobacteria (100)**	**Micrococcales (100)**	**Intrasporangiaceae (96)**	**unclassified (<50)**
**OUT0996**	**Bacteria (100)**	**Actinobacteria (99)**	**Actinobacteria (99)**	**Pseudonocardiales (99)**	**Pseudonocardiaceae (76)**	**unclassified (<50)**
**OUT0997**	**Bacteria (100)**	**Proteobacteria (100)**	**Gammaproteobacteria (100)**	**Pseudomonadales (100)**	**Moraxellaceae (100)**	***Acinetobacter* (99)**
**OUT0998**	**Bacteria (100)**	**Proteobacteria (100)**	**Gammaproteobacteria (100)**	**Vibrionales (100)**	**Vibrionaceae (100)**	***Allomonas* (51)**
**OUT1000**	**Bacteria (100)**	**Firmicutes (100)**	**Bacilli (100)**	**Bacillales (97)**	**unclassified (<50)**	**unclassified (<50)**
**OUT1001**	**Bacteria (100)**	**Proteobacteria (100)**	**Gammaproteobacteria (100)**	**Vibrionales (92)**	**Vibrionaceae (92)**	***Allomonas* (65)**
**OTU1003**	**Bacteria (100)**	**Proteobacteria (100)**	**Gammaproteobacteria (100)**	**Vibrionales (75)**	**Vibrionaceae (75)**	**unclassified (<50)**
**OTU1004**	**Bacteria (100)**	**Proteobacteria (100)**	**Gammaproteobacteria (100)**	**Vibrionales (94)**	**Vibrionaceae (94)**	***Lucibacterium* (50)**

**OTU1005**	**Bacteria (100)**	**Proteobacteria (100)**	**Gammaproteobacteria (99)**	**Vibrionales (99)**	**Vibrionaceae (99)**	***Allomonas* (83)**
**OTU1006**	**Bacteria (100)**	**Proteobacteria (100)**	**Gammaproteobacteria (100)**	**Pasteurellales (100)**	**Pasteurellaceae (100)**	**unclassified (<50)**
**OTU1007**	**Bacteria (100)**	**Proteobacteria (97)**	**Alphaproteobacteria (92)**	**Sphingomonadales (76)**	**Sphingomonadaceae (75)**	***Sphingobium* (53)**
**OTU1008**	**Bacteria (100)**	**Actinobacteria (82)**	**Actinobacteria (82)**	**Pseudonocardiales (82)**	**Pseudonocardiaceae (52)**	**unclassified (<50)**
**OTU1010**	**Bacteria (100)**	**Proteobacteria (100)**	**Gammaproteobacteria (100)**	**Vibrionales (96)**	**Vibrionaceae (96)**	***Allomonas* (52)**
**OTU1014**	**Bacteria (100)**	**Proteobacteria (100)**	**Gammaproteobacteria (100)**	**Vibrionales (100)**	**Vibrionaceae (100)**	***Vibrio* (51)**
**OTU1015**	**Bacteria (100)**	**Proteobacteria (100)**	**Gammaproteobacteria (100)**	**Vibrionales (100)**	**Vibrionaceae (100)**	***Listonella* (97)**
**OTU1017**	**Bacteria (100)**	**Fusobacteria (100)**	**Fusobacteriia (100)**	**Fusobacteriales (100)**	**Fusobacteriaceae (100)**	***Fusobacterium* (100)**
**OTU1018**	**Bacteria (100)**	**Actinobacteria (100)**	**Actinobacteria (100)**	**Actinomycetales (99)**	**unclassified (<50)**	**unclassified (<50)**
**OTU1021**	**Bacteria (100)**	**Actinobacteria (100)**	**Actinobacteria (100)**	**Micrococcales (100)**	**Dermabacteraceae (100)**	***Brachybacterium* (100)**
**OTU1022**	**Bacteria (100)**	**Proteobacteria (100)**	**Gammaproteobacteria (100)**	**Pasteurellales (100)**	**Pasteurellaceae (100)**	
**OTU1023**	**Bacteria (100)**	**Proteobacteria (100)**	**Alphaproteobacteria (100)**	**Sphingomonadales (93)**	**Sphingomonadaceae (93)**	***Sphingomonas* (92)**
**OTU1024**	**Bacteria (100)**	**Actinobacteria (100)**	**Actinobacteria (100)**	**Pseudonocardiales (99)**	**Pseudonocardiaceae (98)**	***Saccharopolyspora* (65)**
**OTU1025**	**Bacteria (100)**	**Proteobacteria (100)**	**Gammaproteobacteria (100)**	**Vibrionales (96)**	**Vibrionaceae (96)**	***Allomonas* (57)**
**OTU1026**	**Bacteria (100)**	**Proteobacteria (100)**	**Gammaproteobacteria (100)**	**Vibrionales (100)**	**Vibrionaceae (100)**	***Listonella* (94)**
**OTU1027**	**Bacteria (100)**	**Proteobacteria (100)**	**Alphaproteobacteria (100)**	**Rhodobacterales (95)**	**Rhodobacteraceae (95)**	***Amaricoccus* (82)**
**OTU1028**	**Bacteria (100)**	**Actinobacteria (100)**	**Actinobacteria (100)**	**Pseudonocardiales (100)**	**Pseudonocardiaceae (95)**	**unclassified (<50)**
**OTU1029**	**Bacteria (100)**	**Firmicutes (100)**	**Bacilli (100)**	**Bacillales (99)**	**unclassified (<50)**	**unclassified (<50)**
**OTU1031**	**Bacteria (100)**	**Proteobacteria (100)**	**Gammaproteobacteria (100)**	**Pseudomonadales (100)**	**Moraxellaceae (100)**	***Acinetobacter* (93)**
**OTU1032**	**Bacteria (100)**	**Actinobacteria (100)**	**Actinobacteria (100)**	**Pseudonocardiales (100)**	**Pseudonocardiaceae (75)**	**unclassified (<50)**
**OTU1033**	**Bacteria (100)**	**Actinobacteria (100)**	**Actinobacteria (100)**	**Corynebacteriales (100)**	**Corynebacteriaceae (93)**	***Corynebacterium* (85)**
**OTU1034**	**Bacteria (100)**	**Proteobacteria (100)**	**Alphaproteobacteria (100)**	**Sphingomonadales (98)**	**Erythrobacteraceae (98)**	***Erythrobacter* (98)**
**OUT1035**	**Bacteria (100)**	**Firmicutes (97)**	**Bacilli (96)**	**Lactobacillales (95)**	**Streptococcaceae (93)**	***Streptococcus* (93)**
**OUT1036**	**Bacteria (100)**	**Proteobacteria (100)**	**Gammaproteobacteria (100)**	**Vibrionales (100)**	**Vibrionaceae (100)**	***Listonella* (96)**

OUT1037	Bacteria (100)	Proteobacteria (100)	Gammaproteobacteria (100)	Vibrionales (100)	Vibrionaceae (100)	*Lucibacterium* (62)
OUT1038	Bacteria (100)	Proteobacteria (100)	Betaproteobacteria (100)	Neisseriales (96)	Neisseriaceae (96)	
OUT1040	Bacteria (100)	Proteobacteria (100)	Gammaproteobacteria (100)	Aeromonadales (59)	Aeromonadaceae (59)	unclassified (<50)
OUT1041	Bacteria (100)	Actinobacteria (100)	Actinobacteria (100)	Actinobacteria (100)	unclassified (<50)	unclassified (<50)
OUT1043	Bacteria (100)	Proteobacteria (100)	Gammaproteobacteria (100)	Aeromonadales (84)	Aeromonadaceae (84)	*Aeromonas* (84)
OUT1044	Bacteria (100)	Firmicutes (100)	Bacilli (100)	Lactobacillales (94)	Enterococcaceae (80)	*Enterococcus* (75)
OUT1045	Bacteria (100)	Proteobacteria (100)	Gammaproteobacteria (100)	Vibrionales (98)	Vibrionaceae (98)	unclassified (<50)
OUT1046	Bacteria (100)	Firmicutes (100)	Bacilli (99)	Bacillales (98)	Staphylococcaceae (94)	*Staphylococcus* (89)
OUT1047	Bacteria (99)	Actinobacteria (99)	Actinobacteria (99)	Pseudonocardiales (95)	Pseudonocardiaceae (70)	unclassified (<50)
OUT1048	Bacteria (100)	Actinobacteria (100)	Actinobacteria (100)	Actinomycetales (100)	Micrococcaceae (100)	*Micrococcus* (100)
OUT1049	Bacteria (100)	Proteobacteria (100)	Gammaproteobacteria (100)	Vibrionales (85)	Vibrionaceae (85)	unclassified (<50)
OUT1050	Bacteria (100)	Proteobacteria (100)	Gammaproteobacteria (100)	Vibrionales (100)	Vibrionaceae (100)	*Listonella* (87)
OUT1051	Bacteria (100)	Proteobacteria (100)	Gammaproteobacteria (100)	Pasteurellales (100)	Pasteurellaceae (100)	unclassified (<50)
OUT1052	Bacteria (100)	Proteobacteria (100)	Gammaproteobacteria (100)	Vibrionales (93)	Vibrionaceae (93)	unclassified (<50)
OUT1053	Bacteria (100)	Actinobacteria (100)	Actinobacteria (100)	Pseudonocardiales (97)	Pseudonocardiaceae (88)	unclassified (<50)
OUT1054	Bacteria (100)	Proteobacteria (100)	Betaproteobacteria (100)	Burkholderiales (100)	Oxalobacteraceae (100)	unclassified (<50)
OUT1055	Bacteria (100)	Firmicutes (100)	Bacilli (100)	Lactobacillales (96)	unclassified (<50)	unclassified (<50)
OUT1057	Bacteria (99)	Firmicutes (98)	Bacilli (98)	Lactobacillales (97)	Streptococcaceae (92)	*Streptococcus* (92)
OUT1058	Bacteria (100)	Actinobacteria (100)	Actinobacteria (100)	Pseudonocardiales (100)	Pseudonocardiaceae (89)	unclassified (<50)
OUT1059	Bacteria (100)	Proteobacteria (100)	Gammaproteobacteria (100)	Pseudomonadales (96)	Pseudomonadaceae (96)	*Azotobacter* (52)
OUT1061	Bacteria (100)	Firmicutes (100)	Bacilli (100)	Lactobacillales (90)	Enterococcaceae (54)	unclassified (<50)
OUT1062	Bacteria (100)	Actinobacteria (100)	Actinobacteria (100)	Pseudonocardiales (100)	Pseudonocardiaceae (84)	unclassified (<50)
OTU1063	Bacteria (100)	Proteobacteria (100)	Gammaproteobacteria (100)	Vibrionales (100)	Vibrionaceae (100)	*Allomonas* (65)
OTU1064	Bacteria (100)	Proteobacteria (100)	Gammaproteobacteria (100)	Vibrionales (100)	Vibrionaceae (100)	*Allomonas* (62)

**OTU1065**	**Bacteria (100)**	**Proteobacteria (100)**	**Gammaproteobacteria (100)**	**Enterobacteriales (100)**	**Enterobacteriaceae (100)**	**unclassified (<50)**
**OTU1066**	**Bacteria (100)**	**Actinobacteria (100)**	**Actinobacteria (100)**	**Pseudonocardiales (100)**	**Pseudonocardiaceae (95)**	**unclassified (<50)**
**OTU1067**	**Bacteria (100)**	**Actinobacteria (100)**	**Actinobacteria (100)**	**Corynebacteriales (100)**	**Corynebacteriaceae (94)**	***Corynebacterium*** **(94)**
**OTU1068**	**Bacteria (100)**	**Actinobacteria (100)**	**Actinobacteria (100)**	**Corynebacteriales (100)**	**Corynebacteriaceae (62)**	***Corynebacterium*** **(58)**
**OTU1069**	**Bacteria (100)**	**Proteobacteria (100)**	**Gammaproteobacteria (100)**	**Vibrionales (100)**	**Vibrionaceae (100)**	***Aliivibrio*** **(95)**
**OTU1070**	**Bacteria (100)**	**Proteobacteria (100)**	**Gammaproteobacteria (100)**	**Vibrionales (96)**	**Vibrionaceae (96)**	**unclassified (<50)**
**OTU1071**	**Bacteria (100)**	**Actinobacteria (100)**	**Actinobacteria (100)**	**Pseudonocardiales (99)**	**Pseudonocardiaceae (80)**	**unclassified (<50)**
**OTU1072**	**Bacteria (100)**	**Proteobacteria (100)**	**Gammaproteobacteria (100)**	**Vibrionales (100)**	**Vibrionaceae (100)**	**unclassified (<50)**
**OTU1073**	**Bacteria (100)**	**Proteobacteria (99)**	**Gammaproteobacteria (99)**	**Pseudomonadales (97)**	**Moraxellaceae (97)**	***Acinetobacter*** **(92)**
**OTU1074**	**Bacteria (100)**	**Proteobacteria (100)**	**Gammaproteobacteria (100)**	**Vibrionales (100)**	**Vibrionaceae (100)**	***Lucibacterium*** **(69)**
**OTU1075**	**Bacteria (100)**	**Proteobacteria (100)**	**Alphaproteobacteria (100)**	**Rhizobiales (79)**	**Phyllobacteriaceae (78)**	***Phyllobacterium*** **(77)**
**OTU1077**	**Bacteria (100)**	**Actinobacteria (98)**	**Actinobacteria (98)**	**Pseudonocardiales (92)**	**Pseudonocardiaceae (66)**	**unclassified (<50)**
**OTU1078**	**Bacteria (100)**	**Actinobacteria (100)**	**Actinobacteria (100)**	**Pseudonocardiales (100)**	**Pseudonocardiaceae (85)**	**unclassified (<50)**
**OTU1080**	**Bacteria (100)**	**Actinobacteria (100)**	**Actinobacteria (100)**	**Pseudonocardiales (98)**	**Pseudonocardiaceae (79)**	**unclassified (<50)**
**OTU1083**	**Bacteria (100)**	**Actinobacteria (100)**	**Actinobacteria (100)**	**Pseudonocardiales (100)**	**Pseudonocardiaceae (89)**	**unclassified (<50)**
**OTU1085**	**Bacteria (100)**	**Proteobacteria (100)**	**Gammaproteobacteria (100)**	**Vibrionales (99)**	**Vibrionaceae (99)**	***Enterovibrio*** **(68)**
**OTU1086**	**Bacteria (100)**	**Actinobacteria (100)**	**Actinobacteria (100)**	**Pseudonocardiales (98)**	**Pseudonocardiaceae (88)**	**unclassified (<50)**
**OTU1087**	**Bacteria (100)**	**Proteobacteria (100)**	**Gammaproteobacteria (100)**	**Vibrionales (82)**	**Vibrionaceae (82)**	***Allomonas*** **(56)**
**OTU1088**	**Bacteria (100)**	**Proteobacteria (100)**	**Gammaproteobacteria (100)**	**Chromatiales (86)**	**Chromatiaceae (86)**	**unclassified (<50)**
**OTU1089**	**Bacteria (100)**	**Actinobacteria (99)**	**Actinobacteria (99)**	**Pseudonocardiales (99)**	**unclassified (<50)**	**unclassified (<50)**
**OTU1090**	**Bacteria (100)**	**Proteobacteria (100)**	**Gammaproteobacteria (100)**	**Vibrionales (96)**	**Vibrionaceae (96)**	**unclassified (<50)**
**OTU1091**	**Bacteria (100)**	**Actinobacteria (100)**	**Actinobacteria (100)**	**Corynebacteriales (100)**	**Corynebacteriaceae (99)**	***Turicella*** **(90)**
**OTU1093**	**Bacteria (100)**	**Actinobacteria (99)**	**Actinobacteria (99)**	**Pseudonocardiales (98)**	**Pseudonocardiaceae (86)**	**unclassified (<50)**
**OTU1095**	**Bacteria (100)**	**Actinobacteria (100)**	**Actinobacteria (100)**	**Pseudonocardiales (100)**	**Pseudonocardiaceae (89)**	**unclassified (<50)**

**OTU1096**	**Bacteria (100)**	**Proteobacteria (100)**	**Alphaproteobacteria (100)**	**Rhizobiales (99)**	**Phyllobacteriaceae (97)**	***Phyllobacterium* (96)**
**OTU1097**	**Bacteria (100)**	**Proteobacteria (100)**	**Alphaproteobacteria (100)**	**Rhizobiales (56)**	**Phyllobacteriaceae (54)**	***Phyllobacterium* (54)**
**OTU1098**	**Bacteria (100)**	**Actinobacteria (100)**	**Actinobacteria (100)**	**Pseudonocardiales (99)**	**Pseudonocardiaceae (88)**	**unclassified (<50)**
**OTU1100**	**Bacteria (100)**	**Proteobacteria (100)**	**Gammaproteobacteria (100)**	**Pseudomonadales (97)**	**Pseudomonadaceae (97)**	***Azotobacter* (72)**
**OTU1101**	**Bacteria (100)**	**Proteobacteria (100)**	**Alphaproteobacteria (100)**	**unclassified (<50)**	**unclassified (<50)**	**unclassified (<50)**
**OTU1102**	**Bacteria (100)**	**Proteobacteria (100)**	**Alphaproteobacteria (100)**	**Rhizobiales (100)**	**Methylobacteriaceae (100)**	***Microvirga* (100)**
**OTU1104**	**Bacteria (100)**	**Actinobacteria (100)**	**Actinobacteria (100)**	**Corynebacteriales (99)**	**Corynebacteriaceae (90)**	***Corynebacterium* (90)**
**OTU1105**	**Bacteria (100)**	**Actinobacteria (100)**	**Actinobacteria (100)**	**Pseudonocardiales (99)**	**Pseudonocardiaceae (76)**	**unclassified (<50)**
**OTU1106**	**Bacteria (100)**	**Actinobacteria (100)**	**Actinobacteria (100)**	**Pseudonocardiales (100)**	**Pseudonocardiaceae (74)**	**unclassified (<50)**
**OTU1108**	**Bacteria (100)**	**Proteobacteria (100)**	**Gammaproteobacteria (100)**	**Alteromonadales (100)**	**Pseudoalteromonadaceae (97)**	***Pseudoalteromonas* (96)**
**OTU1112**	**Bacteria (100)**	**Proteobacteria (100)**	**Gammaproteobacteria (100)**	**Pseudomonadales (90)**	**Pseudomonadaceae (90)**	**unclassified (<50)**
**OTU1113**	**Bacteria (100)**	**Actinobacteria (100)**	**Actinobacteria (100)**	**Pseudonocardiales (100)**	**Pseudonocardiaceae (78)**	**unclassified (<50)**
**OTU1114**	**Bacteria (100)**	**Proteobacteria (100)**	**Gammaproteobacteria (100)**	**Vibrionales (100)**	**Vibrionaceae (100)**	***Allomonas* (72)**
**OTU1115**	**Bacteria (100)**	**Proteobacteria (100)**	**Alphaproteobacteria (100)**	**Sphingomonadales (100)**	**Sphingomonadaceae (100)**	***Sphingomonas* (100)**
**OTU1118**	**Bacteria (100)**	**Proteobacteria (100)**	**Alphaproteobacteria (100)**	**Rhodobacterales (100)**	**Rhodobacteraceae (100)**	***Paracoccus* (91)**
**OTU1119**	**Bacteria (100)**	**Actinobacteria (100)**	**Actinobacteria (100)**	**Corynebacteriales (100)**	**Corynebacteriaceae (97)**	***Corynebacterium* (94)**
**OTU1120**	**Bacteria (100)**	**Proteobacteria (100)**	**Gammaproteobacteria (100)**	**Vibrionales (100)**	**Vibrionaceae (100)**	***Allomonas* (71)**
**OTU1121**	**Bacteria (100)**	**Firmicutes (100)**	**Bacilli (100)**	**Lactobacillales (100)**	**Streptococcaceae (100)**	***Lactococcus* (100)**
**OTU1123**	**Bacteria (100)**	**Proteobacteria (100)**	**Gammaproteobacteria (100)**	**Aeromonadales (100)**	**Aeromonadaceae (100)**	***Aeromonas* (100)**
**OTU1124**	**Bacteria (100)**	**Proteobacteria (100)**	**Gammaproteobacteria (100)**	**Vibrionales (100)**	**Vibrionaceae (100)**	***Allomonas* (67)**
**OTU1125**	**Bacteria (100)**	**Actinobacteria (99)**	**Actinobacteria (99)**	**Pseudonocardiales (99)**	**Pseudonocardiaceae (94)**	**unclassified (<50)**
**OTU1126**	**Bacteria (100)**	**Actinobacteria (100)**	**Actinobacteria (100)**	**Pseudonocardiales (100)**	**Pseudonocardiaceae (69)**	**unclassified (<50)**
**OTU1127**	**Bacteria (100)**	**Proteobacteria (100)**	**Gammaproteobacteria (100)**	**Vibrionales (100)**	**Vibrionaceae (100)**	***Listonella* (93)**
**OTU1128**	**Bacteria (100)**	**Proteobacteria (100)**	**Gammaproteobacteria (100)**	**unclassified (<50)**	**unclassified (<50)**	**unclassified (<50)**

**OTU1129**	**Bacteria (100)**	**Proteobacteria (100)**	**Gammaproteobacteria (100)**	**Pseudomonadales (59)**	**Moraxellaceae (59)**	***Acinetobacter* (55)**
**OTU1130**	**Bacteria (100)**	**Proteobacteria (100)**	**Gammaproteobacteria (100)**	**Vibrionales (100)**	**Vibrionaceae (100)**	***Allomonas* (50)**
**OTU1131**	**Bacteria (100)**	**Proteobacteria (100)**	**Gammaproteobacteria (100)**	**Enterobacteriales (66)**	**Enterobacteriaceae (66)**	**unclassified (<50)**
**OTU1132**	**Bacteria (100)**	**Actinobacteria (97)**	**Actinobacteria (97)**	**Acidimicrobiales (85)**	**unclassified (<50)**	**unclassified (<50)**
**OTU1133**	**Bacteria (100)**	**Proteobacteria (100)**	**Gammaproteobacteria (100)**	**Vibrionales (99)**	**Vibrionaceae (99)**	***Allomonas* (84)**
**OTU1134**	**Bacteria (100)**	**Actinobacteria (100)**	**Actinobacteria (100)**	**Corynebacteriales (100)**	**Corynebacteriaceae (60)**	***Corynebacterium* (61)**
**OTU1135**	**Bacteria (100)**	**Proteobacteria (100)**	**Gammaproteobacteria (100)**	**Enterobacteriales (90)**	**Enterobacteriaceae (90)**	**unclassified (<50)**
**OTU1136**	**Bacteria (100)**	**Proteobacteria (99)**	**Gammaproteobacteria (99)**	**Vibrionales (95)**	**Vibrionaceae (95)**	**unclassified (<50)**
**OTU1137**	**Bacteria (100)**	**Proteobacteria (100)**	**Gammaproteobacteria (100)**	**Vibrionales (100)**	**Vibrionaceae (100)**	***Allomonas* (76)**
**OTU1138**	**Bacteria (100)**	**Proteobacteria (100)**	**Gammaproteobacteria (100)**	**Vibrionales (100)**	**Vibrionaceae (100)**	***Listonella* (75)**
**OTU1139**	**Bacteria (100)**	**Proteobacteria (100)**	**Gammaproteobacteria (100)**	**Pseudomonadales (99)**	**Moraxellaceae (99)**	***Acinetobacter* (93)**
**OTU1140**	**Bacteria (100)**	**Proteobacteria (100)**	**Gammaproteobacteria (100)**	**Pseudomonadales (100)**	**Moraxellaceae (100)**	***Acinetobacter* (93)**
**OTU1141**	**Bacteria (100)**	**Firmicutes (100)**	**Bacilli (100)**	**Lactobacillales (100)**	**Streptococcaceae (98)**	***Streptococcus* (97)**
**OTU1142**	**Bacteria (100)**	**Bacteroidetes (100)**	**Bacteroidia (97)**	**Bacteroidales (97)**	**unclassified (<50)**	**unclassified (<50)**
**OTU1143**	**Bacteria (100)**	**Actinobacteria (100)**	**Actinobacteria (100)**	**Pseudonocardiales (100)**	**Pseudonocardiaceae (80)**	**unclassified (<50)**
**OTU1144**	**Bacteria (100)**	**Proteobacteria (100)**	**Alphaproteobacteria (100)**	**Rhodobacterales (97)**	**Rhodobacteraceae (97)**	***Amaricoccus* (90)**
**OTU1145**	**Bacteria (100)**	**Proteobacteria (100)**	**Gammaproteobacteria (100)**	**Vibrionales (100)**	**Vibrionaceae (100)**	**unclassified (<50)**
**OTU1146**	**Bacteria (100)**	**Proteobacteria (100)**	**Gammaproteobacteria (100)**	**Vibrionales (80)**	**Vibrionaceae (80)**	***Photobacterium* (61)**
**OTU1147**	**Bacteria (100)**	**Proteobacteria (100)**	**Gammaproteobacteria (100)**	**Vibrionales (100)**	**Vibrionaceae (100)**	***Allomonas* (80)**
**OTU1148**	**Bacteria (100)**	**Proteobacteria (100)**	**Gammaproteobacteria (100)**	**Vibrionales (95)**	**Vibrionaceae (95)**	***Photobacterium* (86)**
**OTU1149**	**Bacteria (100)**	**Proteobacteria (100)**	**Gammaproteobacteria (100)**	**Vibrionales (100)**	**Vibrionaceae (100)**	***Listonella* (88)**
**OTU1150**	**Bacteria (100)**	**Proteobacteria (100)**	**Epsilonproteobacteria (100)**	**Campylobacterales (100)**	**Helicobacteraceae (100)**	***Sulfurovum* (100)**
**OTU1151**	**Bacteria (100)**	**Proteobacteria (100)**	**Gammaproteobacteria (100)**	**Vibrionales (100)**	**Vibrionaceae (100)**	***Allomonas* (83)**
**OTU1152**	**Bacteria (100)**	**Proteobacteria (100)**	**Gammaproteobacteria (100)**	**Pseudomonadales (100)**	**Moraxellaceae (100)**	***Acinetobacter* (99)**

OTU1153	Bacteria (100)	Bacteroidetes (100)	Cytophagia (98)	Cytophagales (98)	Flammeovirgaceae (94)	*Marinoscillum* (57)
OTU1154	Bacteria (100)	Firmicutes (100)	Bacilli (98)	Bacillales (97)	Staphylococcaceae (88)	*Staphylococcus* (76)
OTU1155	Bacteria (100)	Firmicutes (100)	Bacilli (100)	Bacillalles (100)	Planococcaceae (99)	*Sporosarcina* (99)
OTU1156	Bacteria (100)	Proteobacteria (100)	Gammaproteobacteria (100)	Vibrionales (100)	Vibrionaceae (100)	*Listonella* (90)
OTU1157	Bacteria (100)	Proteobacteria (100)	Gammaproteobacteria (100)	Vibrionales (100)	Vibrionaceae (100)	*Listonella* (85)
OTU1158	Bacteria (100)	Proteobacteria (99)	Gammaproteobacteria (97)	Pseudomonadales (67)	Moraxellaceae (50)	unclassified (<50)
OTU1159	Bacteria (100)	Proteobacteria (100)	Gammaproteobacteria (100)	Vibrionales (100)	Vibrionaceae (100)	*Allomonas* (78)
OTU1160	Bacteria (100)	Proteobacteria (100)	Gammaproteobacteria (100)	Vibrionales (100)	Vibrionaceae (100)	*Allomonas* (73)
OTU1161	Bacteria (100)	Actinobacteria (100)	Actinobacteria (100)	Pseudonocardiales (100)	Pseudonocardiaceae (65)	unclassified (<50)
OTU1162	Bacteria (100)	Proteobacteria (100)	Gammaproteobacteria (100)	Enterobacteriales (99)	Enterobacteriaceae (99)	unclassified (<50)
OTU1163	Bacteria (100)	Proteobacteria (100)	Alphaproteobacteria (100)	Rhizobiales (57)	unclassified (<50)	unclassified (<50)
OTU1164	Bacteria (100)	Proteobacteria (100)	Gammaproteobacteria (100)	Vibrionales (100)	Vibrionaceae (100)	*Allomonas* (83)
OTU1165	Bacteria (100)	Proteobacteria (100)	Gammaproteobacteria (100)	Vibrionales (94)	Vibrionaceae (94)	unclassified (<50)
OTU1166	Bacteria (100)	Actinobacteria (100)	Actinobacteria (100)	Pseudonocardiales (100)	Pseudonocardiaceae (97)	unclassified (<50)
OTU1167	Bacteria (100)	Proteobacteria (100)	Gammaproteobacteria (94	Chromatiales (89)	Chromatiaceae (88)	*Halochromatium* (75)
OTU1168	Bacteria (100)	Proteobacteria (100)	Alphaproteobacteria (100)	Rhodobacterales (94)	Rhodobacteraceae (94)	unclassified (<50)
OTU1169	Bacteria (100)	Actinobacteria (100)	Actinobacteria (100)	Pseudonocardiales (100)	unclassified (<50)	unclassified (<50)
OTU1170	Bacteria (100)	Proteobacteria (100)	Gammaproteobacteria (100)	Vibrionales (99)	Vibrionaceae (99)	unclassified (<50)
OTU1171	Bacteria (100)	Proteobacteria (100)	Gammaproteobacteria (100)	Vibrionales (100)	Vibrionaceae (100)	*Vibrio* (50)
OTU1172	Bacteria (100)	Firmicutes (100)	Clostridia (100)	Clostridiales (100)	Clostridiales_Incertae Sedis XI (100)	*Peptoniphilus* (100)
OTU1173	Bacteria (100)	Proteobacteria (100)	Gammaproteobacteria (100)	Vibrionales (100)	Vibrionaceae (100)	*Listonella* (74)
OTU1174	Bacteria (100)	Proteobacteria (100)	Gammaproteobacteria (100)	Vibrionales (89)	Vibrionaceae (89)	unclassified (<50)
OUT1175	Bacteria (100)	Proteobacteria (100)	Gammaproteobacteria (100)	Enterobacteriales (100)	Enterobacteriaceae (100)	unclassified (<50)
OUT1176	Bacteria (100)	Actinobacteria (100)	Actinobacteria (100)	Pseudonocardiales (99)	Pseudonocardiaceae (92)	unclassified (<50)

**OTU1177**	**Bacteria (100)**	**Firmicutes (100)**	**Bacilli (99)**	**Bacillales (94)**	**Staphylococcaceae (84)**	***Staphylococcus* (73)**
**OTU1178**	**Bacteria (100)**	**Proteobacteria (100)**	**Alphaproteobacteria (100)**	**Rhodobacterales (99)**	**Rhodobacteraceae (99)**	**unclassified (<50)**
**OTU1179**	**Bacteria (94)**	**Bacteroidetes (85)**	**Bacteroidia (68)**	**Bacteroidales (68)**	**Prevotellaceae (63)**	**unclassified (<50)**
**OTU1180**	**Bacteria (100)**	**Firmicutes (100)**	**Bacilli (100)**	**Bacillalles (100)**	**Bacillales_Incertae Sedis XII (100)**	***Exiguobacterium* (100)**
**OTU1181**	**Bacteria (100)**	**Actinobacteria (100)**	**Actinobacteria (100)**	**Pseudonocardiales (99)**	**Pseudonocardiaceae (77)**	**unclassified (<50)**
**OTU1182**	**Bacteria (100)**	**Actinobacteria (97)**	**Actinobacteria (97)**	**Pseudonocardiales (94)**	**Pseudonocardiaceae (69)**	**unclassified (<50)**
**OTU1183**	**Bacteria (100)**	**Actinobacteria (100)**	**Actinobacteria (100)**	**Pseudonocardiales (100)**	**Pseudonocardiaceae (79)**	**unclassified (<50)**
**OTU1184**	**Bacteria (100)**	**Bacteroidetes (85)**	**unclassified (<50)**	**unclassified (<50)**	**unclassified (<50)**	**unclassified (<50)**
**OTU1185**	**Bacteria (100)**	**Actinobacteria (98)**	**Actinobacteria (98)**	**Pseudonocardiales (98)**	**Pseudonocardiaceae (62)**	**unclassified (<50)**
**OTU1186**	**Bacteria (100)**	**Cyanobacteria/ Chloroplast(78)**	**unclassified (<50)**	**unclassified (<50)**	**unclassified (<50)**	**unclassified (<50)**
**OTU1187**	**Bacteria (100)**	**Proteobacteria (100)**	**Gammaproteobacteria (100)**	**Alteromonadales (100)**	**Pseudoalteromonadaceae (96)**	***Pseudoalteromonas* (95)**
**OTU1188**	**Bacteria (100)**	**Proteobacteria (100)**	**Gammaproteobacteria (100)**	**Vibrionales (98)**	**Vibrionaceae (98)**	***Allomonas* (67)**
**OTU1189**	**Bacteria (100)**	**Actinobacteria (100)**	**Actinobacteria (100)**	**Pseudonocardiales (100)**	**Pseudonocardiaceae (75)**	**unclassified (<50)**
**OTU1190**	**Bacteria (100)**	**Actinobacteria (100)**	**Actinobacteria (100)**	**Pseudonocardiales (99)**	**Pseudonocardiaceae (69)**	**unclassified (<50)**
**OTU1191**	**Bacteria (100)**	**Actinobacteria (98)**	**Actinobacteria (98)**	**Pseudonocardiales (96)**	**Pseudonocardiaceae (70)**	**unclassified (<50)**
**OTU1192**	**Bacteria (100)**	**Proteobacteria (100)**	**Alphaproteobacteria (100)**	**Rhodobacterales (89)**	**Rhodobacteraceae (89)**	***Roseicyclus* (52)**
**OTU1193**	**Bacteria (100)**	**Proteobacteria (100)**	**Gammaproteobacteria (100)**	**Vibrionales (100)**	**Vibrionaceae (100)**	***Allomonas* (73)**
**OTU1194**	**Bacteria (100)**	**Proteobacteria (100)**	**Gammaproteobacteria (99)**	**Alteromonadales (68)**	**unclassified (<50)**	**unclassified (<50)**
**OTU1195**	**Bacteria (100)**	**Proteobacteria (100)**	**Gammaproteobacteria (100)**	**Vibrionales (100)**	**Vibrionaceae (100)**	***Listonella* (81)**
**OTU1196**	**Bacteria (100)**	**Proteobacteria (100)**	**Gammaproteobacteria (100)**	**Vibrionales (68)**	**Vibrionaceae (68)**	**unclassified (<50)**
**OTU1197**	**Bacteria (100)**	**Proteobacteria (100)**	**Gammaproteobacteria (100)**	**Pseudomonadales (100)**	**Moraxellaceae (100)**	***Acinetobacter* (97)**
**OTU1198**	**Bacteria (100)**	**Actinobacteria (100)**	**Actinobacteria (100)**	**Pseudonocardiales (100)**	**Pseudonocardiaceae (90)**	**unclassified (<50)**
**OTU1199**	**Bacteria (100)**	**Proteobacteria (100)**	**Gammaproteobacteria (100)**	**Vibrionales (99)**	**Vibrionaceae (99)**	***Allomonas* (76)**
**OTU1200**	**Bacteria (100)**	**Proteobacteria (99)**	**Gammaproteobacteria (99)**	**Vibrionales (90)**	**Vibrionaceae (90)**	***Allomonas* (51)**

**OTU1201**	**Bacteria (100)**	**Proteobacteria (100)**	**Gammaproteobacteria (100)**	**Vibrionales (100)**	**Vibrionaceae (100)**	**unclassified (<50)**
**OTU1202**	**Bacteria (100)**	**Proteobacteria (100)**	**Gammaproteobacteria (100)**	**Vibrionales (100)**	**Vibrionaceae (100)**	***Allomonas* (71)**
**OTU1203**	**Bacteria (100)**	**Actinobacteria (97)**	**Actinobacteria (97)**	**Pseudonocardiales (97)**	**Pseudonocardiaceae (87)**	**unclassified (<50)**
**OTU1204**	**Bacteria (100)**	**Actinobacteria (100)**	**Actinobacteria (100)**	**Pseudonocardiales (100)**	**Pseudonocardiaceae (80)**	**unclassified (<50)**
**OTU1205**	**Bacteria (100)**	**Actinobacteria (100)**	**Actinobacteria (100)**	**Pseudonocardiales (100)**	**Pseudonocardiaceae (100)**	**unclassified (<50)**
**OTU1206**	**Bacteria (100)**	**Actinobacteria (100)**	**Actinobacteria (100)**	**Pseudonocardiales (100)**	**Pseudonocardiaceae (62)**	**unclassified (<50)**
**OTU1207**	**Bacteria (100)**	**Actinobacteria (100)**	**Actinobacteria (100)**	**Pseudonocardiales (100)**	**Pseudonocardiaceae (89)**	**unclassified (<50)**
**OTU1208**	**Bacteria (100)**	**Proteobacteria (100)**	**Gammaproteobacteria (100)**	**Aeromonadales (66)**	**Aeromonadaceae (66)**	***Aeromonas* (54)**
**OTU1209**	**Bacteria (100)**	**Actinobacteria (96)**	**Actinobacteria (96)**	**Acidimicrobiales (51)**	**unclassified (<50)**	**unclassified (<50)**
**OTU1210**	**Bacteria (100)**	**Proteobacteria (100)**	**Gammaproteobacteria (100)**	**Pseudomonadales (77)**	**Pseudomonadaceae (77)**	***Serpens* (63)**
**OTU1211**	**Bacteria (100)**	**Proteobacteria (100)**	**Gammaproteobacteria (100)**	**Pseudomonadales (83)**	**Pseudomonadaceae (83)**	**unclassified (<50)**
**OTU1212**	**Bacteria (100)**	**Actinobacteria (98)**	**Actinobacteria (98)**	**Pseudonocardiales (98)**	**Pseudonocardiaceae (75)**	**unclassified (<50)**
**OTU1213**	**Bacteria (100)**	**Proteobacteria (84)**	**Alphaproteobacteria (69)**	**unclassified (<50)**	**unclassified (<50)**	**unclassified (<50)**
**OTU1214**	**Bacteria (100)**	**Actinobacteria (98)**	**Actinobacteria (98)**	**Pseudonocardiales (97)**	**Pseudonocardiaceae (70)**	**unclassified (<50)**
**OTU1215**	**Bacteria (100)**	**Proteobacteria (100)**	**Gammaproteobacteria (100)**	**Vibrionales (100)**	**Vibrionaceae (100)**	***Allomonas* (54)**
**OTU1216**	**Bacteria**	**Actinobacteria (96)**	**Actinobacteria (96)**	**Pseudonocardiales (95)**	**Pseudonocardiaceae (76)**	**unclassified (<50)**
**OTU1217**	**Bacteria (100)**	**Proteobacteria (100)**	**Gammaproteobacteria (100)**	**Vibrionales (97)**	**Vibrionaceae (97)**	**unclassified (<50)**
**OTU1218**	**Bacteria (100)**	**Proteobacteria (100)**	**Gammaproteobacteria (99)**	**Vibrionales (74)**	**Vibrionaceae (74)**	**unclassified (<50)**
**OTU1219**	**Bacteria (100)**	**Proteobacteria (100)**	**Gammaproteobacteria (100)**	**Vibrionales (70)**	**Vibrionaceae (70)**	**unclassified (<50)**
**OTU1220**	**Bacteria (100)**	**Proteobacteria (100)**	**Gammaproteobacteria (100)**	**Pseudomonadales (100)**	**Pseudomonadaceae (100)**	***Pseudomonas* (95)**
**OTU1221**	**Bacteria (100)**	**Actinobacteria (100)**	**Actinobacteria (100)**	**Pseudonocardiales (100)**	**Pseudonocardiaceae (97)**	**unclassified (<50)**
**OTU1222**	**Bacteria**	**Planctomycetes (69)**	**Planctomycetia (69)**	**Planctomycetales (69)**	**Planctomycetaceae (69)**	**unclassified (<50)**
**OTU1223**	**Bacteria (100)**	**Proteobacteria (100)**	**Gammaproteobacteria (100)**	**Enterobacteriales (87)**	**Enterobacteriaceae (87)**	**unclassified (<50)**
**OTU1224**	**Bacteria (100)**	**Bacteroidetes (100)**	**Bacteroidia (100)**	**Bacteroidales (100)**	**Prevotellaceae (100)**	***Prevotella* (100)**

OTU1225	Bacteria (100)	Actinobacteria (100)	Actinobacteria (100)	Micrococcales (100)	Micrococcaceae (99)	*Nesterenkonia* (75)
OTU1226	Bacteria (100)	Proteobacteria (100)	Alphaproteobacteria (100)	Rhodobacterales (91)	Rhodobacteraceae (91)	*Roseicyclus* (50)
OTU1227	Bacteria (100)	Proteobacteria (100)	Alphaproteobacteria (100)	Rhodobacterales (95)	Rhodobacteraceae (95)	unclassified (<50)
OTU1228	Bacteria (100)	Actinobacteria (100)	Actinobacteria (100)	Pseudonocardiales (100)	Pseudonocardiaceae (90)	unclassified (<50)
OTU1229	Bacteria (100)	Proteobacteria (100)	Gammaproteobacteria (100)	Alteromonadales (78)	Pseudoalteromonadaceae (70)	*Pseudoalteromonas* (70)
OTU1230	Bacteria (100)	Chloroflexi (76)	Caldilineae (75)	Caldilineales (75)	Caldilineaceae (75)	*Litorilinea* (66)
OTU1231	Bacteria (100)	Proteobacteria (100)	Gammaproteobacteria (100)	Pseudomonadales (92)	Pseudomonadaceae (92)	unclassified (<50)
OTU1232	Bacteria (100)	Proteobacteria (100)	Gammaproteobacteria (100)	Vibrionales (100)	Vibrionaceae (100)	*Allomonas* (88)
OTU1233	Bacteria (100)	Proteobacteria (100)	Gammaproteobacteria (100)	Vibrionales (93)	Vibrionaceae (93)	unclassified (<50)
OTU1234	Bacteria (100)	Actinobacteria (100)	Actinobacteria (100)	Pseudonocardiales (100)	Pseudonocardiaceae (75)	unclassified (<50)
OTU1235	Bacteria (100)	Firmicutes (100)	Bacilli (93)	unclassified (<50)	unclassified (<50)	unclassified (<50)
OTU1236	Bacteria (100)	Proteobacteria (100)	Gammaproteobacteria (95)	Vibrionales (92)	Vibrionaceae (92)	*Allomonas* (83)
OTU1237	Bacteria (100)	Proteobacteria (100)	Gammaproteobacteria (100)	Pseudomonadales (93)	Pseudomonadaceae (93)	unclassified (<50)
OTU1238	Bacteria (100)	Proteobacteria (100)	Gammaproteobacteria (100)	Enterobacteriales (98)	Enterobacteriaceae (98)	unclassified (<50)
OTU1239	Bacteria (100)	Firmicutes (99)	Clostridia (99)	Clostridiales (99)	Lachnospiraceae (80)	*Cellulosilyticum* (78)
OUT1240	Bacteria (100)	Proteobacteria (100)	Gammaproteobacteria (100)	Aeromonadales (69)	Aeromonadaceae (69)	*Allomonas* (66)
OUT1241	Bacteria (100)	Actinobacteria (100)	Actinobacteria (100)	Micrococcales (100)	Micrococcaceae (93)	*Rothia* (90)
OUT1242	Bacteria (100)	Proteobacteria (100)	Gammaproteobacteria (100)	Vibrionales (100)	Vibrionaceae (100)	*Listonella* (76)
OUT1243	Bacteria (100)	Proteobacteria (100)	Gammaproteobacteria (100)	Enterobacteriales (95)	Enterobacteriaceae (95)	unclassified (<50)
OUT1244	Bacteria (100)	Proteobacteria (100)	Alphaproteobacteria (100)	Rhodobacterales (99)	Rhodobacteraceae (99)	unclassified (<50)
OUT1245	Bacteria (100)	Firmicutes (100)	Bacilli (100)	Bacillalles (100)	Listeriaceae (95)	*Brochothrix* (95)
OUT1246	Bacteria (100)	Proteobacteria (100)	Gammaproteobacteria (100)	Vibrionales (100)	Vibrionaceae (100)	*Listonella* (74)
OUT1247	Bacteria (100)	Planctomycets (100)	Planctomycetia (100)	Planctomycetales (100)	Planctomycetaceae (100)	*Pirellula* (70)
OUT1248	Bacteria (100)	Proteobacteria (100)	Gammaproteobacteria (100)	Vibrionales (88)	Vibrionaceae (88)	unclassified (<50)

**OTU1249**	**Bacteria (100)**	**Proteobacteria (100)**	**Gammaproteobacteria (100)**	**Alteromonadales (100)**	**Pseudoalteromonadaceae (97)**	***Pseudoalteromonas* (88)**
**OTU1250**	**Bacteria (100)**	**Actinobacteria (100)**	**Actinobacteria (100)**	**Pseudonocardiales (98)**	**Pseudonocardiaceae (72)**	**unclassified (<50)**
**OTU1251**	**Bacteria (100)**	**Actinobacteria (100)**	**Actinobacteria (100)**	**Pseudonocardiales (100)**	**Pseudonocardiaceae (82)**	**unclassified (<50)**
**OTU1252**	**Bacteria (100)**	**Actinobacteria (100)**	**Actinobacteria (100)**	**Pseudonocardiales (100)**	**Pseudonocardiaceae (100)**	**unclassified (<50)**
**OTU1253**	**Bacteria (100)**	**Proteobacteria (100)**	**Alphaproteobacteria (100)**	**Rhodospirillales (99)**	**Rhodospirillaceae (97)**	***Azospirillum* (95)**
**OTU1254**	**Bacteria (100)**	**Actinobacteria (100)**	**Actinobacteria (100)**	**Pseudonocardiales (100)**	**Pseudonocardiaceae (85)**	**unclassified (<50)**
**OTU1255**	**Bacteria (100)**	**unclassified (<50)**	**unclassified (<50)**	**unclassified (<50)**	**unclassified (<50)**	**unclassified (<50)**
**OTU1256**	**Bacteria (100)**	**Proteobacteria (100)**	**Gammaproteobacteria (100)**	**Vibrionales (98)**	**Vibrionaceae (98)**	***Salinivibrio* (54)**
**OTU1257**	**Bacteria (100)**	**Proteobacteria (100)**	**Gammaproteobacteria (100)**	**Aeromonadales (72)**	**Aeromonadaceae (72)**	***Aeromonas* (54)**
**OTU1258**	**Bacteria (100)**	**Proteobacteria (99)**	**Gammaproteobacteria (98**	**Pseudomonadales (93)**	**Pseudomonadaceae (93)**	***Azomonas* (62)**
**OTU1259**	**Bacteria (100)**	**Proteobacteria (100)**	**Gammaproteobacteria (100)**	**Vibrionales (99)**	**Vibrionaceae (99)**	***Allomonas* (61)**
**OTU1260**	**Bacteria (100)**	**Actinobacteria (100)**	**Actinobacteria (100)**	**Pseudonocardiales (99)**	**Pseudonocardiaceae (88)**	**unclassified (<50)**
**OTU1261**	**Bacteria (100)**	**Proteobacteria (100)**	**Gammaproteobacteria (100)**	**Vibrionales (100)**	**Vibrionaceae (100)**	**unclassified (<50)**
**OTU1262**	**Bacteria (100)**	**Proteobacteria (100)**	**Gammaproteobacteria (100)**	**Vibrionales (100)**	**Vibrionaceae (100)**	***Allomonas* (65)**
**OTU1263**	**Bacteria (100)**	**Proteobacteria (100)**	**Alphaproteobacteria (100)**	**Rhodobacterales (96)**	**Rhodobacteraceae (96)**	***Thalassobius* (58)**
**OTU1264**	**Bacteria (100)**	**Actinobacteria (100)**	**Actinobacteria (100)**	**Pseudonocardiales (97)**	**Pseudonocardiaceae (88)**	**unclassified (<50)**
**OTU1265**	**Bacteria (100)**	**Proteobacteria (100)**	**Gammaproteobacteria (100)**	**Alteromonadales (100)**	**Shewanellaceae (100)**	***Shewanella* (100)**
**OTU1266**	**Bacteria**	**Actinobacteria (96)**	**Actinobacteria (96)**	**Pseudonocardiales (95)**	**Pseudonocardiaceae (74)**	**unclassified (<50)**
**OTU1267**	**Bacteria (100)**	**Actinobacteria (100)**	**Actinobacteria (100)**	**Pseudonocardiales (100)**	**Pseudonocardiaceae (80)**	**unclassified (<50)**
**OTU1268**	**Bacteria (100)**	**Proteobacteria (100)**	**Gammaproteobacteria (100)**	**Oceanospirillales (95)**	**Alcanivoracaceae (94)**	***Alcanivorax* (94)**
**OTU1269**	**Bacteria (100)**	**Proteobacteria (99)**	**Betaproteobacteria (82**	**Neisseriales (78)**	**Neisseriaceae (78)**	**unclassified (<50)**
**OTU1270**	**Bacteria (100)**	**Proteobacteria (100)**	**Gammaproteobacteria (100)**	**Vibrionales (100)**	**Vibrionaceae (100)**	***Listonella* (88)**
**OTU1271**	**Bacteria (100)**	**Proteobacteria (99)**	**Gammaproteobacteria (99)**	**Pseudomonadales (85)**	**Pseudomonadaceae (85)**	***Pseudomonas* (54)**
**OTU1272**	**Bacteria (100)**	**Proteobacteria (100)**	**Gammaproteobacteria (100)**	**Vibrionales (99)**	**Vibrionaceae (99)**	***Listonella* (84)**

OTU1273	Bacteria (100)	Proteobacteria (100)	Gammaproteobacteria (100)	Vibrionales (90)	Vibrionaceae (90)	unclassified (<50)
OTU1274	Bacteria (100)	Proteobacteria (100)	Alphaproteobacteria (100)	Rhodobacterales (97)	Rhodobacteraceae (97)	unclassified (<50)
OTU1275	Bacteria (100)	Proteobacteria (99)	Gammaproteobacteria (99)	Oceanospirillales (54)	unclassified (<50)	unclassified (<50)
OTU1276	Bacteria (100)	Proteobacteria (100)	Gammaproteobacteria (100)	Vibrionales (100)	Vibrionaceae (100)	unclassified (<50)
OTU1277	Bacteria (100)	Proteobacteria (100)	Gammaproteobacteria (100)	Vibrionales (100)	Vibrionaceae (100)	*Listonella* (90)
OTU1278	Bacteria (100)	Deinococcus-Thermus (100)	Deinococci (100)	Thermales (100)	Thermaceae (100)	*Thermus* (100)
OTU1279	Bacteria (100)	Proteobacteria (98)	Gammaproteobacteria (96)	Pseudomonadales (94)	Moraxellaceae (94)	*Acinetobacter* (70)
OTU1280	Bacteria (100)	Proteobacteria (100)	Gammaproteobacteria (100)	Vibrionales (100)	Vibrionaceae (100)	*Lucibacterium* (82)
OTU1281	Bacteria (100)	Actinobacteria (100)	Actinobacteria (100)	Pseudonocardiales (100)	Pseudonocardiaceae (99)	unclassified (<50)
OTU1282	Bacteria (100)	Proteobacteria (100)	Gammaproteobacteria (100)	Vibrionales (100)	Vibrionaceae (100)	*Allomonas* (75)
OTU1283	Bacteria (100)	Actinobacteria (99)	Actinobacteria (99)	Pseudonocardiales (99)	Pseudonocardiaceae (87)	unclassified (<50)
OTU1284	Bacteria (100)	Firmicutes (100)	Bacilli (100)	Lactobacillales (100)	Leuconostocaceae (100)	*Leuconostoc* (100)
OTU1285	Bacteria (100)	Proteobacteria (100)	Alphaproteobacteria (100)	Rhodobacterales (97)	Rhodobacteraceae (97)	unclassified (<50)
OTU1286	Bacteria (100)	Proteobacteria (100)	Gammaproteobacteria (100)	Vibrionales (100)	Vibrionaceae (100)	*Allomonas* (59)
OTU1287	Bacteria (100)	Firmicutes (99)	Bacilli (98)	Bacillales (91)	Staphylococcaceae (83)	*Staphylococcus* (60)
OTU1288	Bacteria (100)	Proteobacteria (100)	Gammaproteobacteria (100)	Vibrionales (100)	Vibrionaceae (100)	*Allomonas* (66)
OTU1289	Bacteria (100)	Proteobacteria (100)	Gammaproteobacteria (100)	Pseudomonadales (97)	Pseudomonadaceae (97)	*Pseudomonas* (69)
OTU1290	Bacteria (100)	Proteobacteria (100)	Gammaproteobacteria (100)	Vibrionales (100)	Vibrionaceae (100)	*Vibrio* (63)
OTU1291	Bacteria (100)	Proteobacteria (100)	Gammaproteobacteria (100)	Vibrionales (100)	Vibrionaceae (100)	*Allomonas* (57)
OTU1292	Bacteria (100)	Proteobacteria (100)	Gammaproteobacteria (100)	Vibrionales (100)	Vibrionaceae (100)	*Allomonas* (80)
OTU1293	Bacteria (100)	Proteobacteria (100)	Gammaproteobacteria (100)	Vibrionales (100)	Vibrionaceae (100)	*Allomonas* (64)
OTU1294	Bacteria (100)	Proteobacteria (100)	Gammaproteobacteria (100)	Pseudomonadales (93)	Moraxellaceae (93)	*Acinetobacter* (92)
OTU1295	Bacteria (100)	Firmicutes (100)	Bacilli (100)	Bacillalles (100)	Listeriaceae (92)	*Brochothrix* (92)
OTU1296	Bacteria (100)	Firmicutes (99)	Bacilli (99)	Lactobacillales (95)	Lactobacillaceae (68)	*Lactobacillus* (57)

**OTU1297**	**Bacteria (100)**	**Proteobacteria (100)**	**Gammaproteobacteria (100)**	**Vibrionales (100)**	**Vibrionaceae (100)**	**unclassified (<50)**
**OTU1298**	**Bacteria (100)**	**Firmicutes (100)**	**Bacilli (100)**	**Bacillales (97)**	**unclassified (<50)**	**unclassified (<50)**
**OTU1299**	**Bacteria (100)**	**Actinobacteria (100)**	**Actinobacteria (100)**	**Pseudonocardiales (100)**	**Pseudonocardiaceae (82)**	**unclassified (<50)**
**OTU1300**	**Bacteria (100)**	**Actinobacteria (100)**	**Actinobacteria (100)**	**Pseudonocardiales (99)**	**Pseudonocardiaceae (95)**	**unclassified (<50)**
**OTU1301**	**Bacteria (95)**	**unclassified (<50)**	**unclassified (<50)**	**unclassified (<50)**	**unclassified (<50)**	**unclassified (<50)**
**OTU1302**	**Bacteria (99)**	**unclassified (<50)**	**unclassified (<50)**	**unclassified (<50)**	**unclassified (<50)**	**unclassified (<50)**
**OTU1303**	**Bacteria (100)**	**Bacteroidetes (98)**	**Sphingobacteria (90)**	**Sphingobacteriales (90)**	**Saprospiraceae (86)**	**unclassified (<50)**
**OTU1304**	**Bacteria (100)**	**Actinobacteria (98)**	**Actinobacteria (98)**	**Pseudonocardiales (98)**	**Pseudonocardiaceae (83)**	**unclassified (<50)**
**OTU1305**	**Bacteria (100)**	**Proteobacteria (100)**	**Alphaproteobacteria (100)**	**Rhodobacterales (59)**	**Rhodobacteraceae (59)**	**unclassified (<50)**
**OTU1306**	**Bacteria (100)**	**Proteobacteria (100)**	**Gammaproteobacteria (100)**	**Vibrionales (100)**	**Vibrionaceae (100)**	***Allomonas* (62)**
**OTU1307**	**Bacteria (100)**	**Proteobacteria (100)**	**Gammaproteobacteria (100)**	**Oceanospirillales (100)**	**Oceanospirillaceae (100)**	***Marinomonas* (100)**
**OTU1308**	**Bacteria (100)**	**Proteobacteria (100)**	**Alphaproteobacteria (100)**	**Rhodobacterales (87)**	**Rhodobacteraceae (87)**	***Roseicyclus* (51)**
**OTU1309**	**Bacteria (100)**	**Proteobacteria (100)**	**Gammaproteobacteria (100)**	**Vibrionales (99)**	**Vibrionaceae (99)**	***Allomonas* (87)**
**OTU1310**	**Bacteria (100)**	**Proteobacteria (100)**	**Gammaproteobacteria (87)**	**Vibrionales (86)**	**Vibrionaceae (86)**	***Allomonas* (76)**
**OTU1311**	**Bacteria (100)**	**Proteobacteria (100)**	**Gammaproteobacteria (99)**	**Aeromonadales (75)**	**Aeromonadaceae (75)**	***Aeromonas* (65)**
**OTU1312**	**Bacteria (100)**	**Actinobacteria (100)**	**Actinobacteria (100)**	**Pseudonocardiales (98)**	**Pseudonocardiaceae (78)**	**unclassified (<50)**
**OTU1313**	**Bacteria (100)**	**Proteobacteria (100)**	**Gammaproteobacteria (100)**	**Vibrionales (100)**	**Vibrionaceae (100)**	***Allomonas* (57)**
**OTU1314**	**Bacteria (100)**	**Proteobacteria (100)**	**Gammaproteobacteria (100)**	**Vibrionales (98)**	**Vibrionaceae (98)**	**unclassified (<50)**
**OTU1315**	**Bacteria (100)**	**Bacteroidetes (100)**	**Flavobacteria (100)**	**Flavobacteriales (100)**	**Flavobacteriaceae (100)**	***Cloacibacterium* (100)**
**OTU1316**	**Bacteria (100)**	**Planctomycets (51)**	**Planctomycetia (51)**	**unclassified (<50)**	**unclassified (<50)**	**unclassified (<50)**
**OTU1317**	**Bacteria (100)**	**Proteobacteria (100)**	**Gammaproteobacteria (100)**	**Vibrionales (100)**	**Vibrionaceae (100)**	***Allomonas* (70)**
**OTU1318**	**Bacteria (100)**	**Proteobacteria (100)**	**Gammaproteobacteria (100)**	**Vibrionales (80)**	**Vibrionaceae (80)**	**unclassified (<50)**
**OUT1319**	**Bacteria (100)**	**Proteobacteria (100)**	**Gammaproteobacteria (100)**	**Pseudomonadales (100)**	**Moraxellaceae (100)**	***Acinetobacter* (100)**
**OUT1320**	**Bacteria (100)**	**Proteobacteria (100)**	**Gammaproteobacteria (100)**	**Vibrionales (100)**	**Vibrionaceae (100)**	***Listonella* (77)**

**OUT1321**	**Bacteria (100)**	**Proteobacteria (100)**	**Gammaproteobacteria (100)**	**Alteromonadales (100)**	**Shewanellaceae (100)**	***Shewanella* (100)**
**OUT1322**	**Bacteria (100)**	**Actinobacteria (99)**	**Actinobacteria (99)**	**Pseudonocardiales (98)**	**Pseudonocardiaceae (79)**	**unclassified (<50)**
**OUT1323**	**Bacteria (100)**	**Actinobacteria (100)**	**Actinobacteria (100)**	**Pseudonocardiales (100)**	**Pseudonocardiaceae (80)**	**unclassified (<50)**
**OUT1324**	**Bacteria (100)**	**Actinobacteria (100)**	**Actinobacteria (100)**	**Pseudonocardiales (100)**	**Pseudonocardiaceae (97)**	**unclassified (<50)**
**OUT1325**	**Bacteria (100)**	**Actinobacteria (100)**	**Actinobacteria (100)**	**Pseudonocardiales (100)**	**unclassified (<50)**	**unclassified (<50)**
**OUT1326**	**Bacteria (100)**	**Proteobacteria (100)**	**Gammaproteobacteria (100)**	**Vibrionales (100)**	**Vibrionaceae (100)**	***Allomonas* (77)**
**OUT1327**	**Bacteria (100)**	**Proteobacteria (100)**	**Alphaproteobacteria (100)**	**Rhodobacterales (99)**	**Rhodobacteraceae (99)**	**unclassified (<50)**
**OUT1328**	**Bacteria (100)**	**Proteobacteria (100)**	**Gammaproteobacteria (100)**	**Vibrionales (100)**	**Vibrionaceae (100)**	***Vibrio* (68)**
**OUT1329**	**Bacteria (100)**	**Proteobacteria (100)**	**Alphaproteobacteria (100)**	**Rhodospirillales (100)**	**Rhodospirillaceae (98)**	***Azospirillum* (86)**
**OUT1330**	**Bacteria (100)**	**Proteobacteria (100)**	**Gammaproteobacteria (100)**	**Vibrionales (100)**	**Vibrionaceae (100)**	***Lucibacterium* (52)**
**OUT1331**	**Bacteria (100)**	**Proteobacteria (100)**	**Gammaproteobacteria (100)**	**Vibrionales (100)**	**Vibrionaceae (100)**	***Allomonas* (81)**
**OUT1332**	**Bacteria (100)**	**Firmicutes (99)**	**Bacilli (90)**	**Bacillales (82)**	**Staphylococcaceae (67)**	***Staphylococcus* (50)**
**OUT1333**	**Bacteria (100)**	**Proteobacteria (100)**	**Gammaproteobacteria (100)**	**Aeromonadales (100)**	**Aeromonadaceae (100)**	***Aeromonas* (100)**
**OUT1334**	**Bacteria (100)**	**Proteobacteria (100)**	**Gammaproteobacteria (100)**	**Vibrionales (90)**	**Vibrionaceae (90)**	**unclassified (<50)**
**OUT1335**	**Bacteria (100)**	**Proteobacteria (100)**	**Gammaproteobacteria (95)**	**Alteromonadales (93)**	**Pseudoalteromonadaceae (83)**	***Pseudoalteromonas* (63)**
**OUT1336**	**Bacteria (100)**	**Proteobacteria (100)**	**Gammaproteobacteria (100)**	**Vibrionales (100)**	**Vibrionaceae (100)**	***Vibrio* (63)**
**OUT1337**	**Bacteria (100)**	**Proteobacteria (100)**	**Gammaproteobacteria (100)**	**Vibrionales (100)**	**Vibrionaceae (100)**	***Listonella* (62)**
**OUT1338**	**Bacteria (100)**	**Actinobacteria (80)**	**Actinobacteria (80)**	**Pseudonocardiales (80)**	**Pseudonocardiaceae (67)**	**unclassified (<50)**
**OUT1339**	**Bacteria (100)**	**Proteobacteria (100)**	**Gammaproteobacteria (100)**	**Vibrionales (100)**	**Vibrionaceae (100)**	**unclassified (<50)**
**OUT1340**	**Bacteria (100)**	**Proteobacteria (100)**	**Gammaproteobacteria (100)**	**Vibrionales (97)**	**Vibrionaceae (97)**	***Allomonas* (78)**
**OUT1341**	**Bacteria (100)**	**Actinobacteria (80)**	**Actinobacteria (80)**	**Pseudonocardiales (80)**	**Pseudonocardiaceae (70)**	**unclassified (<50)**
**OUT1342**	**Bacteria (100)**	**Proteobacteria (100)**	**Gammaproteobacteria (100)**	**Enterobacteriales (100)**	**Enterobacteriaceae (100)**	***Escherichia/Shigella* (98)**
**OTU1343**	**Bacteria (100)**	**Proteobacteria (100)**	**Gammaproteobacteria (100)**	**Pseudomonadales (100)**	**Moraxellaceae (100)**	***Acinetobacter* (100)**
**OTU1344**	**Bacteria**	**Proteobacteria (99)**	**Gammaproteobacteria (99)**	**Vibrionales (76)**	**Vibrionaceae (76)**	**unclassified (<50)**

**OTU1345**	**Bacteria (100)**	**Proteobacteria (100)**	**Gammaproteobacteria (100)**	**Vibrionales (100)**	**Vibrionaceae (100)**	***Allomonas* (78)**
**OTU1346**	**Bacteria (100)**	**Firmicutes (100)**	**Clostridia (100)**	**Clostridiales (100)**	**Clostridiales_Incertae Sedis XI (100)**	***Peptoniphilus* (100)**
**OTU1347**	**Bacteria (100)**	**Proteobacteria (100)**	**Gammaproteobacteria (100)**	**Vibrionales (100)**	**Vibrionaceae (100)**	***Allomonas* (74)**
**OTU1348**	**Bacteria (100)**	**Proteobacteria (100)**	**Gammaproteobacteria (100)**	**Vibrionales (97)**	**Vibrionaceae (97)**	**unclassified (<50)**
**OTU1349**	**Bacteria (100)**	**Proteobacteria (100)**	**Gammaproteobacteria (100)**	**Aeromonadales (99)**	**Aeromonadaceae (99)**	***Aeromonas* (99)**
**OTU1350**	**Bacteria (100)**	**Proteobacteria (100)**	**Gammaproteobacteria (100)**	**Pseudomonadales (99)**	**Moraxellaceae (99)**	***Acinetobacter* (97)**
**OTU1351**	**Bacteria (100)**	**Proteobacteria (100)**	**Gammaproteobacteria (100)**	**Vibrionales (100)**	**Vibrionaceae (100)**	**unclassified (<50)**
**OTU1352**	**Bacteria (100)**	**Actinobacteria (100)**	**Actinobacteria (100)**	**Pseudonocardiales (100)**	**unclassified (<50)**	**unclassified (<50)**
**OTU1353**	**Bacteria (100)**	**Proteobacteria (100)**	**Gammaproteobacteria (100)**	**Vibrionales (100)**	**Vibrionaceae (100)**	***Allomonas* (52)**
**OTU1354**	**Bacteria (100)**	**Bacteroidetes (97)**	**Sphingobacteria (90)**	**Sphingobacteriales (90)**	**Saprospiraceae (79)**	***Haliscomenobacter* (75)**
**OTU1355**	**Bacteria (100)**	**Proteobacteria (100)**	**Gammaproteobacteria (100)**	**Vibrionales (100)**	**Vibrionaceae (100)**	***Lucibacterium* (69)**
**OTU1356**	**Bacteria (100)**	**Proteobacteria (100)**	**Gammaproteobacteria (100)**	**Vibrionales (90)**	**Vibrionaceae (90)**	***Allomonas* (66)**
**OTU1357**	**Bacteria (100)**	**Proteobacteria (100)**	**Gammaproteobacteria (100)**	**Vibrionales (100)**	**Vibrionaceae (100)**	***Lucibacterium* (76)**
**OTU1358**	**Bacteria (99)**	**unclassified (<50)**	**unclassified (<50)**	**unclassified (<50)**	**unclassified (<50)**	**unclassified (<50)**
**OTU1359**	**Bacteria (100)**	**Actinobacteria (100)**	**Actinobacteria (100)**	**Pseudonocardiales (100)**	**Pseudonocardiaceae (83)**	**unclassified (<50)**
**OTU1360**	**Bacteria (100)**	**Proteobacteria (100)**	**Gammaproteobacteria (100)**	**Pseudomonadales (100)**	**Moraxellaceae (100)**	***Acinetobacter* (99)**
**OTU1361**	**Bacteria (100)**	**Proteobacteria (100)**	**Gammaproteobacteria (100)**	**Vibrionales (100)**	**Vibrionaceae (100)**	***Allomonas* (76)**
**OTU1362**	**Bacteria (100)**	**Proteobacteria (99)**	**Gammaproteobacteria (98)**	**Vibrionales (97)**	**Vibrionaceae (97)**	***Allomonas* (86)**
**OTU1363**	**Bacteria (100)**	**Proteobacteria (100)**	**Gammaproteobacteria (100)**	**Vibrionales (100)**	**Vibrionaceae (100)**	***Allomonas* (70)**
**OTU1364**	**Bacteria (100)**	**Actinobacteria (100)**	**Actinobacteria (100)**	**Pseudonocardiales (100)**	**Pseudonocardiaceae (94)**	**unclassified (<50)**
**OTU1365**	**Bacteria (100)**	**Proteobacteria (100)**	**Gammaproteobacteria (100)**	**Vibrionales (99)**	**Vibrionaceae (99)**	**unclassified (<50)**
**OTU1366**	**Bacteria (100)**	**Proteobacteria (100)**	**Gammaproteobacteria (100)**	**Enterobacteriales (100)**	**Enterobacteriaceae (100)**	**unclassified (<50)**
**OUT1367**	**Bacteria (100)**	**Actinobacteria (100)**	**Actinobacteria (100)**	**Pseudonocardiales (100)**	**Pseudonocardiaceae (99)**	**unclassified (<50)**
**OUT1368**	**Bacteria (100)**	**Proteobacteria (100)**	**Gammaproteobacteria (100)**	**Vibrionales (99)**	**Vibrionaceae (99)**	***Allomonas* (57)**

**OUT1369**	**Bacteria (100)**	**Actinobacteria (84)**	**Actinobacteria (84)**	**Acidimicrobiales (51)**	**Acidimicrobiaceae (51)**	**unclassified (<50)**
**OUT1370**	**Bacteria (100)**	**Proteobacteria (100)**	**Gammaproteobacteria (100)**	**Aeromonadales (97)**	**Aeromonadaceae (97)**	***Aeromonas* (94)**
**OUT1371**	**Bacteria (100)**	**Actinobacteria (100)**	**Actinobacteria (100)**	**Pseudonocardiales (100)**	**Pseudonocardiaceae (88)**	**unclassified (<50)**
**OUT1372**	**Bacteria (100)**	**Actinobacteria (100)**	**Actinobacteria (100)**	**Pseudonocardiales (100)**	**Pseudonocardiaceae (84)**	**unclassified (<50)**
**OUT1373**	**Bacteria (100)**	**Proteobacteria (100)**	**Gammaproteobacteria (100)**	**Vibrionales (73)**	**Vibrionaceae (73)**	**unclassified (<50)**
**OUT1374**	**Bacteria (100)**	**Actinobacteria (100)**	**Actinobacteria (100)**	**Pseudonocardiales (100)**	**Pseudonocardiaceae (89)**	**unclassified (<50)**
**OUT1375**	**Bacteria (100)**	**Proteobacteria (100)**	**Gammaproteobacteria (100)**	**Alteromonadales (100)**	**Pseudoalteromonadaceae (96)**	***Pseudoalteromonas* (96)**
**OUT1376**	**Bacteria (100)**	**Proteobacteria (100)**	**Gammaproteobacteria (100)**	**Vibrionales (99)**	**Vibrionaceae (99)**	***Listonella* (60)**
**OUT1377**	**Bacteria (100)**	**Proteobacteria (100)**	**Gammaproteobacteria (100)**	**Vibrionales (100)**	**Vibrionaceae (100)**	***Allomonas* (76)**
**OUT1378**	**Bacteria (100)**	**Proteobacteria (100)**	**Alphaproteobacteria (100)**	**Rhodobacterales (98)**	**Rhodobacteraceae (98)**	**unclassified (<50)**
**OUT1379**	**Bacteria (99)**	**Proteobacteria (78)**	**unclassified (<50)**	**unclassified (<50)**	**unclassified (<50)**	**unclassified (<50)**
**OUT1380**	**Bacteria (100)**	**Actinobacteria (100)**	**Actinobacteria (100)**	**Pseudonocardiales (97)**	**Pseudonocardiaceae (78)**	**unclassified (<50)**
**OUT1381**	**Bacteria (100)**	**Proteobacteria (100)**	**Gammaproteobacteria (100)**	**Vibrionales (74)**	**Vibrionaceae (74)**	**unclassified (<50)**
**OUT1382**	**Bacteria (100)**	**Actinobacteria (99)**	**Actinobacteria (99)**	**Pseudonocardiales (98)**	**Pseudonocardiaceae (77)**	**unclassified (<50)**
**OUT1383**	**Bacteria (100)**	**Actinobacteria (100)**	**Actinobacteria (100)**	**Pseudonocardiales (100)**	**Pseudonocardiaceae (86)**	**unclassified (<50)**
**OUT1384**	**Bacteria (100)**	**Proteobacteria (100)**	**Gammaproteobacteria (100)**	**Vibrionales (100)**	**Vibrionaceae (100)**	***Listonella* (82)**
**OUT1385**	**Bacteria (100)**	**Proteobacteria (100)**	**Gammaproteobacteria (100)**	**Pseudomonadales (98)**	**Pseudomonadaceae (98)**	***Pseudomonas* (70)**
**OUT1386**	**Bacteria (100)**	**Proteobacteria (100)**	**Gammaproteobacteria (100)**	**Vibrionales (100)**	**Vibrionaceae (100)**	***Vibrio* (84)**
**OUT1387**	**Bacteria (100)**	**Proteobacteria (100)**	**Gammaproteobacteria (100)**	**Vibrionales (100)**	**Vibrionaceae (100)**	**unclassified (<50)**
**OUT1388**	**Bacteria (100)**	**Proteobacteria (100)**	**Gammaproteobacteria (100)**	**Vibrionales (100)**	**Vibrionaceae (100)**	***Allomonas* (68)**
**OUT1389**	**Bacteria (100)**	**Proteobacteria (100)**	**Gammaproteobacteria (99)**	**Vibrionales (97)**	**Vibrionaceae (97)**	***Allomonas* (53)**
**OUT1390**	**Bacteria (100)**	**Proteobacteria (100)**	**Gammaproteobacteria (100)**	**Pseudomonadales (100)**	**Moraxellaceae (100)**	***Acinetobacter* (100)**

**OTU1391**	**Bacteria (100)**	**Proteobacteria (100)**	**Gammaproteobacteria (100)**	**Vibrionales (99)**	**Vibrionaceae (99)**	**unclassified (<50)**
**OTU1392**	**Bacteria (100)**	**Actinobacteria (98)**	**Actinobacteria (98)**	**Pseudonocardiales (96)**	**unclassified (<50)**	**unclassified (<50)**
**OTU1393**	**Bacteria (100)**	**Proteobacteria (100)**	**Alphaproteobacteria (100)**	**Rhodobacterales (70)**	**Rhodobacteraceae (70)**	**unclassified (<50)**
**OTU1394**	**Bacteria (100)**	**Actinobacteria (100)**	**Actinobacteria (100)**	**Pseudonocardiales (100)**	**Pseudonocardiaceae (92)**	**unclassified (<50)**

Table S2. BLAST homologies for the OTUs found at least five times

OTUs	Best BLAST homology	Entry	%	sample
OTU0001	Uncultured *Saccharopolyspora* sp. clone VE21-K19 16S ribosomal RNA gene, partial sequence	KF767307	99	parchment
OTU0002	*Saccharopolyspora* sp. AFM 10238 16S ribosomal RNA gene, partial sequence	KF673492	98	Dead Sea
OTU0003	Uncultured bacterium clone I3Q1XXJ02CAUFP 16S ribosomal RNA gene, partial sequence	KP952426	99	intestinal flora of black tiger shrimp
OTU0004	*Saccharopolyspora* sp. 13-18-42 16S ribosomal RNA gene, partial sequence	KM886189	99	sponge, South China Sea
OTU0005	*Pseudoalteromonas* sp. H6 16S ribosomal RNA gene, partial sequence	KX236489	99	seawater
OTU0006	Uncultured bacterium clone HYPJBHH15I4RDY 16S ribosomal RNA gene, partial sequence	KF316172	99	hydraulic fracturing fluids and produced water from shale gas extraction
OTU0007	*Acinetobacter* sp. G2-5 partial 16S rRNA gene, isolate G2-5	LK054569	99	anchihaline waters, Western Mediterranean coastal cave
OTU0008	*Aeromonas veronii* strain CB51, complete genome	CP015448	99	blood
OTU0009	Uncultured bacterium clone I3Q1XXJ02BWIF9 16S ribosomal RNA gene, partial sequence	KP953689	99	intestinal flora of black tiger shrimp
OTU0010	Uncultured bacterium clone I3Q1XXJ02BTJK5 16S ribosomal RNA gene, partial sequence	KP952131	99	intestinal flora of black tiger shrimp
OTU0011	*Acinetobacter pittii* strain IITRC19 16S ribosomal RNA gene, partial sequence	KU715842	99	pulp paper mill discharged sludge
OTU0012	*Staphylococcus aureus* strain ST20130938, complete genome	CP012978	99	hip prosthesis infection
OTU0013	*Pseudomonas* sp. G3-45 partial 16S rRNA gene, isolate G3-45	LK054584	99	anchihaline waters, Western Mediterranean coastal cave
OTU0014	Uncultured bacterium clone nbw525c03c1 16S ribosomal RNA	GQ105040	99	skin, volar forearm

	gene, partial sequence			
OTU0015	Uncultured Pseudonocardiaceae bacterium partial 16S rRNA gene, clone IZ35.2_K11	FN689586	99	fragment of paper bulls of indulgence of the 15th-16th century
OTU0016	*Pantoea agglomerans* strain XF1 16S ribosomal RNA gene, partial sequence	KU373053	99	sugar industry
OTU0017	*Phyllobacterium* sp. JCM 28305 gene for 16S ribosomal RNA, partial	LC133652	99	stone chambers of the Takamatsuzuka and Kitora Tumuli
OTU0018	*Enterococcus hirae* strain RCB498 16S ribosomal RNA gene, partial sequence	KT260710	100	bat guano
OTU0019	Uncultured *Saccharopolyspora* sp. clone VE21-K36 16S ribosomal RNA gene, partial sequence	KF767309	99	parchment
OTU0020	Uncultured Corynebacterineae bacterium clone 5788 16S ribosomal RNA gene, partial sequence	KP106988	100	fecal sample
OTU0021	Uncultured bacterium clone G4TICC403GDU7W 16S ribosomal RNA gene, partial sequence	JQ074296	99	indoor air
OTU0022	Uncultured bacterium clone G7DUZBG01BCKNP 16S ribosomal RNA gene, partial sequence	JX940887	99	intestinal flora of black tiger shrimp
OTU0023	*Bacillus amyloliquefaciens* strain CH-04 16S ribosomal RNA gene, partial sequence	KU352764	99	coastal marine water, Tamil Nadu, India
OTU0024	*Escherichia coli* strain RCB930 16S ribosomal RNA gene, partial sequence	KT261142	100	bat guano
OTU0025	Uncultured bacterium clone GXTJ5A301AX3JO 16S ribosomal RNA gene, partial sequence	KF332701	99	intestinal flora of black tiger shrimp
OTU0026	Uncultured *Saccharopolyspora* sp. clone VE21-K19 16S ribosomal RNA gene, partial sequence	KF767307	98	parchment
OTU0027	Uncultured bacterium clone OTU_23810 16S ribosomal RNA gene,	KR855094	99	soil of a tobacco plantation

	partial sequence			
OTU0028	*Saccharopolyspora qijiaojingensis* strain YIM 91168 16S ribosomal RNA gene, partial sequence	NR_116107	99	salt lake
OTU0029	Uncultured bacterium clone I3Q1XXJ01BC414 16S ribosomal RNA gene, partial sequence	KP953369	99	intestinal flora of black tiger shrimp
OTU0030	*Vibrio ponticus* strain SB6 16S ribosomal RNA, partial sequence	KU249214	99	fish
OTU0031	Uncultured bacterium clone OTU_81 16S ribosomal RNA gene, partial sequence	KT360371	99	Pu-erh tea
OTU0032	*Acinetobacter tjernbergiae* strain T40 16S ribosomal RNA gene, partial sequence	KU991570	100	surface water
OTU0033	Uncultured bacterium clone nbw14e10c1 16S ribosomal RNA gene, partial sequence	GQ060210	99	human skin, external auditory canal
OTU0034	*Pseudomonas indoloxydans* strain Bss-13a 16S ribosomal RNA gene	KX161387	99	halophilic and halotolerant bacteria from Badab-e Surt travertine spring
OTU0035	*Alteromonas confluentis* strain DSSK2-12 16S ribosomal RNA, partial sequence	NR_137375	99	water from the junction between the ocean and a freshwater spring
OTU0036	Uncultured bacterium clone nbw212d06c1 16S ribosomal RNA gene partial sequence	GQ073741	98	skin microbiome
OTU0037	*Pseudomonas stutzeri* strain AAU PG1 16S ribosomal RNA gene, partial	KX358068	99	organic concoction "panchagavya"
OTU0039	Uncultured *Photobacterium* sp. clone F.c-7 16S ribosomal RNA gene, partial sequence	GU225837	99	shrimp intestine
OTU0040	*Massilia timonae* strain Bsw-26b 16S ribosomal RNA gene, partial sequence	KX161400	99	water, Badab-e Surt travertine spring

OTU0041	Uncultured bacterium clone G4TICC403GDU7W 16S ribosomal RNA gene, partial sequence	JQ074296	98	Indoor air
OTU0042	*Streptococcus vestibularis* strain MRS_87-5 16S ribosomal RNA gene, partial sequence	KX673987	99	human fecal sample
OTU0043	*Marinomonas ushuaiensis* strain hMe9-8 16S ribosomal RNA gene, partial sequence	KX453227	99	fluids of marine invertebrates
OTU0044	Uncultured bacterium clone HH_b02_1 16S ribosomal RNA gene, partial sequence	EU775648	100	hedgehog faeces
OTU0045	*Finegoldia magna* strain DNF00923 16S ribosomal RNA gene, partial sequence	KU726690	99	vaginal fluid from subject with bacterial vaginosis
OTU0046	*Micrococcus* sp. strain BAB-5964 16S ribosomal RNA gene, partial sequence	KX622627	99	soil
OTU0047	*Saccharopolyspora halotolerans* strain TRM 45123 16S ribosomal RNA, partial sequence	NR_134166	98	hypersaline lake
OTU0048	Uncultured bacterium clone p23_20-D03 16S ribosomal RNA gene, partial sequence	KX437175	100	plaque from teeth
OTU0049	Uncultured bacterium clone I3Q1XXJ02BS9EH 16S ribosomal RNA gene, partial sequence	KP953699	100	intestinal flora of black tiger shrimp
OTU0050	*Corynebacterium variabile* strain AA6-6 16S ribosomal RNA gene, partial sequence	KU663670	99	insect gut microflora
OTU0051	Uncultured *Saccharopolyspora* sp. clone VE21-K11 16S ribosomal RNA gene, partial sequence	KF767304	99	parchment
OTU0052	*Pseudoalteromonas marina* strain NJES-88 16S ribosomal RNA gene, partial sequence	KR140261	99	digestive tracts of Antarctic krills
OTU0053	Uncultured bacterium clone IZ1RPV403DLWZX 16S ribosomal RNA	KP948142	99	intestinal flora of black tiger shrimp

	gene, partial sequence			
OTU0054	*Saccharopolyspora cavernae* strain YIM C01235 16S ribosomal RNA gene, partial sequence	NR_126289	97	swallow cave in Yunnan
OTU0055	Uncultured bacterium clone IZ1RPV404EDZS2 16S ribosomal RNA gene, partial sequence	KP948749	99	intestines of the black tiger shrimp
OTU0056	Uncultured *Vibrio* sp. clone T028deg41 16S ribosomal RNA gene, partial sequence	JN210667	99	sponge tissue
OTU0057	*Acinetobacter* sp. HKG 224 16S ribosomal RNA gene, partial sequence	KU687329	99	soil sediments
OTU0058	*Massilia alkalitolerans* 16S ribosomal RNA gene, partial sequence	KX013423	99	desertic areas of South-East Morocco
OTU0059	Uncultured bacterium clone BIGO525 16S ribosomal RNA gene, partial sequence	HM558649	99	herbivore insect microbiome
OTU0060	Uncultured bacterium clone 071065_556 16S ribosomal RNA gene, partial sequence	JQ475196	99	human mouth
OTU0061	*Cobetia amphilecti* strain 25 16S ribosomal RNA gene, partial sequence	KX218313	99	male *Octopus* mucus
OTU0062	Uncultured bacterium clone HYPJBHH13H4NHH 16S ribosomal RNA gene, partial sequence	KF316017	99	hydraulic fracturing fluids and produced water from shale gas extraction
OTU0063	Uncultured bacterium clone ncd700e08c1 16S ribosomal RNA gene, partial sequence	HM291804	99	skin, nare
OTU0064	Uncultured bacterium clone IZ1RPV403CYXQV 16S ribosomal RNA gene, partial sequence	KP948057	99	intestinal flora of black tiger shrimp
OTU0065	Uncultured bacterium clone ncd1885c01c1 16S ribosomal RNA gene, partial sequence	JF162761	99	skin, antecubital fossa
OTU0066	Uncultured *Acinetobacter* sp. clone BER_e12 16S ribosomal RNA gene,	JQ815599	100	Tinto River acidic sediments

	partial sequence			
OTU0067	Uncultured bacterium clone G4RKN2Y15JDCSA	KC033337	99	soybean aphid
OTU0068	Uncultured bacterium clone GXTJ5A301BBJGP 16S ribosomal RNA gene, partial sequence	KF329567	98	intestinal flora of black tiger shrimp
OTU0069	Uncultured Rhodobacteraceae bacterium clone 1LM16-3 16S ribosomal RNA gene, partial sequence	JN232343	98	seagrass epiphytic biofilm
OTU0070	Uncultured *Gemella* sp. clone 074-194 16S ribosomal RNA gene, partial sequence	KU363169	99	oral cavity
OTU0071	*Halomonas* sp. U1368-101106-SW116	JQ082135	99	surface sea water
OTU0072	*Granulicatella adiacens* gene for 16S ribosomal RNA, strain: GM01	LC125190	99	human endophthalmitis patient
OTU0073	Bacterium 'H. scabra 1 (H-Fa)' 16S ribosomal RNA gene, partial sequence	JX022727	99	hindgut-feces
OTU0074	Uncultured bacterium clone I3Q1XXJ02CG1P3 16S ribosomal RNA gene, partial sequence	KP953479	98	intestinal flora of black tiger shrimp
OTU0075	*Shewanella baltica* strain I-M-3-2 16S ribosomal RNA gene, partial sequence	KU570317	99	intestinal tract and gills of Chinese mitten crab
OTU0076	Uncultured *Veillonella* sp. clone OTU_10559 16S ribosomal RNA gene, partial sequence	KR841843	99	soil of a tobacco plantation
OTU0077	Uncultured Pseudonocardiaceae bacterium clone Bo-Arch-K16 16S ribosomal RNA gene, partial sequence	KF767291	99	parchment
OTU0078	Uncultured *Aliivibrio* sp. clone O-A5 16S ribosomal RNA gene, partial sequence	HQ897484	99	intestinal microbiota of farmed Atlantic salmon *Salmo salar*
OTU0079	Uncultured *Saccharopolyspora* sp. clone VE21-K13 16S ribosomal RNA gene, partial sequence	KF767306	99	parchment

OTU0080	*Corynebacterium kroppenstedtii* strain VA5601_15 16S ribosomal RNA gene, partial sequence	KT347607	99	prevertebral abscess
OTU0081	Uncultured bacterium clone ncd2140c06c1 16S ribosomal RNA gene, partial sequence	JF184172	99	human skin, nare
OTU0082	*Fusobacterium* sp. Marseille-P2749 partial 16S rRNA gene, strain Marseille-P2749	LT576389	99	stool sample from hospital in Timone
OTU0083	Uncultured bacterium clone GXTJ5A301BTLUK 16S ribosomal RNA gene, partial sequence	KF333779	97	intestinal flora of black tiger shrimp
OTU0084	Uncultured prokaryote clone OTU_5702 16S ribosomal RNA gene, partial sequence	KT018789	100	heavy metal contaminated soil
OTU0085	*Actinomyces johnsonii* strain RSAA17 16S ribosomal RNA gene, partial sequence	KX298724	99	sheep
OTU0086	*Vibrio harveyi* strain ATCC 43516 chromosome 1, complete sequence	CP014038	99	shark
OTU0087	Uncultured bacterium clone ncd2677e07c1 16S ribosomal RNA gene, partial sequence	JF232588	99	skin
OTU0088	*Rothia mucilaginosa* strain DNF00207 16S ribosomal RNA gene, partial sequence	KU726646	99	vaginal fluid
OTU0089	*Pseudomonas oryzihabitans* strain H72 16S ribosomal RNA gene, partial sequence	KX390639	100	indoor environment
OTU0090	Uncultured *Vibrio* sp. clone 12L_112 16S ribosomal RNA gene, partial sequence	KP183078	99	surface sea water, China South Sea
OTU0091	Uncultured bacterium clone B4-2 16S ribosomal RNA gene, partial sequence	KF494503	98	permafrost soil
OTU0092	Uncultured *Corynebacterium* sp. clone B8-70 16S ribosomal RNA gene, partial	KF448111	99	water-flooded petroleum reservoirs

	sequence			
OTU0093	Uncultured *Aeromonas* sp. clone DGL42 16S ribosomal RNA gene, partial sequence	AY528791	99	activated sludge
OTU0094	*Kocuria rhizophila* strain Bss-9a 16S ribosomal RNA gene, partial sequence	KX161390	99	sediment from Badab-e Surt travertine spring
OTU0095	Uncultured bacterium clone p31_44-D06 16S ribosomal RNA gene, partial sequence	KX437605	99	plaque from teeth
OTU0096	*Saccharopolyspora* sp. AFM 10238 16S ribosomal RNA gene, partial sequence	KF673492	98	Dead Sea
OTU0097	*Pseudomonas luteola* strain LOCK 1011 16S ribosomal RNA gene, partial sequence	KT728842	100	textiles from 1250-1450 AC
OTU0098	Uncultured bacterium clone HYPJBHH15I4RDY 16S ribosomal RNA gene, partial sequence	KF316172	99	hydraulic fracturing fluids and produced water from shale gas extraction
OTU0099	Uncultured bacterium clone I3Q1XXJ01A7M8V 16S ribosomal RNA gene, partial sequence	KP953518	97	intestinal flora of black tiger shrimp
OTU0100	*Azospirillum formosense* strain UENF-411203	KU850954	99	tropical fruits crop
OTU0101	*Veillonella parvula* strain NRBB60 16S ribosomal RNA gene, partial sequence	KX130947	99	buffalo rumen liquor
OTU0102	Uncultured bacterium clone I3Q1XXJ02CAUFP 16S ribosomal RNA gene, partial sequence	KP952426	98	intestines of the black tiger shrimp
OTU0103	*Rheinheimera japonica* strain KP17 16S ribosomal RNA gene, partial sequence	NR_136858	99	seashore sediments of the Sea of Japan
OTU0104	Uncultured *Cetobacterium* sp. clone L.j-1 16S ribosomal RNA gene, partial sequence	HM031433	97	intestines of marine fishes
OTU0105	*Saccharopolyspora* sp. 13-18-42 16S ribosomal RNA gene, partial sequence	KM886189	98	sponge, South China Sea

OTU0106	*Saccharopolyspora* sp. 13-18-42 16S ribosomal RNA gene, partial sequence	KM886189	98	sponge, South China Sea
OTU0107	*Corynebacterium imitans* strain 58FIII 16S ribosomal RNA gene, partial sequence	KT832816	99	hospital objects and surfaces
OTU0108	Uncultured bacterium clone OTU_28873 16S ribosomal RNA gene, partial sequence	KU224876	99	freshwater biofilm
OTU0109	Uncultured *Vibrio* sp. clone GY29W4J02G4CEG 16S ribosomal RNA gene, partial sequence	KF116007	99	red palm weevil
OTU0110	*Aeromonas caviae* strain F1 16S ribosomal RNA gene, partial sequence	KP120939	98	natural waters
OTU0111	Uncultured *Neisseriales bacterium* clone E5VB3_96 16S ribosomal RNA gene, partial sequence	HM080824	100	prostatitis sample
OTU0112	Uncultured bacterium clone 16S(V3-V4)-865 16S ribosomal RNA gene, partial sequence	KX623641	99	rhizoshere soil
OTU0113	Uncultured *Saccharopolyspora* sp. clone VE21-K12 16S ribosomal RNA gene, partial sequence	KF767305	98	parchment
OTU0115	*Prevotellaceae bacterium* Marseille-P2826 partial 16S rRNA gene, strain Marseille-P2826	LT576392	99	human duodenum sample
OTU0116	Uncultured *Saccharopolyspora* sp. clone Bo-Arch-K40 16S ribosomal RNA gene, partial sequence	KF767296	99	parchment
OTU0117	Uncultured bacterium partial 16S rRNA gene, clone US11.34_V02193B_093	FN826506	98	cystic fibrosis patient sputum
OTU0118	Uncultured bacterium gene for 16S rRNA, partial sequence, clone: PT-MAG-B41	AB369028	100	petroleum crude oil in Asia
OTU0119	Uncultured bacterium clone I3Q1XXJ01A03QI 16S ribosomal RNA gene, partial sequence	KP949610	97	intestinal flora of black tiger shrimp

OTU0121	Uncultured Proteobacterium clone Upland_16_5276	JF985978	99	agricultural soil
OTU0123	*Vibrio harveyi* strain T4Y2 16S ribosomal RNA gene, partial sequence	JF520424	99	marine fish in Sabah
OTU0124	*Vibrio porteresiae* strain MSSRF30 16S ribosomal RNA gene, partial sequence	NR_044248	99	rhizosphere of *Porteresia coarctata* growing in a mangrove
OTU0126	Uncultured bacterium clone OTU_1450 16S ribosomal RNA gene, partial sequence	KU226295	99	soil
OTU0127	Uncultured *Saccharopolyspora* sp. clone VE21-K39 16S ribosomal RNA gene, partial sequence	KF767303	98	parchment
OTU0130	Uncultured bacterium clone IZ1RPV403DKA14 16S ribosomal RNA gene, partial sequence	KP948277	98	intestinal flora of black tiger shrimp
OTU0131	Uncultured Rubrobacteridae bacterium clone S3-013 16S ribosomal RNA gene, partial sequence	KF183142	98	soil of continuous peanut cropping
OTU0133	Uncultured *Erwinia* sp. clone GY29W4J02JHLJO 16S ribosomal RNA gene, partial sequence	KF115748	99	red palm weevil
OTU0134	Uncultured *Cetobacterium* sp. clone L.j-1 16S ribosomal RNA gene, partial sequence	HM031433	99	intestines of marine fishes
OTU0135	*Fusobacterium* sp. Marseille-P2749 partial 16S rRNA gene, strain Marseille-P2749	LT576389	98	stool sample from hospital in Timone
OTU0136	*Stenotrophomonas* sp. s1425 16S ribosomal RNA gene, partial sequence	KX085483	99	*Stenotrophomonas* sp. s1425
OTU0137	*Salinicoccus* sp. strain YM-15 16S ribosomal RNA gene, partial sequence	KX225419	99	Yuncheng salt lake
OTU0138	Uncultured Alphaproteobacterium clone Hc_OTU1	HM593540	99	sponge and corals
OTU0139	*Saccharopolyspora* sp. AFM 10238 16S ribosomal RNA gene, partial sequence	KF673492	97	Dead Sea

OTU0140	Uncultured *Moraxellaceae* bacterium clone SIM056 16S ribosomal RNA gene, partial sequence	JF733221	96	black flies
OTU0141	*Comamonas* sp. RCB175 16S ribosomal RNA gene, partial sequence	KT260387	99	bat guano
OTU0142	Uncultured bacterium clone H39KNTR01BZZ8U 16S ribosomal RNA gene, partial sequence	KF315360	99	hydraulic fracturing fluids and produced water from shale gas extraction
OTU0143	Uncultured *Saccharopolyspora* sp. clone Bo-Arch-K10 16S ribosomal RNA gene, partial sequence	KF767295	97	parchment
OTU0144	*Pseudomonas* sp. D2-17 partial 16S rRNA gene, isolate D2-17	AM403176	99	marine aquaculture biofilter
OTU0145	Uncultured *Saccharopolyspora* sp. clone MS492-K11 16S ribosomal RNA gene, partial sequence	KF767276	99	parchment
OTU0146	Uncultured *Saccharopolyspora* sp. clone VE21-K19 16S ribosomal RNA gene, partial sequence	KF767307	98	parchment
OTU0148	*Tannerella* sp. oral taxon 286 clone WWP_SS7_C16 16S ribosomal RNA gene, partial sequence	GU409361	99	oral cavity
OTU0149	Uncultured *Saccharopolyspora* sp. clone VE21-K19 16S ribosomal RNA gene, partial sequence	KF767307	97	parchment
OTU0150	*Stenotrophomonas* sp. s1425 16S ribosomal RNA gene, partial sequence	KX085483	99	*Stenotrophomonas* sp. s1425
OTU0152	Uncultured actinomycete clone Z0-72 16S ribosomal RNA gene, partial sequence	KJ834229	98	wheat straw mushroom compost
OTU0155	*Psychrobacter aestuarii* strain 0096 16S ribosomal RNA gene, partial sequence	KP236231	99	coastal area
OTU0156	Uncultured *Saccharopolyspora* sp. clone VE21-K19 16S ribosomal RNA gene, partial sequence	KF767307	98	parchment

OTU0157	*Azospirillum formosense* strain UENF-411203 16S ribosomal RNA gene, partial sequence	KU850954	98	rhizosphere soil
OTU0159	Uncultured bacterium clone G7DUZBG01AHBU7 16S ribosomal RNA gene, partial sequence	KF329420	99	intestinal flora *Penaeus monodon*
OTU0160	Uncultured bacterium clone I3Q1XXJ01A60NA 16S ribosomal RNA gene, partial sequence	KP953371	99	intestinal flora *Penaeus vannamei*
OTU0161	Uncultured bacterium clone JFR0502_aaa12f04 16S ribosomal RNA gene, partial sequence	HM779123	97	conventionally-raised adult zebrafish
OTU0163	Uncultured bacterium clone ncd2170d07c2 16S ribosomal RNA gene, partial sequence	JF189381	98	skin antecubital fossa
OTU0164	Uncultured bacterium clone I3Q1XXJ01AWXAX 16S ribosomal RNA gene, partial sequence	KP952197	98	intestinal flora *Penaeus vannamei*
OTU0165	*Saccharopolyspora* sp. MOLA 1509 16S ribosomal RNA gene, partial sequence	KM273921	98	wash water
OTU0168	Uncultured planctomycete clone Dover216A 16S ribosomal RNA gene, partial sequence	AY499825	97	marine sediment
OTU0169	*Saccharopolyspora* sp. AFM 10238 16S ribosomal RNA gene, partial sequence	KF673492	97	Dead Sea
OTU0172	Uncultured bacterium clone GXTJ5A301BC6H6 16S ribosomal RNA gene, partial sequence	JX934603	98	intestinal flora *Penaeus monodon*
OTU0174	*Saccharopolyspora* sp. AFM 10238 16S ribosomal RNA gene, partial sequence		98	Dead Sea
OTU0175	Uncultured bacterium clone nbw872c09c1 16S ribosomal RNA gene, partial sequence	GQ031338	99	skin, retroauricular crease
OTU0176	Uncultured bacterium clone ncd2062g05c1 16S ribosomal RNA gene, partial sequence	JF177605	99	skin, antecubital fossa

OTU0179	Uncultured bacterium clone I3Q1XXJ02B18KP 16S ribosomal RNA gene, partial sequence	KP953226	97	intestinal flora *Penaeus vannamei*
OTU0181	*Acinetobacter* sp. TUST-DFRC2 16S ribosomal RNA gene, partial sequence	KX390869	100	crude oil marine water
OTU0183	Uncultured bacterium clone 1_62_F08 16S ribosomal RNA gene, partial sequence	KX376534	100	mesh from abdominal
OTU0184	Uncultured bacterium clone p9_62 16S ribosomal RNA gene, partial sequence	KJ601253	97	seawater around coral
OTU0185	Uncultured bacterium clone 16S(V3-V4)-3783 16S ribosomal RNA gene, partial sequence	KX626448	98	rhizosphere soil
OTU0186	Uncultured Rhodobacteraceae bacterium 16S rRNA gene, clone W3R21	LT576206	99	roots of wheat plant
OTU0187	Uncultured bacterium clone HYPJBHH14IN8MC 16S ribosomal RNA gene, partial sequence	KF315822	98	hydraulic fracturing fluids and produced water from shale gas extraction
OTU0189	Uncultured bacterium clone 16S(V3-V4)-7891 16S ribosomal RNA gene, partial sequence	KX630383	99	rhizosphere soil
OTU0190	Uncultured bacterium clone GXTJ5A301BTPTV 16S ribosomal RNA gene, partial sequence	KF334052	98	intestinal flora *Penaeus monodon*

The best scoring sequence was chosen; whenever a score was shared by several sequences, the first sequence has been listed, possibly avoiding the ones referring to purified genomes.

Figure S1. Venn diagram of the OTUs found in the purple damaged (Purple) and in the uncoloured undamaged (White) samples.

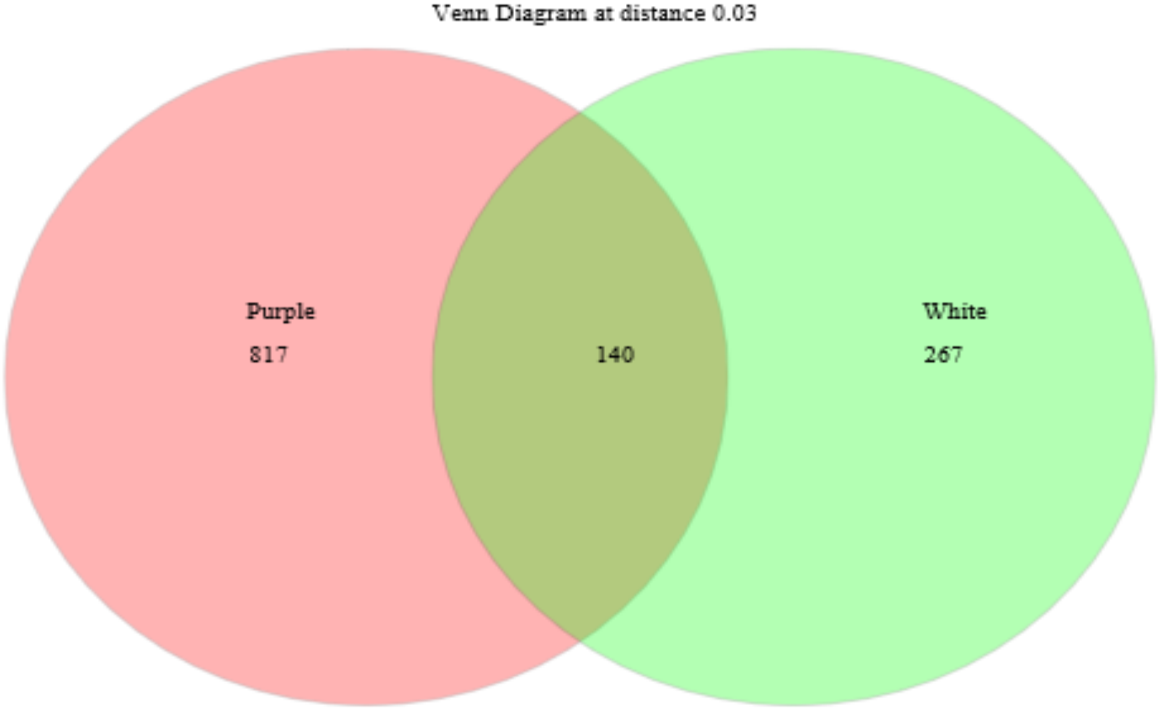

The number of species in group Purple is 957, and the number of squences is 11445; 1498 sequences are not shared

The number of species in group White is 407, and the number of squences is 5385; 382 sequences are not shared

The number of sepecies shared between groups Purple and White is 140, and the number of squences is 14950; 88.8295% of these sequences are shared

Percentage of species that are shared in groups Purple and White is 11.4379

The total richness for all groups is 1224
